# Image analysis workflows to reveal the spatial organization of cell nuclei and chromosomes

**DOI:** 10.1080/19491034.2022.2144013

**Published:** 2022-11-29

**Authors:** Ricardo S Randall, Claire Jourdain, Anna Nowicka, Kateřina Kaduchová, Michaela Kubová, Mohammad A. Ayoub, Veit Schubert, Christophe Tatout, Isabelle Colas, Kalyanikrishna, Sophie Desset, Sarah Mermet, Aurélia Boulaflous-Stevens, Ivona Kubalová, Terezie Mandáková, Stefan Heckmann, Martin A. Lysak, Martina Panatta, Raffaella Santoro, Daniel Schubert, Ales Pecinka, Devin Routh, Célia Baroux

**Affiliations:** a Department of Plant and Microbial Biology, Zürich-Basel Plant Science Center, University of Zürich, Zürich, Switzerland; b Institute of Biology, Freie Universität Berlin, Germany; c Centre of the Region Haná for Biotechnological and Agricultural Research (CRH), Institute of Experimental Botany, v. v. i. (IEB), Olomouc, Czech Republic; d Central European Institute of Technology (CEITEC) and Department of Experimental Biology, Masaryk University, Brno, Czech Republic; e Leibniz Institute of Plant Genetics and Crop Plant Research (IPK) Gatersleben, D-06466 Seeland, Germany; f Institut Génétique, Reproduction et Développement (GReD), Université Clermont Auvergne, CNRS, INSERM, 63001 Clermont-Ferrand, France; g The James Hutton Institute, Errol Road, Invergowrie, DD2 5DA, Scotland UK; h Central European Institute of Technology (CEITEC) and National Centre for Biomolecular Research, Masaryk University, Brno, Czech Republic; i Department of Molecular Mechanisms of Disease, DMMD, University of Zürich, Zürich, Switzerland; j Service and Support for Science IT (S3IT), Universität Zürich, Zürich, Switzerland

**Keywords:** Nucleus, chromatin, 3D organization, spatial distribution, image analysis, segmentation, quantification, mitosis, meiosis, chromosome, metaphase, pachytene, crossovers, nuclear speckles, nuclear bodies, RNA Pol II, transcription factories, oligo FISH, STED imaging, SIM

## Abstract

Nucleus, chromatin, and chromosome organization studies heavily rely on fluorescence microscopy imaging to elucidate the distribution and abundance of structural and regulatory components. Three-dimensional (3D) image stacks are a source of quantitative data on signal intensity level and distribution and on the type and shape of distribution patterns in space. Their analysis can lead to novel insights that are otherwise missed in qualitative-only analyses. Quantitative image analysis requires specific software and workflows for image rendering, processing, segmentation, setting measurement points and reference frames and exporting target data before further numerical processing and plotting. These tasks often call for the development of customized computational scripts and require an expertise that is not broadly available to the community of experimental biologists. Yet, the increasing accessibility of high- and super-resolution imaging methods fuels the demand for user-friendly image analysis workflows. Here, we provide a compendium of strategies developed by participants of a training school from the COST action INDEPTH to analyze the spatial distribution of nuclear and chromosomal signals from 3D image stacks, acquired by diffraction-limited confocal microscopy and super-resolution microscopy methods (SIM and STED). While the examples make use of one specific commercial software package, the workflows can easily be adapted to concurrent commercial and open-source software. The aim is to encourage biologists lacking custom-script-based expertise to venture into quantitative image analysis and to better exploit the discovery potential of their images.

**Abbreviations:** 3D FISH: three-dimensional fluorescence in situ hybridization; 3D: three-dimensional; ASY1: ASYNAPTIC 1; CC: chromocenters; CO: Crossover; DAPI: 4',6-diamidino-2-phenylindole; DMC1: DNA MEIOTIC RECOMBINASE 1; DSB: Double-Strand Break; FISH: fluorescence in situ hybridization; GFP: GREEN FLUORESCENT PROTEIN; HEI10: HUMAN ENHANCER OF INVASION 10; NCO: Non-Crossover; NE: Nuclear Envelope; Oligo-FISH: oligonucleotide fluorescence in situ hybridization; RNPII: RNA Polymerase II; SC: Synaptonemal Complex; SIM: structured illumination microscopy; ZMM (ZIP: MSH4: MSH5 and MER3 proteins); ZYP1: ZIPPER-LIKE PROTEIN 1.

## Introduction

Elucidating the spatial organization of eukaryotic genomes, their structural and compositional dynamics during cellular processes and functional relationship with the nucleus, is a keystone of three-dimensional (3D) genomics. 3D genomics aims to decipher the functional, 3D organizing principles of the chromosomes, chromatin domains and nucleus that contribute to transcription, replication, repair, and recombination. Understanding the 3D genome requires multidisciplinary methods including high-throughput, sequencing-based, molecular profiling techniques, computational simulation-based biophysical and mathematical modeling, and microscopy imaging at high-to-super resolution and in three-dimensions [1,2]. Microscopy followed by image analysis provides the opportunity to measure chromosome and chromatin structures down to the nanoscale, with a few kilobase resolution. This can inform on the genomic interactions *in situ* and the spatial organization of genomic domains in relation to the 3D nuclear space and its functional compartments [3,4].

Venturing into these opportunities to probe for the spatial organization of the genome *in situ* requires dedicated imaging and image analysis procedures, recently captured by the concept of quantitative, data-driven microscopy [1]. Quantitative image analysis for nuclear and chromosomal studies can be implemented at different levels of complexity, depending on the research question and, often, the expertise available. For instance, a simple level consists of scoring structures or patterns on the image based on user-defined classification. This can be applied when the immunolabelled chromatin protein, or FISH-labeled genomic domain, shows a very distinct distribution pattern (e.g. punctuate vs diffuse), varying between treatments or genotypes. In this case, quantifying the relative occurrence of pattern categories by scoring may be sufficient to address the original question. Manual scoring can also be used to quantify a moderate number of labeled regions (e.g., number of FISH signals or nuclear bodies). These categorical, quantitative approaches have the virtue to be accessible to all experimentalists, without sophisticated software. They allow to characterize relatively simple signal distribution patterns, providing, however, a limited number of samples, and double-blind scoring to avoid cognitive biases. Yet, for many images (e.g., from high-throughput imaging), images with multiple labels, showing complex spatial patterns of signal distribution, with continuous (rather than discrete) variation in signal abundance, or a combination of all, require computationally driven processing approaches for quantitative analyses. A core step required is image segmentation. This process partitions the image based on the signal distribution into digital objects identifying biologically relevant structures. Various image segmentation methods and algorithms exist. These perform differently depending on the signal distribution [5], with deep-learning approaches for automated segmentation tasks at a large scale being continuously developed [6]. Once the image is segmented, multiple features can be extracted from the 3D digital objects, for instance, object number, size and shape; signal intensity and variance per object type, texture of the signal, channel and position in the image; distance relationships, and spatial distribution. Practically, these features are highly relevant to analyze the spatial organization of chromatin, chromosome and nuclear components *in situ*.

The field of chromatin, chromosome and nuclear organization studies would greatly benefit from the broader deployment of image processing-based quantitative analyses [2,3]. Several tools and packages have been developed in the past years based on open-source software, including for the 3D spatial analysis of nuclear organization [7–11]. Yet, a major hurdle for most ‘biology-only’ oriented labs is the lack of computational expertise for customizing the image processing scripts, for large data handling, the lack of template workflows, or a combination thereof. Key concepts, from image acquisition to quantitative data, have been framed in recent years, for applications in cell biology, but also to set good practice and standards in the field [1,12]. Efforts are undertaken to promote education and support in image analysis for scientists dealing with biological images [13]. This resource paper contributes to these efforts by providing a compendium of image analysis workflows for nucleus, chromatin and chromosome studies, taking seven case-studies as examples developed by participants of the training school of the INDEPTH COST action [14].

The workflows are based on a user-friendly, commercial image processing software (Imaris, Bitplane, Switzerland) but are conceptually applicable to concurrent (commercial or open source) software as discussed in this paper. In addition, although they largely borrow examples from plant nuclei and chromosomes, they remain transferable to the study of animal nuclei. Indeed, the organization of the nucleus, including the nuclear envelope, chromatin domains and chromosomes, share common organizing principles in plants and animals [15–18]

The workflows associated with each case study are briefly described below and are illustrated in the related figures. Each workflow is associated with a Supplemental File folder that includes a step-by-step guideline (text); a table summarizing the main step functions and parameters used on the training image; one or two training images per workflow; and, for workflow 1, a video tutorial. Training image datasets are available on the INDEPTH-OMERO repository [13,14].^1^

### Analyzing the spatial distribution of transcription clusters

In mammals, a radial gradient model of transcription in the nucleus has been proposed [19,20]. In plants, including the *Arabidopsis thaliana* (Arabidopsis) plant model, little is known about the spatial, 3D distribution of transcription. Transcriptional activity in the nucleus can be visualized *in situ* by immunolabeling the active isoform of RNA Polymerase II (RNPII). In Arabidopsis, super-resolution imaging of RNPII has shown a reticulate pattern throughout the nucleoplasm along which distributed clusters of variable size and intensity exist [21,22].

**Figure 1.**
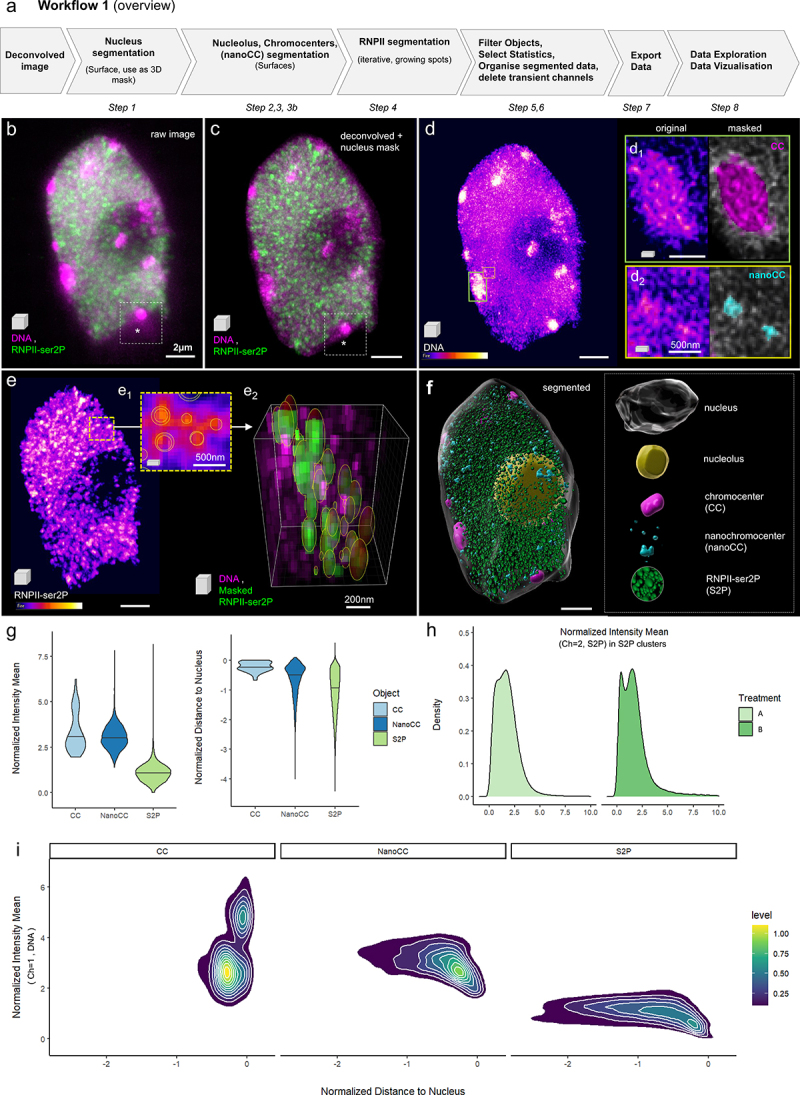
**Analysis of the spatial distribution of RNA Pol II clusters in intact nuclei. (a)** Overview of the workflow illustrated in b-i; **(b)** 3D projection of a 3D-STED image reporting on immunolabelled RNA Pol II (isoform phosphorylated on SerP, green, RNPII-ser2P) and DNA (magenta, Hoechst 580CP [26]), raw image; **(c)** Same image following deconvolution, nucleus contour segmentation and masking; **(d)** Intensity-coded coloring mode (Fire) of the DNA channel and frames magnified in the insets showing examples of chromocenters (CC, d1) and nanochromocenters (nanoCC, d2) in the original channel (left) and after segmentation and pseudo-coloring (right); **(e)** Intensity-coded coloring mode (Fire) of the RNPII-ser2P channel showing a dense distribution of clusters with identifiable intensity peaks, enabling segmentation as adaptive spots (e1, e2), e1: single plane showing the spot contours; e2, 3D segment of the image after segmentation, clusters pseudo-colored in green, DNA in magenta. **(f)** Fully segmented image containing surface (nucleus, nucleolus, CC and nanoCC) and spot objects (RNPII-ser2P, abbreviated S2P). **(g-i)** Data exploration using *DataViz* (github.com/barouxlab/DataViz, Supplemental File 1- Dataviz_guidelines). **(g)**, Violin plots showing a similar DNA density distribution in CC and nanoCC but much lower density in S2P clusters (intensity mean, DNA channel, normalized per image) and a sharp peripheral location of CC as formerly described (Andrey et al., 2010; Fransz et al., 2002), contrasting with the more dispersed distribution of nanoCC and S2P clusters (distance to nucleus surface (0) normalized using the nucleus center of mass as reference);.**(h)** Example showing an application of the workflow, to compare the distribution of RNPII cluster intensities between two treatments: A and B. **(i)** Another example illustrating one of the many analyses enabled by the workflow and DataViz, with density scatter plots of DNA intensity means in RNPII clusters as a function of their distance to the nucleus surface. Scale bars: b-f, 2 µm; insets, as indicated.

To resolve the spatial distribution of RNPII signals in Arabidopsis nuclei in 3D, we imaged RNPII and DNA using 3D-STED microscopy. To quantify RNPII foci distribution, we designed an image analysis workflow (Figure 1a and Supplemental File 1). Sample preparation and imaging are described elsewhere [23]. Deconvolved STED images are segmented using the Imaris software (Bitplane, Switzerland) to create digital objects corresponding to the nucleus, the nucleolus, the chromocenters and the RNPII signals (Figure 1b-d, Supplemental File 1 – Video 1). The surface object corresponding to the nucleus is also used to apply a 3D mask to separate the true image from the background signal (compare the framed regions in Figure 1b-c). While the nucleus and nucleolus are segmented based on smoothed, manual contours, heterochromatin is segmented using the supervised automated tool. Chromocenters (CCs) are typically large, brightly stained regions. In Arabidopsis nuclei, these are discrete and relatively easy to segment (Figure 1d, inset d1). Super-resolution imaging revealed that additional heterochromatin regions, which we termed nanochromocenters (nanoCC), can also be segmented (Figure 1d, inset d2). RNPII signal shows a complex nuclear distribution in Arabidopsis nuclei: rather than being discrete, it spreads unevenly in a reticulated manner with, however, clearly identifiable local clusters [22]. Our aim was to segment the image to discretize RNPII signal and focus on the clusters, considering their variable size, to further analyze their variability in intensity, size and spatial distribution. We applied the growing spot function in an iterative manner and could capture 70–80% of the RNPII signal in spots of variable size (Figure 1e, inset e2). This stepwise segmentation resulted in a digital image composed of objects capturing the nucleus, the nucleolus, the chromocenters and RNPII clusters (Figure 1f). Variables of interest, such as signal intensity per channel, object size and shape and distance between objects (spot-to-spot, spot-to-surface) were exported for each object type and channel.

The high number of variables, object type, channels, image replicates and levels of comparison (such as genotypes and treatment) dramatically increases data complexity. To facilitate data exploration, we built a stand-alone data visualization interface named *DataViz* (https://github.com/barouxlab/DataViz) which allows one to interactively plot all, or a subset of, data. This also enables custom variable creation for the normalization of distances and intensity per image (Figure 1g-i, Supplemental File 1 – Dataviz_guidelines). Here, we provide a few examples of violin plots (Figure 1g), density distributions (Figure 1h) and scatter plots with density contours (Figure 1i). The mean intensity, normalized per nucleus, of the DNA signal shows that the average and range of chromatin compaction in CC and nanoCC is largely similar, while nanoCC occasionally shows a higher compaction (upper tail of the violin plot, Figure 1g). By contrast, and as expected for transcriptionally active regions, chromatin is, on average, 2–3x less condensed in the RNPII-S2P clusters (Figure 1g). Also, plotting the shortest distance of each object to the nucleus surface (Figure 1g, right plot: the negative values indicate distance toward the nucleus interior), confirms the peripheral localization of CC as already described [24,25], an apparent enrichment of the nanoCC toward the periphery (although less pronounced than CC), and spreading of the RNPII-S2P clusters from the periphery toward the nuclear interior (with an apparent decreasing occurrence linked to the presence of the nucleolus). Further, plotting the density distribution of RNPII-S2P signals in the clusters (normalized mean intensity) allows detecting different structures of the RNPII landscape between different treatments (A and B in the example provided Figure 1h). Finally, DataViz enables exploring the relationship between two continuous variables using scatter plots, with or without density contours. In the example provided Figure 1i, we interrogated the relationship between the distance to the nucleus boundary and the mean DNA intensity for each of the nuclear domains segmented as CC, nanoCC and S2P clusters. The plots suggested (i) two categories of CC distinctive mostly by their intensity but slightly different with regard to their peripheral position and that (ii) nanoCC and transcription clusters closer to the periphery are on average less compact than their counterparts located more toward the nuclear interior. These are only a few examples of the numerous possible plots that collectively contribute to data mining and discovery.

The segmentation process described in detail in the supplemental material corresponds to a user-supervised workflow. The input values (threshold, smoothing factor, or filtering values) are either software-defined values (and depend on image attributes) or customized by the user to best capture the biological objects. The parameters are then saved and re-applied for subsequent image replicate analyses. If the image quality is highly reproducible, it is further possible to apply automated batch-segmentation (following the software provider’s instructions). For a trained user, the workflow takes *ca*. 45 min per image or less. Finally, this workflow can be further applied for the analysis of other types of nuclear signals showing punctate distribution similar to that in our example.

### Analysis of the spatial distribution of proteins located at the nuclear periphery

To date, the distribution of nuclear envelope (NE)-associated proteins is poorly documented in plants. 3D microscopy-based observations may provide new insights into the organization of chromatin domains at the nuclear periphery at the single-cell level.

**Figure 2.**
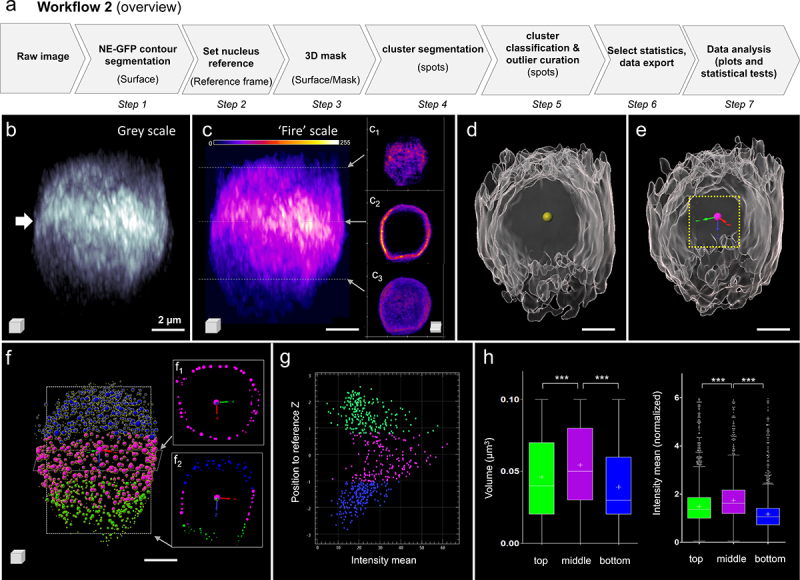
**Analysis of the spatial distribution of a fluorescently tagged protein associated with the nuclear envelope**. (a) Overview of the Image analysis workflow. Details of the parameters are in supplements. (b) Raw image of NE-GFP (Nuclear Envelope – associated protein fused to GFP) signal in a root nucleus; 3D rendering in gray levels suggests an enrichment of the protein at the equatorial region of the nucleus (white arrow). (c) Same image (3D) as in (b) using a fire color scale for NE-GFP signal intensities display (0–255), c1-c3 insets: cross sections at selected top, middle and bottom planes, respectively. (d) Result of the segmentation of the NE-GFP signal domain as a surface (gray); a spot (yellow) is created at the surface’ center-of-mass. (e) a new XYZ coordinate system (reference frame) is docked at the center-of-mass. (f) The NE-GFP signal is segmented as spots of adaptive size (‘growing spots’) using the channel masked by the surface; spots are classified according to their axial (z) position, the equatorial region is defined ±2 µm around the origin. Three spot classes are created located at the top, middle and bottom of the nucleus (blue, magenta, green, respectively). f1, f2 insets: XY and XZ sections. (g) The intensity mean of the spots is plotted as a function of their axial position (z) relative to the new reference frame for the image shown in (b-f). The colors indicate the ‘top’, ‘middle’ and ‘bottom’ classes, respectively. (h) The volume and normalized intensity mean of NE-GFP spots are plotted for each class, for n = 8 nuclei images segmented following this workflow. Kruskal-Wallis and Dunn’s multiple comparison tests with bottom vs middle and top vs middle indicate statistically significant differences with P < 0.001 (***) for both variables. Scale bars: 2 µm.

In this example, we developed a workflow to quantify the spatial pattern of a protein heterogeneously distributed within the NE (Figure 2a). We imaged Arabidopsis root nuclei expressing a GFP-tagged protein associated with the NE (NE-GFP, *Tatout, Mermet, Boulaflous-Stevens, unpublished*) from 1 week old seedlings using a confocal microscope equipped with an Airyscan module [27] (Supplemental File 2- Figure 2). The NE-GFP protein is located at the NE and forms clusters of variable size; these clusters appear to be asymmetrically distributed (arrow, Figure 2b). Intensity-based coloring of the signal confirmed the enrichment of NE-GFP at the equatorial plane of the nucleus, in contrast to that at the top and bottom poles (Figure 2c and insets). The first step in our procedure was to segment the global domain of NE-GFP signal using the ‘Surface’ function of Imaris (Figure 2d). Subsequently, we created a spot at the center of mass of the segmented NE-GFP surface (yellow spot, Figure 2d) and used it to create a ‘Reference frame’ object (Figure 2e). This new XYZ coordinate system at the nucleus’ center allows to classify the NE-GFP clusters at a later stage. The NE-GFP clusters are segmented as spots of adaptive size on the NE-GFP domain masked on the surface created at step 1 (Step 3–4, Supplemental File 2). Spots were classified into three categories (top:blue, middle:magenta, bottom:green) according to their axial position in the coordinate system defined at step 2 (Step 5, Figure 2f). The ‘middle’ class is defined by a region encompassing the origin of this coordinate system from −2 to +2 µm along the z-axis. The ‘top’ and ‘bottom’ classes capture the spots above and below this equatorial region, respectively. In addition, this step included curation of the segmentation results to (i) keep spots strictly located at the periphery (removing outliers located internally due to surface invaginations) and (ii) to select spots of biologically relevant size (up to 600 nm diameter, ~1.1 µm^3^; detailed procedure in Supplemental File 2). A 2D-plot of the mean intensity of segmented NE-GFP spots according to their axial (x) position in this coordinated system revealed higher signal intensity among spots located at the equatorial plane (‘middle’ class, Figure 2g). The segmentation of multiple images supported this finding (Figure 2h). Importantly, as fluorescence intensities varied between images, the mean intensity of each spot was normalized, following export, using the mean intensity within the NE-GFP surface for each image (Figure 2h). This analysis revealed that both the volume and mean intensity of NE-GFP clusters in the equatorial plane (‘middle’ class) are significantly different from that of the clusters located at the polar regions (‘top’ and ‘bottom’ classes; Figure 2h, Kruskal-Wallis and Dunn’s multiple comparison test with P < 0.0001 for all pairs).

In conclusion, this image analysis workflow enables quantification of the spatial heterogeneity of proteins associated with the nuclear envelope. In combination with mutant genetics, this approach enables one to assess the quantitative influence of candidate regulators and that of intrinsic (protein) domains on spatial protein localization.

### Analysis of protein distribution on meiotic chromosomes

Meiosis is a special type of cell division occurring during sexual reproduction and enabling genetic recombination. During the first stage of meiosis, prophase I, homologous chromosomes align along their entire length by a protein structure called the synaptonemal complex (SC). This process is essential for crossover (CO) formation in many eukaryotes. Prophase I is itself divided into five substages – leptotene, zygotene, pachytene, diplotene and diakinesis. Each stage can be monitored by immunostaining specific proteins involved in SC formation. The most common targets are ASY1 (ASYNAPTIC 1) and ZYP1 (ZIPPER-LIKE 1) [28]. During prophase I, homologous recombination starts with the formation of SPO11-programmed DNA double-strand breaks (DSB) [29]. These DSBs are subsequently processed and recombinases such as DMC1 (DNA MEIOTIC RECOMBINASE 1) mediate strand invasion, essential for CO formation [30]. In barley, a large number of DSBs are formed [31], but only 13–22 (depending on the cultivar and scoring method) are repaired to crossover (CO), while the rest are repaired as non-crossovers (NCO) [32,33]. What controls the fate of DSB (CO vs NCO) is poorly understood. A current hypothesis involves HEI10 (HUMAN ENHANCER OF INVASION 10), a ZMM class-of-protein in the CO repair pathway, as an early indicator [34].

To elucidate whether HEI10 also contributes to DSB fate designation in barley, one approach is to elucidate the dynamics of HEI10 foci along prophase chromosomes at early, mid, and late stages, and in relation to DMC1 at early prophase. This approach requires 3D imaging of (immunostained) meiotic proteins on prophase chromosomes and the scoring of HEI10 vs DMC1 foci in relation to the prophase stage. We describe here a workflow to process 3D-SIM images to enable the scoring and classification of DMC1 and HEI10 foci depending on their size and intensity.

**Figure 3.**
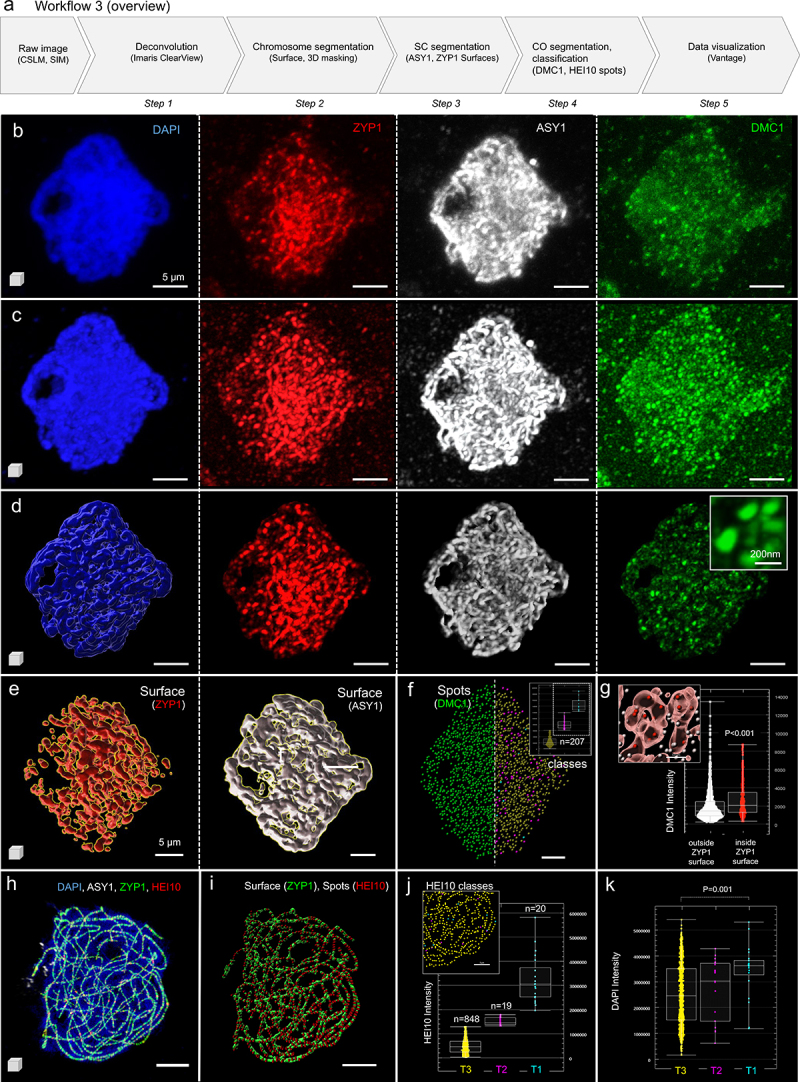
**Analysis of crossover distribution in meiocytes**. (a) Overview of the image analysis workflow in 5 steps illustrated on two images marking the synaptonemal complex (SC) and crossovers (CO) with different components (provided in Supplemental File 3): image 3a (b-g) represents a barley meiocyte at zygotene stage immunostained for DMC1, ZYP1 and ASY1 and counterstained for DNA using DAPI. The image was acquired by confocal microscopy (ZEISS LSM 710) as described (Colas et al., 2019). Image 3b (h-k) represents a barley meiocyte at the late pachytene stage immunostained for ASY1 (Ch = 2), ZYP1 (Ch = 3) and HEI10 (Ch = 4) and counterstained for DNA using DAPI (Ch = 1). The image was acquired by 3D-SIM as described previously (Hesse et al., 2019). (b) Original image acquired by confocal imaging, the different labeling are indicated. (c) image following deconvolution to resolve the SC and immunostained CO. (d) segmentation of the chromosomes as surface and masking of the ZYP1, ASY1 and DMC1 channels to remove background signal. It allows resolving DMC1 foci at high resolution (inset). (e) SC segmentation using the ZYP1 and ASY1 masked channels (f) DMC1 foci segmentation (left) and classification according to their intensity (right and inset = intensity plot per category), (g) classification of DMC1 foci according to their distance relative to the ZYP1 surface. (h) Original 3d SIM image (image 3b), (i) same image following ZYP1 and HEI10 segmentation, (j) HEI10 spots were classified according to their intensity (T1, T2, T3 on graph and inset); 20 foci were scored (automatic) for the T1 class as described in earlier studies, (k) HEI10 classes differ by the DNA density. Scale bars: 5 µm except for the inset d, DMC1 channel (200 nm), Plots (f, g, j, k): Imaris Vantage.

The workflow (Figure 3a) is illustrated with two images of barley male meiocytes labeled for components of the SC (ASY1, ZYP1), processed DSBs (DMC1) and recombination intermediates (HEI10). The first image, shown in Figure 3b-g and provided in Supplemental File 3 – Image 3a, shows a zygotene stage nucleus labeled with ASY1 (white), ZYP1 (red), DMC1 (green) and counterstained with DAPI (blue), was acquired on a confocal microscope (Zeiss LSM 710) as per Colas *et al*. [33]. The second image, shown in Figure 3h-k and provided in Supplemental File 3 – Image 3b, shows a pachytene stage nucleus labeled with ASY1 (white), ZYP1 (green), HEI10 (red) and counterstained with DAPI (blue), was acquired using 3D-SIM similar as for rye [35]. The aim of both images was to segment HEI10 or DMC1 foci and to analyze their distribution relative to the SC, their size and their intensity. For this, the chromosomes, SC complex and HEI10 or DMC1 foci are segmented separately. The detailed strategies used for filtering and classifying the HEI10 or DMC1 foci are explained in the detailed workflow descriptions (Supplemental Files 3). In brief, the image analysis followed the steps of deconvolution (optional depending on the imaging method), chromosome segmentation, SC segmentation, CO foci segmentation and classification before data export (Figure 3a).

The effect of deconvolution is shown for the first image acquired by confocal microscopy to reconstruct the image at optical resolution (compare the panels Figure 3b and 3c). This step is essential for properly estimating the CO diameter later (Figure 3d, DMC1 channel inset). Next, the DNA (DAPI) staining was used to generate a 3D surface of the chromosomes serving as a mask to remove signal noise in the image (compare the panels Figure 3c and 3d). Note that here, the aim was not to segment the chromosomes very precisely, as the masking would result in the exclusion of ASY1, ZYP1 and DMC1 foci that do not entirely colocalize with DNA at that meiotic stage. Hence, permissive criteria were preferred in this case. Next, the SC complex was segmented on both the ASY1 and ZYP1 channels, creating two distinct surfaces (Figure 3e, red: surface ZYP1, white: surface ASY1). Finally, DMC1 foci were segmented as spot objects using an estimated seed size of 200 nm (Figure 3f). The algorithm detects all possible foci with both low and high intensities. Classical studies have so far focused on high-intensity foci, whose abundance falls within a few hundred [31,33]. By contrast, the workflow described here enables one to capture all foci, first, irrespective of their intensity, and to classify them according to intensity, during the creation process (Figure 3f, right panel). In this example, three classes were created (Figure 3f plot, yellow, magenta and cyan classes). Alternatively, spots can be classified after data export based on normalized signal intensity in a third-party software application (for instance, using DataViz, see Workflow1). In an intensity sum-based classification, we scored 217 DMC1 spots with medium-to-high intensity (Figure 3f plot, magenta and cyan classes) as previously reported for a similar stage of meiosis [31,33]. The remaining low-intensity spots (Figure 3f plot, yellow class) may correspond to either immunolabeling noise or unbound proteins. Next, we asked whether DMC1 localization was correlated with the SC. Indeed, following the classification of DMC1 spots in two groups, inside or outside the ZYP1 surface, we found a significant enrichment of DMC1 signal (based on intensity mean) when foci colocalize with ZYP1 (Figure 3g). This is one of the many examples of correlative analyses that can be carried-out in such segmented images.

The second image (Figure 3h) was analyzed similarly, but deconvolution, chromosome segmentation and masking were omitted in this case. The ZYP1 surface (Figure 3i, green) was used to mask the HEI10 channel to specifically focus on HEI10 foci (Figure 3i, red) colocalizing with ZYP1. HEI10 spots objects were then classified according to their intensity, considering notably the first and next 2% quantile versus the rest to create three classes, T1, T2 and T3, respectively (Figure 3j). This approach was formerly described to analyze CO distribution during meiosis in a fungal species [36]. The surprisingly high number of low-intensity HEI10 foci (T3 class) detected by the segmentation in this late pachytene-stage nucleus suggests the need for further investigation to understand their nature and possible function. To further describe the properties of HEI10 classes, we investigated different relationships and found that in most cells, typically high-intensity HEI10 foci (T1) localize, on average, on chromosome regions with higher DNA compaction (DAPI mean intensity) compared to low-intensity HEI10 foci (T3) (Figure 3k).

This image processing workflow facilitates the scoring of class I CO and NCO foci across multiple images, stages and genotypes, a task largely done manually until now. In addition, segmentation is near-exhaustive and includes low-intensity foci that were discarded from manual scoring in former studies. This raises the question of the dynamics of HEI10 and DMC1 foci formation, with possible intermediate stages represented by low-intensity foci. In addition, it opens the possibility to refine the analysis of CO/NCO spatial organization and their fine-scale structure. For instance, the localization of CO/NCO foci can be measured relatively to the SC components as a function of their intensity, and as a function of local chromatin compaction.

### Analysis of nuclear speckle distribution

A distinguishing feature of nuclear topography is the ability to accommodate a variety of subnuclear compartments including nuclear bodies. Nuclear bodies are membraneless compartments that spatially partition the nuclear environment and are thought to facilitate enzymatic reactions [37,38]. Similar to membrane-bound organelles, they maintain an effective steady-state structure, but likely by different mechanisms [39]. The first-identified and best-characterized plant nuclear bodies are the nucleolus and Cajal bodies. Several other smaller structures have, however, also been identified, including speckles, paraspeckles, coiled bodies and photobodies [40–42]. Unmasking the mechanisms by which cells assemble, maintain and regulate nuclear bodies and speckles, and the environmental and developmental factors contributing to the process, will shed light on their biological functions. For instance, splicing regulator (SR) proteins in plants localize as speckles, the size and shape of which are dependent on cell type, metabolic state and transcriptional activity [41–43].

One way to elucidate the speckle dynamics of nuclear bodies, which are not membrane-bound, is through microscopy imaging and image analysis. This approach enables the analysis of their spatial distribution and their composition relative to other nuclear components and DNA (chromatin) density. In this example, we showcase a simple image analysis workflow for analyzing the distribution of nuclear speckles and bodies. We used two images: Supplemental File 4 – image 4a reports on the nuclear localization of a plant chromatin remodeler: a SWI/SNF subunit (called SSSU here) forming nuclear speckles in leaf nuclei. Supplemental File 4 – image 4b reports on the nuclear localization of a mammalian chromatin protein (here called CP) and of H3K27me3 forming large nuclear bodies in nuclei of mouse naive pluripotent embryonic stem cells [44]. CP is a Baz-related subunit of the ISWI (Imitation SWItch) family remodeling complex factor, interacting with SNF2H, a SWI/SNF related remodeler [45] (Santoro, Panatta, unpublished).

**Figure 4.**
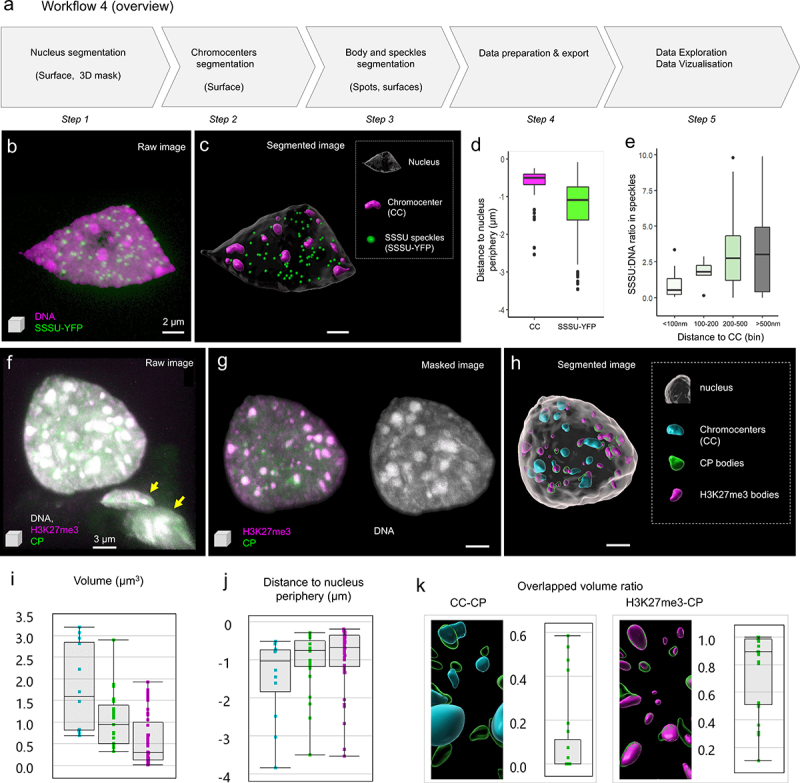
**Analysis of the spatial distribution of nuclear speckles and bodies**. (a) Overview of the image analysis workflow, details and training images are provided in Supplemental Files 4. The analysis of two images (Supplemental File 4 – image 4a and 4b) representing plant and animal nuclei are shown in (b-e) and (f-k), respectively. (b) Raw, STED image (3D projection) showing an isolated leaf nucleus stained for DNA (magenta, Hoechst 580CP [26],) and immunostained for SSSU (green). (c) Segmentation result: the nucleus, chromocenters (CC) and the nuclear speckles (SSSU) were segmented as surface objects (legend, right panel). (d) The position of CC and SSSU speckles was plotted relative to the nucleus’ periphery defined by the surface’s boundary (0 = at the boundary; negative values = toward the interior), n = 9 nuclei analyzed. (e) The relative enrichment of SSSU on chromatin was plotted as the SSSU:DNA mean signal intensity ratio for different classes of speckles defined by their distance to CC (in µm). Plots were generated using Dataviz (see Workflow 1) using data from n = 10 segmented nuclei. (f) Confocal image (3D projection) of a nucleus from a mouse naïve pluripotent embryonic stem cell stained for DNA (gray, DAPI), immunostained for the chromatin protein under study (CP, green) and H3K27me3 (magenta) forming large nuclear bodies; the arrows show truncated nuclei in the field of view undesirable for downstream analyses and eliminated upon masking at the next step. (g) Same image after 3D masking using the nucleus surface created at step 1. (h) Results of image segmentation: the nucleus, chromocenters (CC) and the nuclear bodies (CP and H3K27me3) were segmented as surface objects (legend, right panel). (i-k) Quantitative analysis of CC and nuclear bodies: volume: (i) distance to the nucleus periphery (j) and overlapping volume ratios (k, left: CC and CP overlap, right: CP and H3K27me3 overlap). Plots were generated using Imaris Vantage. Scale bar: (a-b), 2µm; (f-h), 3 µm.

SSSU was found to interact with PWO1 and CRWN1 (Kalyanikrishna, Jourdain, Schubert, unpublished), a set of proteins involved in epigenetic gene regulation and chromatin organization at the nuclear periphery [46]. CRWN1 (CROWDED NUCLEI 1), a nuclear lamina candidate in Arabidopsis, interacts with PWO1 (PROLINE-TRYPTOPHANE-TRYPTOPHANE-PROLINE INTERACTOR OF POLYCOMBS1), a plant-specific protein associated with histones and PRC2 (POLYCOMBREPRESSIVE COMPLEX 2) [46]. Due to its possible interaction with CRWN1, we asked whether SSSU is also located preferentially at the nuclear periphery. To answer this question, we tagged SSSU with YFP (YELLOW FLUORESCENT PROTEIN) and imaged nuclei expressing SSSU-YFP using STED microscopy. The image analysis workflow consists of only a few steps (Figure 4a): STED images reporting on the immunolabeled SSSU-YFP and DNA counterstaining (Figure 4b) were segmented for the nucleus, chromocenters (CC) and SSSU-speckles, using the surface tool. For segmentation of the nucleus, smooth, manual contours were used, while for segmenting CCs, automated, parameter-controlled settings were applied. SSSU speckles were segmented as spot objects of ca. 200 nm diameter. Segmentation data of several images were exported and plotted using DataViz (see workflow 1, Supplemental File 1 – Dataviz_guidelines). SSSU-YFP speckles showed a broad spatial distribution, with no clear preferential enrichment toward the periphery (Figure 4d), in contrast to chromocenters as previously shown [24,25]. We found, however, that SSSU speckles are not uniform: they differ in their relative enrichment (SSSU:DNA ratio), which correlates with the proximity to CC (Figure 4e). Our analysis demonstrates that the nuclear speckles formed by SSSU are not preferentially enriched at the nuclear periphery as would have been expected from their biochemical interaction with CRWN1. The analysis suggests a differential enrichment depending on the proximity to other nuclear bodies, the CC, a relationship whose functional relevance remains to be investigated. This preliminary finding was unexpected and was revealed thanks to the possibility to explore multiple relationships between distance and intensity measurements in DataViz using segmentation data generated using this workflow.

In the second example, we were interested in the CP protein localization relative to the repressive nuclear compartments formed by heterochromatin (chromocenters) and H3K27me3 in nuclei of mouse naive pluripotent embryonic stem cells. Nuclei stained for DNA and immunostained for CP and H3K27me3 were imaged at high resolution by confocal microscopy (Figure 4f). To analyze the distribution of CP bodies, we segmented the nucleus, the chromocenters (CC), CP and H3K27me3 nuclear bodies, as surfaces of adaptive size (Figure 4g-h). Volume measurements show that CP bodies are smaller than CC but larger than H3K27me3 bodies (Figure 4i, p < 0.001, Wilcoxon test) and are similarly distributed toward the periphery compared to CC and H3K27me3 bodies (Figure 4j). The image shows an intricate relationship between CP bodies, CC and H3K27me3 bodies. Measuring the overlapped volume ratio is a useful approach to quantify the fraction of spatially colocalizing bodies (Figure 4k), revealing in our case a frequent overlap of 50% or more of CP bodies with H3K27me3 bodies. Conversely, the overlap with CCs is less frequent and occurs to a lower extent (<20%). This image analysis workflow thus allows one to quantify features of the nuclear body distribution that are otherwise underappreciated with qualitative data alone. Based on this simple workflow, further processing steps can be implemented that would contribute to a refined analysis of the spatial pattern of CP proteins relative to chromatin density and H3K27me3 levels. This can include, for instance, the creation of intensity-based colocalization or ratio channels (not shown).

### Analysis of the higher-order chromatin organization in mitotic chromosomes

During mitosis, chromosomes reassemble into compact bodies resulting from increased chromatin fiber looping within the chromatids [47]. How sister chromatids resolve into distinct structures and which topological rearrangement contributes to the final organization start being understood. Yet, questions remain concerning the molecular mechanisms and the regulation of this dynamic process [47]. Also, whether the topological arrangement in mitotic chromosomes is conserved during evolution is not well known and is motivating for comparative investigations in less-well-studied models [48].

Oligo-FISH combined with spatial super-resolution structured illumination microscopy (3D-SIM) is a useful approach for resolving helical versus non-helical arrangement of chromatin fibers in chromatids. For instance, this method allowed us to confirm that the chromatids of barley metaphase chromosomes are formed by a helically wound ~400 nm chromatin fiber, the so-called chromonema [49]. Additionally, by measuring the volume of oligo-FISH painted regions and based on the DNA quantity used for the probes, it was possible to calculate the chromatin compaction. With this approach, different chromatin densities were found along the barley chromosome arm 5 HL. Interstitial arm regions were ~1.7 times more compact than regions adjacent to the subtelomeres (34.1 vs. 19.5 Mb/µm^3^, respectively) [49].

**Figure 5.**
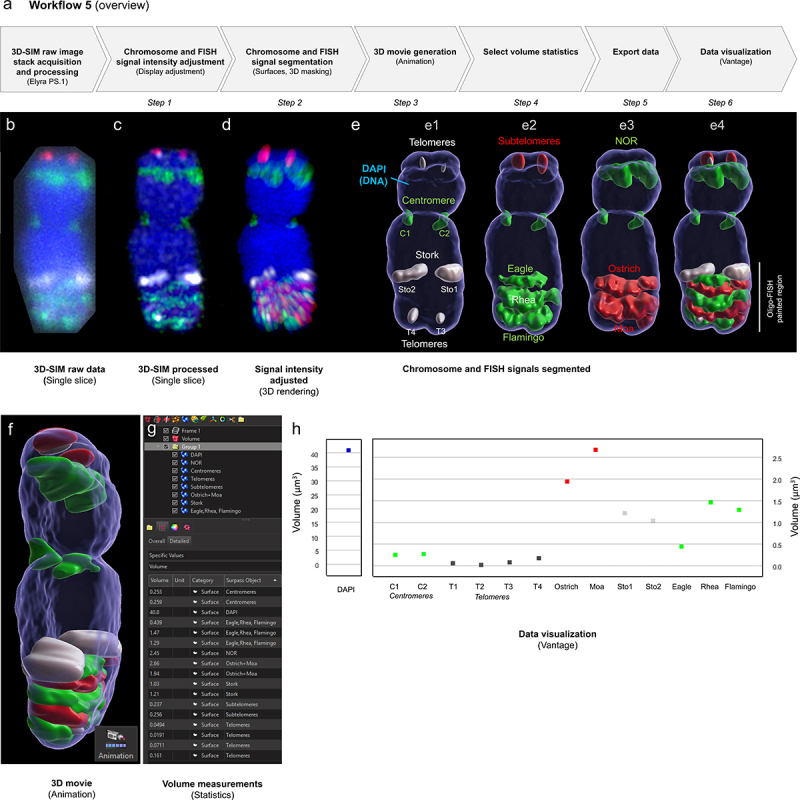
**Analysis of the metaphase chromosome ultrastructure using volume measurement of oligo-FISH labeled regions**. (a) Overview of the image analysis workflow. (b) 3D-SIM raw image slice from a stack containing 30 slices at widefield resolution. (c) 3D-SIM processed image slice showing increased super-resolution. (d) Display adjustment to optimize the visualization of signals with varying intensities. (e) Segmentation results: the chromosome is segmented using the DAPI channel and the generated 3D surface is used as a mask to specifically retain chromosomal FISH signals and exclude the background. The segmentation is presented sequentially for different FISH probe groups (e1-e3), and the result is shown in the merge (e4). e1, telomere, centromere, and Stork probes; e2, Subtelomeres, Eagle, Rhea and Flamingo probes; e3, 45SrDNA (Nucleolus Organizing Region, NOR), Ostrich and Moa probes. The Oligo-FISH probes label the bottom part of chromosome 5HL. (f) Side view of a 3D movie generated via the ‘Animation‘ tool (Supplemental File 5 – video 1). (g) Volume data are read in the ‘Statistics’Tab for selected surfaces. (h) Data visualization using the ‘Vantage‘ tool for individual objects (top).

Workflow 5 describes the processing procedure to segment an individual 5 H chromosome and the different FISH signals to obtain quantitative measurements on the degree of chromatin compaction (Figure 5a; Supplemental File 5). In this example, centromeres, 45SrDNA (NOR, nucleolus organizing regions) telomeres, and subtelomeres of somatic metaphase chromosomes were labeled with specific FISH probes as described [49]. In addition, half- and full helical turns of the chromonema were painted by oligo-FISH at the long arm of chromosome 5H (Figure 5, probes were named according to birds: Stork, Eagle, Ostrich, Rhea, Moa, Flamingo) [49]. 3D-SIM raw data image stacks (Figure 5b) were acquired using an Elyra PS.1 microscope system equipped with a 63×/1.4 Oil Plan-Apochromat objective, processed with the software ZENBlack (Carl Zeiss GmbH) (Figure 5c) [50] and converted into an Imaris file. DAPI and FISH signal intensities were adjusted for improving the visualization using the ‘Display Adjustment’ tool (Figure 5d). The DAPI-labeled chromosome was segmented using the surface tool, and the surface was used to mask the image to remove the background signal outside this region of interest. Additional surfaces of the other, differently colored FISH signals were generated (Figure 5e-f, Supplemental File 5 – Video 1). The surface volume data were established (Figure 5g), exported for further analysis by compiling several images, and used to calculate the volumetric density of the different FISH-labeled regions along the chromosome [49]. An example plot for one chromosome is shown in Figure 5h using Vantage.

### Analysis of centromere and telomere positioning

**Figure 6.**
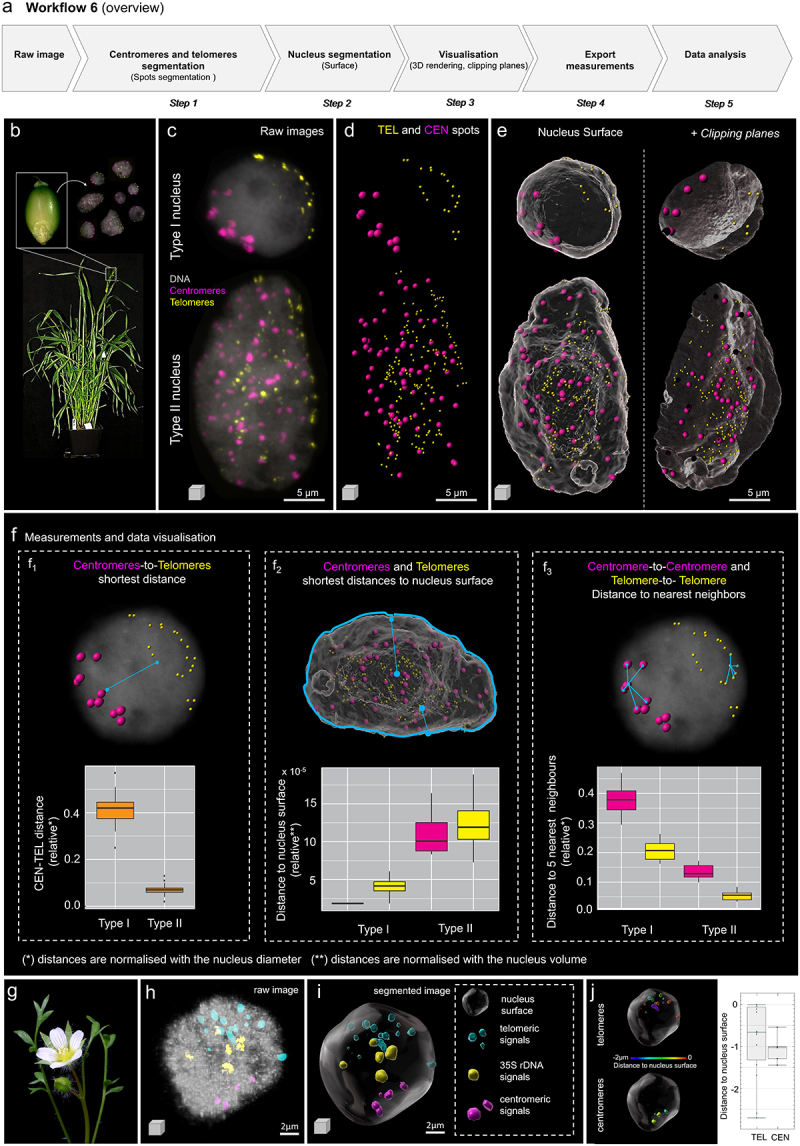
Analysis of centromere and telomere positioning in the interphase nucleus (continued). (a) Workflow overview showing the main steps to process the 3D image and identify centromeres and telomeres and their position in an interphase nucleus. The workflow is illustrated with seed nuclei from barley (a-f) and leaf nuclei from Limnanthes (g-i). (b) Barley plant, seeds and isolated nuclei stained by FISH for centromere and telomeric repeats (see main text for details). (c) Raw images (3D projections) of type I and type II nuclei showing centromeric (magenta) and telomeric (yellow) FISH probes signals, counterstained for DNA (DAPI, gray). (d) Telomeric (TEL) and centromeric (CEN) signals were segmented as spots. (e) 3D rendering together with nucleus surfaces (gray) following segmentation, whole nuclei (left) or clipped (right), exposing the CEN and TEL signals in the interior of the nucleus. (f) The distribution of telomeres and centromeres is described according to three measurements derived from spot-to-spot or spot-to-surface statistics: shortest distance between centromeres and telomeres (f1), shortest distance of centromeres to the nucleus surface and shortest distance of telomeres to the nucleus surface (f2), inter-centromere and inter-telomere distances computed as the average distance to the nearest 5 neighbor spots of the same category (f3). In blue, schematic representation of the measured distance. Distances were exported and normalized to the nucleus diameter (f1, f3) or nucleus volume (f2) and plotted using the ggplot GUI online tool (https://shiny.gmw.rug.nl/ggplotgui/). The lower and upper hinges of the boxplots correspond to the first and third quartiles of the data, respectively, the black lines within the boxes mark the median. Five to ten nuclei were used for each measurement. Black spots beyond the whiskers represent outliers. (g-j) Illustration of the workflow on a Limnanthes leaf nucleus, (g) Limnanthes floccosa subsp. bellingeriana, (h) Raw image (3D projection) of a nucleus stained for centromeric repeats (magenta), telomeric repeats (cyan) and rDNA repeats (yellow) by FISH, counterstained for DNA (DAPI, gray), imaged by confocal laser scanning microscopy, (i) 3D nucleus following segmentation of FISH signals and DNA as surfaces. (j) Distance of the different segmented groups relative to the nucleus surface were plotted in Imaris Vantage; images showing a distance-coded coloring are shown for centromeres (CEN) and telomeres (TEL).

Arabidopsis and barley are eukaryotic models contrasting in their 3D interphase chromosome organization. Arabidopsis has a small genome of about 157 Mbp per haploid DNA content (1C) packed into 5 chromosomes (2 n = 10), whereas the barley genome is large, with around 5.1 Gbp/1C divided into 7 chromosomes (2 n = 14) [51,52]. In Arabidopsis, centromeres are distributed relatively equally around the nuclear periphery to which they are attached, while telomeres are associated with nucleoli and each chromosome occupies a discrete territory within the nuclear space [53]. In barley, interphase chromosomes are organized in the so-called Rabl configuration with the centromeres and telomeres clustered at opposite nuclear poles [52]. While the Rabl configuration has long been thought to be prevalent among monocot species, recent studies show that it also occurs in dicot species and that variations exist within the same phylogenetic group [54]. In addition, this peculiar organization can occur in a tissue-specific manner, as in rice [55]. To better characterize the occurrence of Rabl vs. non-Rabl configurations and their possible intermediates, in different species and tissue types, there is a need to define an image analysis workflow quantifying telomere and centromere distribution in the nuclear space. We present such a workflow (overview Figure 6a) illustrated with two examples, corresponding to studies of chromosome organization in a monocot species (barley, Figure 6b-f) and in a dicot species (*Limnanthes floccosa* subsp. *bellingeriana*, Figure 6g-j). Details, parameters, and demo images are available in Supplemental Files 6.

A first example is given for barley nuclei (Figure 6b-f). Nuclei extracted from seeds (Figure 6b) were flow-sorted as described [56] and labeled by FISH using fluorescently labeled oligoprobes (Cy3-labeled *CEREBA*-centromeric repeat; [57] and Cy5-labeled Arabidopsis-type telomeric repeats [58]). Z-stack images were acquired with an epifluorescence microscope connected with a spinning disk (Andor, Oxford Instruments, UK). Centromeric and telomeric FISH signals and the DNA counterstain (DAPI) were pseudo-colored in magenta, yellow, and gray, respectively. Two types of seed nuclei are shown (Type I, Type II, Figure 6c). Images are first segmented on the channels reporting on FISH signals to create spot objects corresponding to centromeres (CEN) and telomeres (TEL), (Figure 6d) using the automated tool. The spot diameter is adjusted to the average size of FISH foci (ca 300 nm). The nuclear surface is rendered using a low smoothing factor (Figure 6e). For a better visualization of the spot distribution inside the nucleus, the surface is set to transparent or can be digitally sectioned using a clipping plane (Figure 6e). This segmentation and 3D visualization approach allowed us to realize that the seed nuclei population was composed of two categories of nuclei. In type I nuclei, centromeric and telomeric spots are grouped to opposite sides of the nucleus, reflecting Rabl-like features. In type II nuclei, they distribute in the whole 3D nuclear space, which corresponds to a non-Rabl configuration. To support this observation by quantitative measurements, we exported three types of distance measurements (Figure 6f): (i) shortest distance between centromeric and telomeric spots (Figure 6f_1_), (ii) shortest distance of centromeres and telomeres to the surface, corresponding to the nucleus border (Figure 6f_2_), and (iii) average distance to top five neighboring spots for each group (centromeres, telomeres, Figure 6f_3_). Because distances depend on the nucleus size, we normalized them using the nucleus diameter (graphs shown in Figure 6f express relative distances). The quantitative analysis shown in Figure 6f based on ca. 20 nuclei supported a contrasted spatial distribution of telomeres and centromeres in the two categories, with notably clear segregation of telomere and centromere groups in Type I nuclei (f1), located closely to the nuclear surface (f2). We also noticed a shorter distance between telomeres and centromeres in Type II nuclei, which was unexpected. Because this type of nuclei is frequently highly endoreduplicated, this led us to investigate further the relationship between ploidy and chromosomal organization (Nowicka, Pecinka et al., submitted).

A second example is shown using nuclei from *L. floccosa* subsp. *bellingeriana* (Figure 6g-j). Nuclei extraction from different types of tissue, FISH protocol and imaging were previously described by [54,59–61]. A similar procedure was applied to segment the nucleus based on DAPI staining (gray) and FISH signal reporting on the telomeres (cyan), centromeres (magenta) and 35S rDNA loci (yellow) (Figure 6h-i). In this example, centromeres and telomeres clearly showed clustering toward the nuclear periphery as shown with a median distance of spots around 0.6 µm (TEL) to 1 µm (CEN) in a nucleus of *ca*. 12 µm diameter (Figure 6j). We used this workflow for the analysis of nuclear organization in Crucifer genomes [54], in seven diploid species with up to 26-fold variation in genome size. This allowed to unveil species-specific patterns in nuclear organization [54].

For a trained user, the workflow takes approximately 20 minutes or less per image. This workflow can be used to compare the spatial distribution of chromosomes at interphase using centromeres and telomeres as references. Distance measurements across image replicates offer the possibility to detect quantitative differences invisible to the eye, between tissue types and cell types and to characterize potential mutant phenotypes in genetic analyses.

### Analysis of mitotic chromosome orientation during division

Mitosis is the process by which organisms increase the number of cells. In plants, the highest number of mitotically active cells can be found in the root and shoot apical meristems (RAM and SAM, respectively) [62]. We focused the analysis on chromosome organization and orientation in living barley roots. Both cell division orientation and cell elongation contribute to the oriented growth of the root. Changes in the mitotic division orientation affect root shape and anatomy [63]. In *Vicia faba*, chromosome positioning correlates with the cell division plane and ultimately cell shape [64]. Notably, it was speculated that cell size could be a limiting factor forcing the spindle axis to be tilted, deviating slightly from the main axis of cell and organ elongation. Analyzing the orientation of mitotic chromosomes during cell division is thus relevant to understand this intricate relationship.

**Figure 7.**
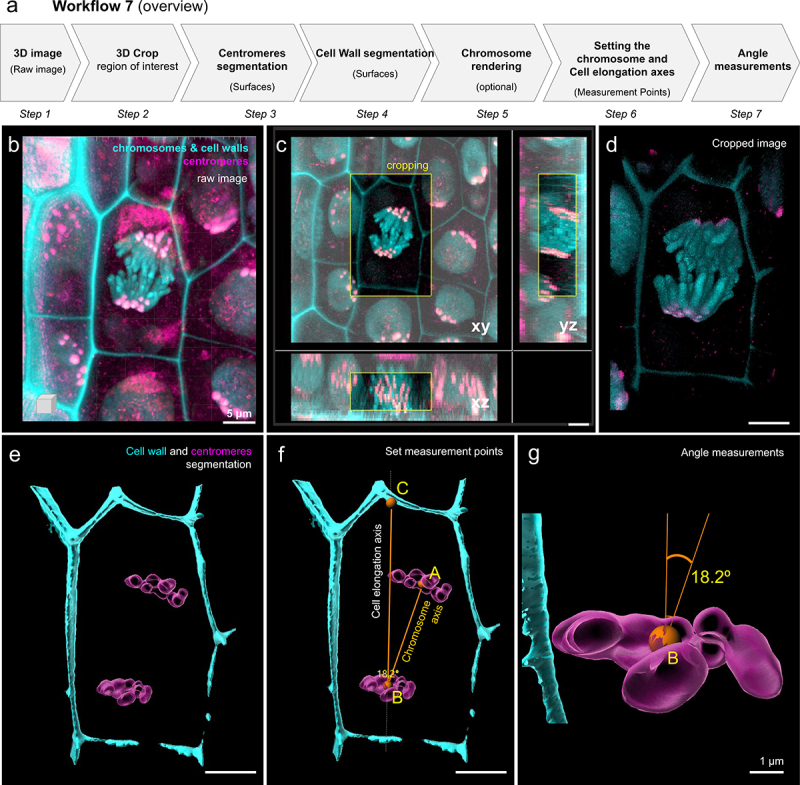
**Division angle measurement using surface-rendered cellular structures in living barley root cells. (next page)**. (a) Workflow overview showing the sequence of tasks to process a raw picture up to the setting of measurement lines within the 3D cell space. (b) Confocal imaging of barley root tissue from a young seedling expressing CFP-H2B marking the chromosomes (cyan) and RFP-CenH3 marking the centromeres (magenta). In addition, cell wall autofluorescence upon UV excitation was used to mark the cell’s boundaries (cyan). The image is a partial projection from a z-stack. (c) 3D cropping of the image to select a region of interest containing a dividing cell in mitotic anaphase (yellow frame). Orthogonal projections are shown in xy, yz and xz. (d) The cropped image is rendered in 3D using the ‘blend’ mode. (e) **‘**Surface’ rendering of segmented centromeres (magenta) and the cell wall (cyan). (f) Setting of ‘Measurement points’ and their connective lines. AB defines the axis along which chromosomes are pulled (orthogonal to the chromosome plates), BC defines the cell’s elongation axis. (g) Detailed visualization of the lower metaphase plate and angle formed between both axes defined by AB and BC measurement lines. The angle is measured in 3D by Imaris. Scale bars: b-f, 5 μm; g, 1 μm.

We designed a 3D microscopic image analysis workflow described in Figure 7a and detailed in Supplemental Files 7 containing a protocol and troubleshooting tips. We used barley chromatin and centromere fluorescent marker lines (FMLs) expressing translational fusions of histone H2B with CYAN FLUORESCENT PROTEIN (CFP-H2B) and -CENTROMERIC HISTONE H3 with RED FLUORESCENT PROTEIN (RFP-CENH3), respectively (Kaduchová, Pecinka et al., in preparation). Z-stack images were acquired using a Leica TCS SP8 STED3X confocal microscope equipped with a Leica Application Suite X (LAS-X) software version 3.5.5 with a Leica Lightning module (Leica, Buffalo Grove, IL, USA) (Leica Microsystems, Wetzlar, Germany). In addition, we took advantage of the fact that barley cell walls have an autofluorescence detectable in CFP emission spectra [65], allowing simultaneous visualization of chromosomes, centromeres, and cell walls. Centromere signals were pseudo-colored in magenta, chromatin with cell walls in cyan. The raw image (Figure 7b) presenting several cells in the root was cropped around one cell showing chromosomes at anaphase (Figure 7c). Centromeres were segmented with the ‘spots’ tool (Figure 7d). Chromosomes and cell walls were segmented using the ‘surface’ tool (Figure 7e). Using the tool ‘Measurement point’, we created spots (connected by a measurement line) at key positions, providing information on the cell elongation axis (A-B), on the pulling axis of the chromosomes (A’-B) and a reference axis (B-C) (Figure 7f, see Supplemental File 7 for detailed explanation), which allowed for angle measurements (Figure 7g).

For a trained user, the workflow takes approximately 30 min per image. This workflow will allow one to measure the relationship between the orientation of the spindle axis during division and cell shape (elongation) and its variation between tissue and cell types. In addition, the possibility to measure this relationship opens the possibility to quantify the effect of genetic or environmental factors with large or subtle effects on the cell division axis.

### Conclusive remarks

We present here a set of seven image analysis workflows enabling the quantitative study of the spatial organization of chromosomes and chromatin components. The workflows cover applications for studies at interphase (workflows 1, 2, 4, 6), mitosis (workflow 5, 7) or meiosis (workflow 3). Workflows 1, 2 and 4 demonstrated the possibility to discover spatial distribution patterns, taking as examples transcription clusters, nuclear bodies and speckles and nuclear envelope-associated proteins. Such patterns were revealed thanks to the exploration of possible relationships between distance and intensity measurements among the different objects of the segmented images. Workflow 6, exploring genome organization at interphase, illustrates the quantitative power of image segmentation to precisely measure the spatial positioning in the nuclear space and the clustering of telomeres and centromeres. These features describe different types of 3D genome organization depending on cell type and species. The interest in performing image analysis for chromosome studies was further illustrated with workflows 3 and 5 focusing on condensed chromosomes at meiosis or mitosis, respectively. Workflow 1 demonstrates the usefulness of image segmentation for quantifying the number and distribution of crossover components on meiotic chromosomes and revealing the possible enrichment in relation to the synaptonemal complex and that of chromosomal regions. Workflow 5 shows that volumetric measurements of FISH signals enable determining chromatin density (compaction) in different genomic regions. Finally, workflow 7 proposes an approach to measure the angles between chromosome and cell elongation axes and to investigate the relationship between cell division orientation and chromosomal positioning.

Image segmentation delivers a wealth of information related to signal intensity, distribution pattern (texture), shape, size and distance relationships between segmented objects [1]. Thus, images become associated with many variables and entry types, generating big data. Those can either be explored in a non-hypothesis-driven way using multidimensional data analysis (Bagheri et al., 2022) or in a hypothesis-driven manner following a careful choice of data for export. Even when exporting a selective number of image descriptors, the analysis of replicate image datasets, in different conditions (treatments or genotypes), labeled for multiple components, quickly generates a large numerical dataset. Versatile data visualization interfaces become handy at this stage. Here, we provided some examples among the numerous available solutions. We developed a customized Shiny-based (shiny.rstudio.com/) data visualization interface, DataViz, for processing (normalizing, filtering), exploring and plotting intensity, morphology and distance measurement data exported from segmented images. Normali-zation of intensity or distance measurements per image is important for considering variations that may arise between images during sample preparation, imaging or image acquisition [1,12]. The examples provided here propose different strategies depending on the image analysis question. Versatile data visualization greatly facilitates the explorative work, which in turn has the potential to seed discoveries, revealing unexpected patterns or relationships and driving further analyses or experiments.

Although these workflows were developed to analyze nuclei and chromosome organization mostly in plant cells, these are conceptually applicable to nuclei of other species. An example is shown in workflow 4 with the analysis of nuclear bodies in mouse embryonic stem cells. In addition, these image analysis workflows are expected to inspire cell biologists beyond the study of the nucleus and its constituents. For instance, transposed at the cellular scale, workflow 1 or 4 could be applied to analyze the spatial distribution of vesicles or cytoplasmic bodies within a cell, using cell segmentation modules to create the initial surface object (see, for instance, but not exhaustive, references [66–68]).

Finally, while based on a particular (commercial) software piece, the concept of these workflows is expected to be transferable to other concurrent software offering similar image analysis tools (Supplementary File 8 – Table 1). One example is the 3D ImageJ Suite [8,10] popularized by the NEUBIAS COST action [8] which also offers a set of Fiji-based plugins for analyzing the spatial distribution of nuclear signals.

The increasing number of user-friendly platforms and the growing performance of segmentation algorithms greatly facilitate image analysis. Yet, this progress should not elude the need to reflect on the pertinence of the segmentation applied relative to the image features extracted by the process – and that will ultimately be interpreted in a biological context. Segmentation is influenced by the image quality, and specific metrics have been proposed to control for it [69]. In addition, when establishing a segmentation pipeline for the first time, several thresholds relative, for instance, to signal intensity, contrast and seed size must be adjusted that influence object detection. These thresholds influence the results in terms of the number, size, shape and texture of objects (discussed in [1,3]). In a semi-automated, user-guided segmentation such as proposed here, how to decide on a specific threshold or cutoff values can be difficult (of note, this type of decision is similar to those met in bioinformatics analyses to select sequencing reads based on their quality, replication and cutoff levels). Threshold values must be justified with sufficient criteria to be reproduced and understood by peer users. Alternatively, and when image quality is relatively homogeneous in a dataset, it is possible to use values automatically proposed by the algorithm as those are usually derived from image-based statistical parameters. We address this issue and propose solutions for each workflow in their detailed description (supplemental files 1–7). Yet, the rapid emergence of machine-learning (ML) based segmentation algorithms is expected to ease the application of optimal segmentation parameters, although an initial investment is required to train the algorithm with ground-truth images (discussed in [1,12]). Eventually, and perhaps most importantly, the image analysis becomes only relevant when two or more biological conditions are compared. Sample preparation and image analysis done in the same conditions and by the same user, ideally in a blind analysis design, will average possible technical and cognitive biases throughout the datasets. This will, in turn, allow us to draw relevant conclusions relative to the type and the order of magnitude of changes correlated with a treatment, a genotype or cell type, for a given spatial pattern describing nuclear, chromatin or chromosome organization.

The compendium of workflows presented here, with its illustrations, training images and detailed guidelines, aims at inspiring experimentalists in the field of chromatin, chromosome and nucleus organization studies, with no or little expertise in image processing. This effort responds to the rapid development of microscopy imaging techniques and the needs of a wider community to have well documented and conceptually accessible image analysis tools [13]. Ultimately, this allows to exploit image data to an unprecedented level of analysis.

## Supplementary Material

Supplemental Material

## Note

1. https://omero.bio.fsu.edu/webclient/userdata/?experimenter=-1 folder IDP 3008_Randall-Baroux2022
