# Supplementary material for: Image analysis workflows to reveal the spatial organization of cell nuclei and chromosomes
Source: Nucleus. 2022 Nov 29;13(1):277–99. doi: 10.1080/19491034.2022.2144013 (PMC9754023; doi:10.1080/19491034.2022.2144013)
Supplement: Supplemental Material [file KNCL_A_2144013_SM9221.zip › Supplemental File 2 Text and Table/Supplemental File 2 - Text_and_Table/Workflow 2-Text.docx]

# **Workflow 2 – Analysis of the spatial distribution of proteins located at the nuclear periphery**

This step-by-step image analysis workflow can be practiced with the training image, supplemental file 2- image 2. The image is that of an Arabidopsis nucleus stained for a nuclear envelope (NE)-associated protein tagged with GFP (NE-GFP). In this example, the NE-GFP form spots of variable sizes at the NE and display an asymmetry distribution with an enrichment at the equatorial plane of the nucleus. Images were acquired using seedling expressing the NE-GFP marker at 7 -14 days after germination and roots were imaged in 3D using a confocal microscope equipped with an Airyscan module (Huff, 2015). The asymmetric distribution was confirmed by changing the initial grey level rendering of pixel intensities for the “Fire-scale” coloring mode indicating higher intensities at the equatorial plane (middle) than at the poles (top and bottom). The following procedure aims at characterizing this asymmetric distribution.

The key steps and parameters are also summarized in the supplemental file 2- Table 2. When applied to other, similar images, these parameters must be adjusted as they highly depend on image resolution and quality (signal-to-noise ratio).

***Step 1- segmentation of the NE-RFP peripheral domain.***

The domain expressing NE-GFP is segmented using the ‘*Surface*’ function of Imaris divided in four sub-steps guided by the built-in creation wizard. At the first step (**1/4**) the default option to go on with an automatic segmentation is kept. The option ’*segment only a ROI*’ is not selected. Also, the option ‘*classify surfaces*’ is not necessary here and is unselected but ‘*object-object statistics*’ is kept as it allows for distance calculations. At the next step (**2/4**) the appropriate source channel is selected (GFP channel in our case). The smoothing factor is kept to the automatic surface grain size (calculated by the software based on image properties) here = 0.2 µm. The thresholding is set for background subtraction with ‘*diameter of largest sphere*’ = 0.3µm, corresponding roughly to the width of the NE-FP domain that can be measured at the nuclear periphery using the slice viewer mode.


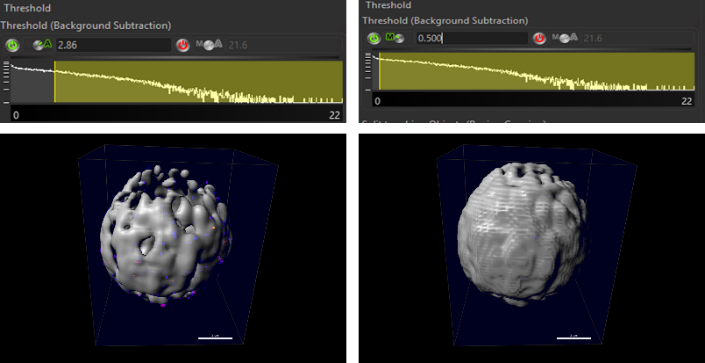
The segmentation threshold is set up at the next step (**3/4**): it is lowered as much as possible to capture more permissively the signal at the NE including regions of low intensities. This threshold must be adjusted on each image depending on signal intensity and of the background to capture as best as possible the signal at the nucleus periphery (from our dataset, this value ranges between 0.5-10). An example of the influence of the threshold is given in **Figure 1.**

Figure 1- Influence of the intensity threshold (histogram, upper panel) on the segmentation result (lower panel)


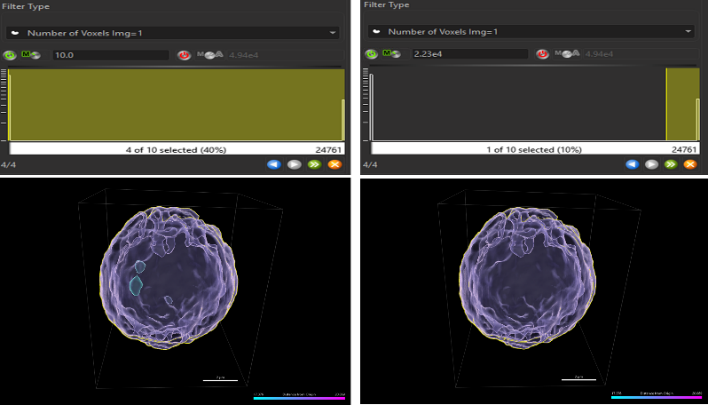
This permissive threshold has however the drawback to create additional and unwanted, small surfaces capturing lower signal intensities (Figure 2). These additional surfaces are visible at the next step, which is a ‘*filter step*’ (**4/4**). The histogram shows small surfaces with a small number of voxels on the left side of the histogram and a category on the far right with a high number of voxels corresponding to our surface-of-interest. The selection window (yellow) is adjusted to capture only this large surface (image, right panel). Finally, segmentation is applied (green arrow in the creation wizard) and the surface is created. Note that it is a hollow surface.

Figure 2- Removal of unwanted surfaces at the filter step. Left: the permissive intensity threshold captures the signal all around the nucleus but also smaller regions of low intensity inside the nucleus (colored objects). Right: at the filtering step, only the largest surface is kept (selected)

Note: Thresholding is an important step. In some images, a permissive threshold may create a surface with invaginations towards the center of the nucleus, due to residual signal of low intensity. These invaginations could lead, later on (Step 5), to the segmentation of NE-GFP clusters which are not located at the periphery. Those can be manually filtered by distance (explained later). On different images, however, invaginations may be more invasive. In that case, the creation threshold may be adjusted as explain above. Alternatively, a different approach (not shown here) consists in creating first a cleaning mask: create a surface using manual contours capturing the inner space of the nucleus; use it as a mask by setting voxels inside the surface to zero. The resulting channel will be devoid of signal in the inner space of the nucleus and can be used for surface creation as described in this Step 1.

***Step 2- Create a positional reference at the center of the nucleus.*** To classify the spots later according to their position at the nuclear surface, a reference is needed. This is achieved in two sub-steps. First, a spot object is created at the ‘*center of mass’* (CoM) of the surface object (good proxy for the center of mass of the nucleus itself). Second a reference frame is position at the Center of mass.

**Step 2-1 Create a spot at the Center of mass:** Two approaches can be used to determine the *center of mass* (i) either manually using the CoM coordinates, (ii) or automatically using an Imaris plugin requiring a connection to Matlab. This results in a spot located at the center of mass of the nucleus (Figure 3)


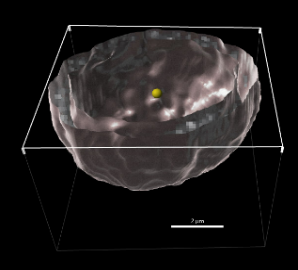

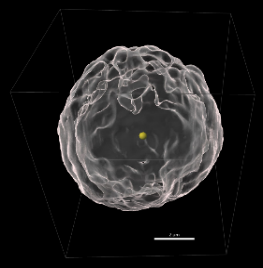


Figure 3- A spot is created at the center of mass of the Surface (yellow dot in the image)

For (i), in the ‘*statistics*’ Tab of the Surface created at step 1, select ‘*detailed /specific values’* and ‘*center of homogeneous Mass*’. This statistic parameter must be activated in the ‘*Edit*/*Preference/statistics/Surface/Surface’* main menu of Imaris (9 and later). Write down the X, Y and Z values of the CoM. In a next step, create a ‘*Spot’* object, skip the automatic segmentation and add manually a spot by selecting ‘*shift + left click*’ (a new spot appears at the upper left corner of the frame). Chose the spot radius (for instance, 0.2µm) and position the new spot at the center of the nucleus by entering the X, Y and Z coordinates of the CoM.

For (ii), in the NE-GFP Surface object, select the *’Tools’* Tab (far right) and select the plugin ‘*Center of Mass To Spot’*. For this, Matlab and the corresponding Matlab plugins from Imaris should be installed (see Bitplane help or your facility manager). It takes up to 20sec for Matlab to start, thus click only once and wait. A Matlab pop-up window asks to enter the radius of the spot. Enter 1. This creates a large spot object. You can change the size in the Settings tab of the newly created spot called ‘[name of your Surface] Center Of Mass’.

**Step2-2 Reference frame at the center of Mass:** The second sub-step is to use the CoM spot to dock a new cartesian reference origin in the image. For that purpose, create a ‘*Reference Frame’* object. It appears in the lower left corner of the 3D frame (**Figure 4, left**). Drag it manually to overlap with the spot ‘center of mass ‘ (remove the surface display for easier manipulation) (**Figure 4 right**). Rotate in all directions to make sure the overlap is correct. It is important to align the Z axis with the longest axis of the nucleus as they are usually not perfectly spherical. *Note. This docking is manual in Imaris version 9.8. Mind a possible, automatic docking option in later versions.*

Figure 4- Dock a new Reference Frame at the center of mass

***Step 3- 3D masking using the NE-RFP surface.***

Because we focus our study to NE-GFP signal located at the nuclear envelope, the Surface is used as mask to create a new channel capturing the peripheral signal only (inside the surface). For this, select the Surface object and use the ‘Mask all’ option in the Edit tab, duplicate the channel and set voxels outside surface to zero. The result is a new channel called “GFP channel-Masked”.

***Step 4- NE-RFP cluster segmentation.***

The NE-GFP signal shows interspersed, high-intensity clusters alternating with a continuous, low intensity signal. Here, we aim at segmenting the clusters enriched in NE-GFP. A first inspection using the ‘ortho slicer’ tool showed that the GFP signal form clusters of varying sizes and intensities . To capture this variability, we will segment the signal as spots or adaptive sizes, in six sub-steps. First, create a *‘Spot’* object. At the first step **1/6**, select the ‘*region growing*’ option that will create spots of adaptive size. At step **2/6**, select the GFP channel-Masked and choose as a starting spot size the value of 0.3µm (this size is estimated using the slice viewing mode). Select the ‘*background subtraction’* mode. At the next step (**3/6**), adjust the intensity threshold toa permissive range: deactivate the lowest threshold (enter the value zero or press the ‘off’ icon near the lowest threshold). At step **4/6**, choose the method ‘*absolute intensity*‘, which is appropriate for this high resolution image (*note: on an image with less resolution or lower signal to noise ratio, the method ‘local contrast’ might be better*). Step **5/6** consists in choosing the region within which the spots will grow, the default value, proposed by Imaris based on the image and signal properties, is kept. At that stage you can directly complete the creation process and skip the classification step (which will be done and edited also after spot creation) to explore your data before creating spot categories.

***Step 5 -Classification***

A first inspection of the image gave insights into the possible enrichment of NE-GFP signal at the equatorial plane of the nucleus. To verify this hypothesis, categories of spots will be created based on their axial position. For this purpose, the *Reference frame* defined at the center of mass (step2-2) of the nucleus will be used as a reference.

**Step 5-1 *Create classes***: This can be done during spot creation, at the classification step **6/6** or after spot creation using the spot object *Classification* tab. Select the variable ‘*position z Reference frame*’ to create three classes according to their spatial position (by default Imaris proposes only two classes, add one using the ‘+’ button) In this example, we take a median class consisting in spots distributed within a 4µm slice centered on the center of mass. Hence on the histogram, use -2um and +2um as the lower and upper limits of the intermediate class (magenta, **Figure 5 left**). The A, B and C classes can be renamed in top, middle and bottom by clicking on the colored squares. Then complete the process (green arrow) leading to the classification of the spots in three colors (**Figure 5 right**).

Figure 5. NE-GFP spot classification according to their position along the Z axis, in the new reference frame (origin at the center of mass).


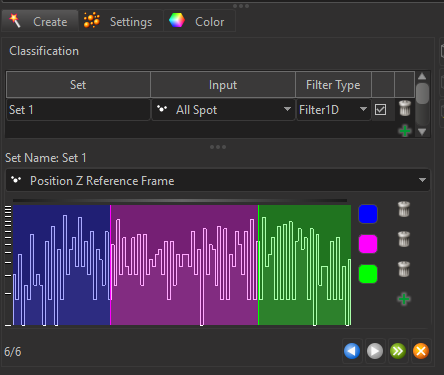

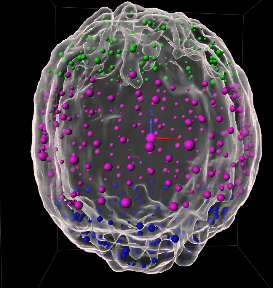

***Step 5-2- Removing outliers:*** Depending on signal distribution, the permissive surface created at Step 1 can generate invaginations inside the nucleus capturing signal of middle/low intensities. Consequently, spots are also created in these regions. Since we want to focus on the peripherally located clusters, we consider these spots as outliers. To remove them, select the spot object and use the ‘*Filter’* tab to filter the spots using the variable ‘*average distance to 9 nearest neighbors*’ as those spots are expected to locate further away. The image below shows three outliers (yellow), corresponding to the yellow spots with inner position on the nucleus (**Figure 6 left**).

Figure 6- Select outlier spots using the Filter Tab and the variable ‘average distance to 9 neares neighbors’ (left). Selected spots are in yellow (right) and can be deleted in the ‘Edit’ Tab.

In this example, the surface was set to transparent (grey) and the spots were displayed as ‘*Center point*’, size 15 (*Spot* object/*Settings* Tab), their color was set to purple (*Spot* object/ *Display* Tab, unselect ‘*labels override base color’*), such that the selected spots are highlighted in yellow and are better visible (**Figure 6 right**). The selected spots (yellow) are then deleted in the *Spot*/*Edit* tab. The diagram stretches and a few more outliers are visible, repeat the operation. Depending on images, repeat the correction a few times, possibly also with the average distance to 5 neighbors, always verify their location by rotating the image in 3D.

Note that if the invaginations at the surface are too abundant following segmentation at Step1, this manual curation step can become cumbersome. Then an alternative approach for surface creation must be undertaken – See Note at the end of Step 1

**Step 5-3. Filtering spots for biologically relevant size**

The ‘Growing spot’ function of Imaris creates spots of unlimited size if this is not instructed during the spot creation. Spots of biologically relevant size can be filtered during the creation process or after creation, using the *Filter* Tab in the *Spot* object. Here we used the variable ‘Volume’ to select spots between 0.0001-0.1µm^3^ corresponding to NE-GFP clusters with a diameter of ca 120-600nm.

**Step 5-4. Estimating the proportion of signal captured by spots:** The spots capture clusters of varying size and leave unsegmented some interspersed signal. To estimate the ‘capture efficiency’ of the spot segmentation, create transiently a masked channel (Spot/Edit/Mask all, duplicate channel, set voxels outside spots to zero), and read the intensity sum of the original channel in the Surface (ring used to mask the original image) and compare it with the intensity sum of the last created channel (masked on spots). Make the ratio.

***Step 6 – Data selection and export***

For analysis in a third-party software, select the statistics to export in the general Imaris menu *Edit* / *Preferences*/*Statistics*/*Spots*. Depending on Imaris versions, the statistics are classified in sub-folders. Inspect all of them to find your statistics of interest. In this study, statistics of interest are: volume, intensity sum, intensity mean, intensity standard deviation and possibly diameter and position (x,y,z)

Apply the selection then select the *Spot* objects, go to the statistics tab and click on the ‘Export All’ button (multiple disks icon, bottom right).

Rename your Surface and Spot objects (optional). Make sure each object has the same name in each image (mind case and space), and that the image has a short name, to ensure an error-free reading of the collective data exported from several images, for compiling a unique dataframe used for plotting.

***Step 7- Data visualization***

Following image segmentation, the image can be described by a series of graphics plotting the distribution of various statistics-of-interest per object using the ImarisVantage module. Imaris Vantage can also plot the statistics of several images providing they carry objects (spots, surface) with the exact same name. In Arena, select all segmented images (ctrl shift) and select ‘add plot’ (top tabs). Note that to compare intensities, or use intensities from several images, those need to be normalized for each image. Normalization has then to be done in a third-party software (eg DataViz, see workflow 1).

**References**: Huff, J. (2015). The Airyscan detector from ZEISS: confocal imaging with improved signal-to-noise ratio and super-resolution. *Nature Methods, 12*(12), i-ii. doi:10.1038/nmeth.f.388
