# Supplementary material for: Image analysis workflows to reveal the spatial organization of cell nuclei and chromosomes
Source: Nucleus. 2022 Nov 29;13(1):277–99. doi: 10.1080/19491034.2022.2144013 (PMC9754023; doi:10.1080/19491034.2022.2144013)
Supplement: Supplemental Material [file KNCL_A_2144013_SM9221.zip › Supplemental File 3 Text and Table/Supplemental File 3 - Text_and_Table/Workflow 3-Text.docx]

# **Workflow 3 - Analysing cross-over distribution in meiocytes**

The supplemental material provides two demo images (supplemental file 3- images 3a and 3b):

- image 3a (I.Colas): barley meiocyte at zygotene stage was immunostained for DMC1(Ch=2), ZYP1 (Ch=3) and ASY1 (Ch=4) and counterstained for DNA using DAPI (Ch=1). The image was acquired by confocal microscopy (ZEISS LSM 710) as described in (Colas et al, 2019).

- image 3b (M.Ayoub): barley meiocyte at late pachytene stage was immunostained for ASY1 (Ch=2), ZYP1 (Ch=3) and HEI10 (Ch=4) and counterstained for DNA using DAPI (Ch=1). The image was acquired by 3D-SIM (Elyra PS.1 microscope system and its software ZENblack (Carl Zeiss GmbH) (Hesse et al., 2019).

*The workflow is based on Imaris 9.5 or later. Some options (spot and surface classification and distance calculation) may not be available or presented differently in earlier versions. For additional classification possibilities as those presented here, see* [*https://imaris.oxinst.com/support/imaris-release-notes/9-6-0*](https://imaris.oxinst.com/support/imaris-release-notes/9-6-0)

The key steps and parameters are also summarized in the supplemental file 3- Table 3. When applied to other, similar images, these parameters must be adjusted as they highly depend on image resolution and quality (signal-to-noise ratio).

*Step1- Image pre-processing: deconvolution*

Image 3a was deconvoluted using IMARIS Clearview (9.5.1): in the image processing tab, choose deconvolution; we used the standard deconvolution parameter with an interactive algorithm (5- 10 iterations). Using more than 10 iteration is not recommended as it can introduce image artifacts.

**Note: that for colocalization studies, it is important to have corrected the image before processing, for chromatic aberrations that generate spatial shifts between the blue emitting and green/red/far red emitting labels . Channel shift correction can be done based on measurement on spectrabeads with the same imaging settings and entering the corresponding pixel values in Edit/channel shift. Default parameters, calculated by Imaris-based on image properties, are kept throughout the wizard and using signal detection based on ‘absolute intensity’. Depending on image quality, these parameters (smoothing factor, threshold) may have to be adjusted.*

*Step2- Segment the chromosomes and use the surface as mask (image 3a)*

The immunostained proteins of the synaptomal complex and cross-over machinery of image 3a distribute close to, or on the chromosomes. A fraction of the immunostaining signal, however, distributes broadly over the nuclear area. To create a clear image focusing on the chromosomal environment, we use the DAPI channel to create a surface, using the Surface tool. Here, we choose permissive parameters: for image 3a, the smoothing factor was increased to 0.2µm (~2x the proposed value) and no background subtraction was applied. The intensity threshold was lowered to 800. This value may differ between images and should be adjusted to capture the relevant information. A major reason for creating a permissive surface is to be able capturing also immunosignals not tightly colocalizing with DAPI. This is the case, in image3a, for ASY1. The effect of a tight surface on the chromosomes is to lose some ASY1 signal when applying a mask at latter stage (Figure below, note the loss of signal in the yellow inset for surface 1 and 2).


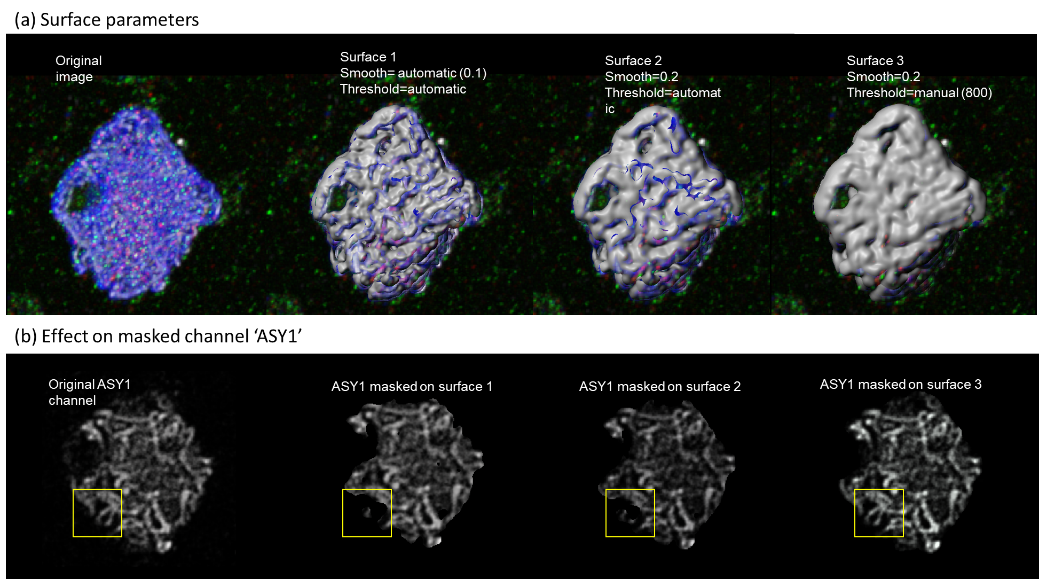


When creation is completed, the chromosomes’ surface is then used as mask to remove signal not colocalizing with the surface, in all other channels: in the edit tab, select ‘Mask all’, select Ch=1, keep the option ‘duplicate channel’ and ‘set voxels outside surface to zero’. Repeat this for all other channels. This creates four new (masked) channels focusing collectively on the biologically relevant information (e.g. How many foci are on ASY1 or ZYP1 axes?). In the ‘Display Adjustment’ Tab, deselect the original channel to visualize a clean image (Figure 3c).

For Image 3b, displaying a different meiotic stage, the analysis focused on HEI10 spots associated with ZYP1 hence the chromosomes were not segmented.

*Step3- Segment the synaptonemal complex (SC)*

In these demo images (image3a, 3b), the synaptonemal complex is labelled with ZYP1 and ASY1. Their localization domains are segmented, consecutively. The Surface tool is used whereby the parameters vary depending on the image. If the image has a high signal to noise ratio and no or little background staining, the default parameters proposed by Imaris are satisfying. If the immunostaining is noisy, or if the image resolution is low, or both, the parameters need to be adjusted to best capture the SC structure. Adjustment is particularly relevant for background subtraction, where seed size called ‘diameter of the largest sphere’ and threshold play an important role. In addition, if the image has not been masked before (Step 2), ASY1 and ZYP1 residual signals outside the chromosomes will be segmented. The surfaces capturing this noise can be filtered out in the last step of the creation wizard using size, or intensity. But depending on the image, the filtering criteria may not be sufficient to remove these biologically irrelevant surfaces, hence masking from step2 is strongly recommended in these cases.

The parameters used for our demo images were the following.

*Image 3a.*
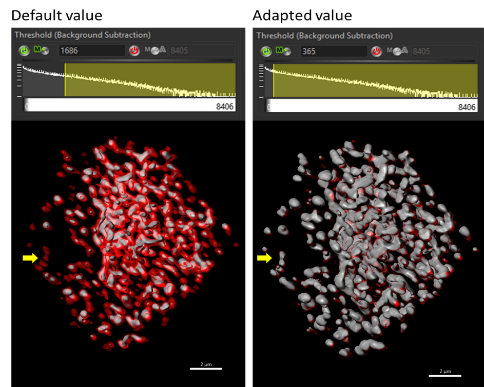
For ASY1 segmentation, chose the masked ASY1 channel, smoothing details are kept with the default value, background subtraction with 0.5um for the ‘diameter of largest sphere’. This value can be adjusted by measuring the width of ASY1 signal on different part of the image using the slice viewer and drawing a measurement line. Next, the threshold for background subtraction was adjusted to 365 to capture all the relevant signal – see image (the yellow arrow points towards part of the signal that was not captured using the default threshold value). This value will differ for images taken under different conditions / with different resolution. The criteria here is subjective but can be justified with the aim to not leave relevant ASY1 signal out.

For ZYP1 segmentation, the same approach was used, on the masked ZYP1 channel, using 0.2um as the diameter of the largest sphere and 200 for the threshold.

*Image 3b.* Only ZYP1 was segmented in this example, on the original channel since here no DAPI surface was created. Segmentation was done using 0.4um as the diameter of the largest sphere and 200 for the threshold. The surface was used to mask the HEI10 channel for the next step. (Edit/Mask/Channel 2, voxel outside to surface set to zero)

Note: masking the immunosignal of HEI10 with the SC surface makes sense only if the study targets specifically HEI10 foci associated with the SC. Association may differ between meiotic stages or deviate in mutant backgrounds. In these cases, masks may better be omitted at that step and HEI10 foci can be sorted at later stage according to their position with regards to the SC surfaces.

*Step 4- Segment crossover (CO) foci as spots*

Images 3a and 3b report on two distinct proteins involved in meiotic recombination namely DMC1 for DNA Strand Break (DSB) processing and HEI10 marking class I CO. Both occur as discrete foci that can be well segmented using the Spot tool. The strategy for both DMC1 and HEI10 segmentation (as for any other label of CO) is essentially the same with some adjustments of: (i) the starting spot size (estimated in the slice viewing mode), (ii) the axial (PSF) elongation of the signal in z, which depends on the imaging method and resolution of the image and (iii) the intensity threshold for spot detection.

In addition, there is the possibility to segment specifically the CO foci associated with one component of the SC. This can be done in two ways: (a) during spot creation, filter the spots according to their distance to the (biologically) relevant surface; (b) mask the CO channel with the (biologically) relevant SC surface, and use this masked channel for creating CO spots (see Note, Step3).

In our demo workflow we used the strategy (a) to segment HEI10 spots associated with ZYP1 specifically and the strategy (b) to classify DMC1 spots relative to ASY1.

*Image 3a* . Create a Spot object and follow the wizard. Select the DMC1 channel masked on DAPI surface. Activate the “shortest distance calculation” or “object-object statistics” (name depends on the Imaris version) to be able to measure the distances of the spots to the SC surface in later steps. Activate the ‘Classify spots’ option (optional).

Start spot size: xy diameter = 250µm; Z diameter (PSF elongation) = 500µm; Background substraction was not activated


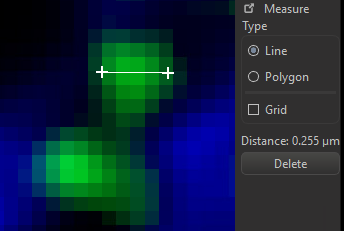
To estimate the expected spot size, switch to the Slice viewing mode, draw a line across a typical spot, read the diameter. Repeat this on a few spots. The size does not have to be an exact value but an estimation guiding the segmentation. (see image on the left; the white line is drawn in Imaris and measures a diameter of 255µm).

The z diameter (PSF elongation) depends on the image resolution (verified in section viewer) and voxel size (Edit/Image Property/Geometry)

The next step allows to filter the spots according to quality (criteria that considers intensity and local contrast). The default value identifies few spots, well separated and showing high signal intensity. If the image shows a high signal-to-noise ratio, it is meaningful to consider all possible spots hence to lower the quality threshold to 1, and to create classes later to assess the different properties of low vs high intensity spots. The effect of quality threshold is illustrated below. Here we chose a permissive quality threshold, and the spots were classified at the next step.

Classification allows to create classes of spots according to specific properties. This can be intensity levels, or distance relationships. As an example, here we classified DMC1 spots according to their intensity (sum), following automatic group creation based on the properties of the intensity distribution (with the middle class corresponding to spots centered around the median). See Figure 3f.


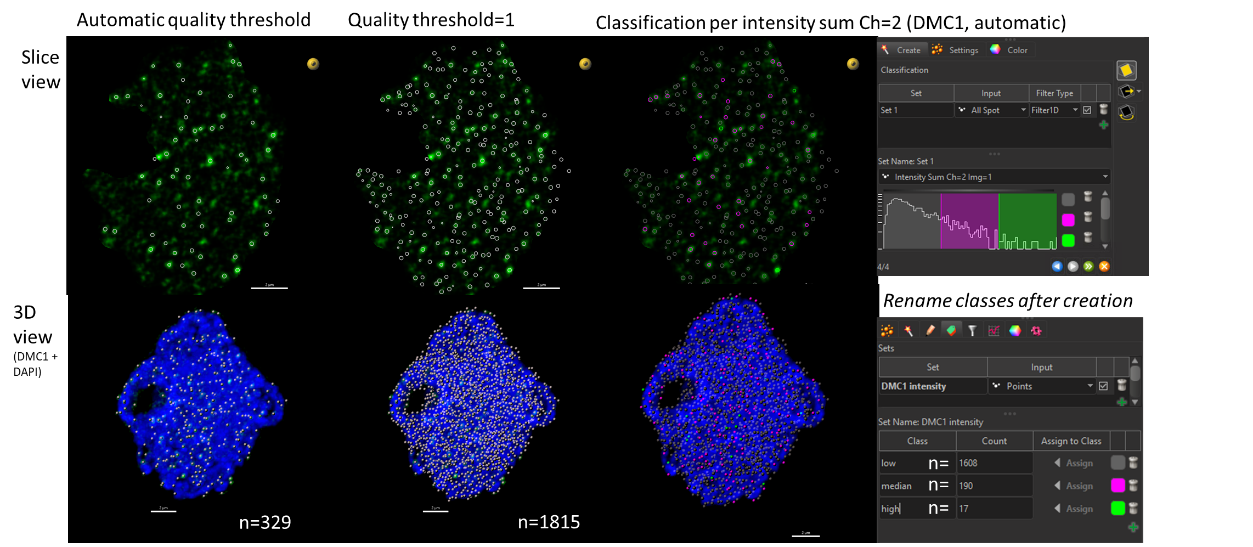


A second classification was done to distinguish the DMC1 spots colocalizing with ASY1 from those not colocalizing with it. For this, an ‘Set’ was added ( ‘+’ in the classification wizard) and the variable ‘shortest distance to ZYP1 surface’ was chosen. Two classes were made for spots inside the surface (negative values) and outside the surface (positive values) hence colocalizing with ASY1 (see Figure 3g).

*Image 3b*. Create a Spot object and follow the wizard. Select the HEI10 channel masked on ZYP1 surface. Activate the “shortest distance calculation” or “object-object statistics” (name depends on the Imaris version) to be able to measure the distances of the spots to the SC surface in later steps. Activate the ‘Classify spots’ option.


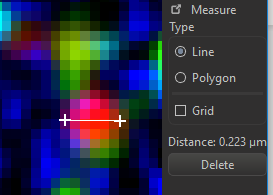
Start spot size: xy diameter = 220µm; Z diameter (PSF elongation) = 880µm; Background substraction was activated

Classification: HEI10 foci were classified based on signal intensity (Intensity sum Ch=5, masked HEI10 channel). The classification wizard allows to select ranges based on the intensity histogram but does not allow to directly select fraction based on customized quantiles. Here, we first chose the default class creations up to three, to complete the spot creation. Next, we customized the classes as follows: using the filter tab, we selected first the top 2% (above 1.94e6) and going back to the classification tab, we assigned them to the formerly created class C, which we renamed T1. We then repeated the operation for the second (intermediate) class corresponding to the next 2% (between 1.31e6 and 1.94e6) and assigned the spots to class B renamed as T2. Finally, the last class consisted in the remaining 96% (below 1.30e6) assigned to class A renamed as T3. See below


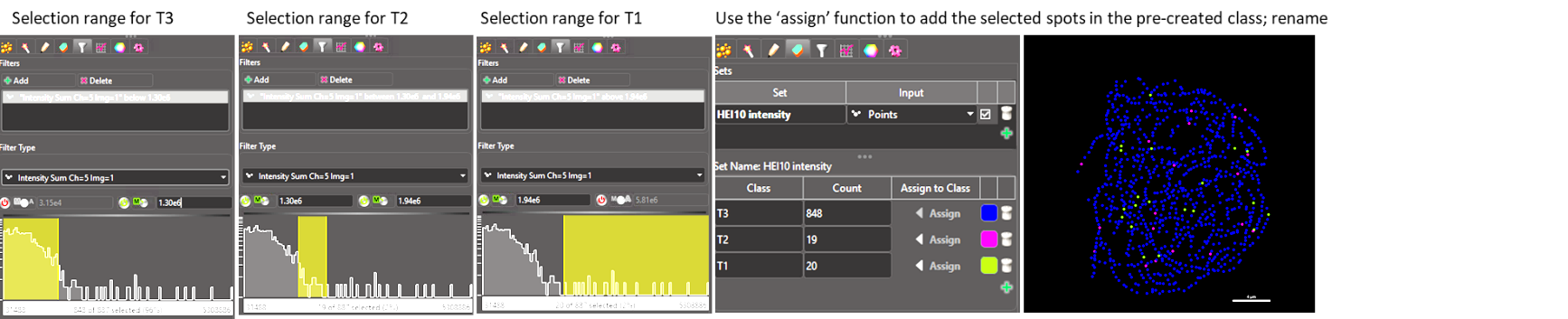


*Step 5 – Data Vizualisation*

The properties of the different DMC1 and HEI10 classes are explored in Vantage by plotting different statistics such as DNA (DAPI) intensity, SC (ZYP1, ASY1) intensity or distance relationships. Examples are shown in the main Figure and discussed in the text. Only 1D plots are shown here but 2D and 3D scatter plots are also possible to explore more complex relationships. In addition, classification using Machine Learning has the potential to identify different classes (classification using ML mode, not shown here, see software tutorials).
