# Supplementary material for: Image analysis workflows to reveal the spatial organization of cell nuclei and chromosomes
Source: Nucleus. 2022 Nov 29;13(1):277–99. doi: 10.1080/19491034.2022.2144013 (PMC9754023; doi:10.1080/19491034.2022.2144013)
Supplement: Supplemental Material [file KNCL_A_2144013_SM9221.zip › Supplemental File 4 Text and Table/Supplemental File 4 - Text_and_Table/Workflow 4-Text.docx]

# **Workflow 4 – Analysis of the spatial distribution of nuclear speckles & bodies**

This step-by-step image analysis workflow can be practiced with the training images, supplemental file 4- image 4a and image 4b.

Image 4a shows an *Arabidopsis* leaf nucleus stained for DNA with Hoechst580CP (Bucevičius et al., 2019) (channel Ch=1) and expressing the YFP-tagged SSSU protein (see main text). Because nucleus isolation poorly preserves the native YFP fluorescence, SSSU-YFP was immunostained using a GFP booster antibody (GFP-Booster ATTO488, ChromoTek (gba488-100), 1:2000 dilution, channel Ch=2). Sample preparation and immunolabeling was performed as described elsewhere (Ashenafi & Baroux, 2018). The image was acquired by STED microscopy (Leica SP8 STED 3x, using a 93x Glycerol, HC PL APO STED WHITE objective as described in Ashenafi and Baroux, 2018).

Image 4b shows a mouse nucleus (naïve pluripotent embryonic stem cells) stained for DNA with DAPI (grey, ch=1) and immunostained for a GFP-tagged chromatin protein (CP) forming nuclear bodies (green, ch=2) as well as H3K27me3 (red, ch=3). Samples were prepared as described (Bersaglieri et al., 2022) (Bersaglieri et al., 2022) and the image was acquired using confocal microscopy imaging (Leica inverse SP8, 63x glycerol HC PL APO CS2 objective).

The key steps and parameters are also summarized in the supplemental file 4- Table 4. When applied to other, similar images, these parameters must be adjusted as they highly depend on image resolution and quality (signal-to-noise ratio).

Workflow for Image 4a.

*Step1- Nucleus segmentation.*

The nucleus is segmented using the ‘Surface’ function of Imaris. If the nucleolus is located too close to the nuclear periphery, the manual segmentation should be used (see Workflow 1, step1). In this example, we performed automated segmentation with a smoothing factor of 10.15 and a user-defined intensity threshold (‘Absolute intensity’) on the DNA channel (Ch=1). The display is changed to transparent grey.

*Step1a (optional).* The nucleus surface can be used as 3D mask if the image is noisy. Use the Edit Tab/Mask All, set voxel outside to surface to 0. Unclick the option ‘duplicate channel’ (this avoids duplicating channels, a situation which makes the analysis of exported data cumbersome).

*Step2- Chromocenter segmentation.*

CC are best segmented with the Surface function, using the supervised, automated mode. Here, we used a smoothing factor of 0.15 and the option ‘background subtraction’ is used to improve the identification of regions with stark contrast in signal intensities. The quality and intensity threshold guiding the watershed-based algorithm is set manually to capture best the CC. This step is subjective and requires a user-based decision, which can be repeated, however, across images using similar threshold values. Touching CC can be separated using the option ‘split touching objects. During segmentation, at the filtering step, only objects with a size (area) >0.5 um2 are kept (alternatively, diameter or volume can be used as filtering criteria).

*Step3- Speckle segmentation.*

To segment SSSU signals forming small speckles, we use the ‘Spots’ function. Add a Spot object and follow the creation wizard. Select the option ‘object-object statistics’ (or ‘shortest distance calculations’ in versions earlier than 9.9) and omit the classification. Here, since SSSU speckles have a near constant size, we use the mode ‘spots of fixed size’. Select the channel of interest (here Ch=2). Next, the estimated diameter of the speckles to segment, here 0.320µm. To estimate the speckle diameter, we used the line measurement tool in the Slice Viewing mode (navigate through the Z stacks and set a line with two left mouse clicks across a nuclear speckle, read the value on the right panel, repeat on other speckles). Back in the spot creation wizard, at the next step, apply the thresholding method ‘Quality’ and lower it down to 1 to capture all possible structures (those can be classified at later stage). Possibly, however, images with lower signal-to-noise ratio require a more conservative threshold or background subtraction in the first step.

*Step4- Data export for customized analysis.*

The signal and distance variables associated with the created objects (spots and surface) are called ‘Statistics’ in Imaris. Select the appropriate ones in the Preferences/Statistics for Spots and Surfaces, respectively. For exporting these variables at once for all objects, place the nucleus and spots in one folder (use the Create folder button for this). In the statistics Tab of the folder, select the Export All button. Prior to data export, make sure that (i) the option ‘object-object statistics’ is activated in all objects (this feature is necessary for distance measurements), (ii) the objects’ names have a consistent spelling throughout the images, (iii) the transiently created channels (masks) are deleted.

*Example of statistics of interest* *for* *our aim*: (i) for spots and surface: Intensity Sum, Intensity Standard Deviation, Intensity Mean, (ii) for spots: volume, shortest distance to Surface, distance to nearest neighbor, averaged distance to top 3, 5 or 9 neighbours, (iii) for surfaces: distance to surface, volume, area, sphericity, ellipticity, distance measurements.

*Step5- Data visualization.*

The data can be visualized using ImarisVantage: to plot data from several images at once select several images in the Arena and select ‘Add a new plot’ in the top menu bar. Follow the wizard and explore the plot options. Alternatively, for plotting image-normalized data, especially regarding signal intensities, open the exported data using DataViz tool (see Wokflow 1, step 8 and video 2).

Workflow for Image 4b.

*Step1- Nucleus segmentation.*

The nucleus is segmented using the ‘Surface’ function of Imaris. Select the option ‘Object-object statistics’ (or ‘shortest distance calculations for Imaris versions earlier than 9,9). If the nucleolus is located too close to the nuclear periphery, the manual segmentation should be used (see Workflow 1, step1). In this example, we performed automated segmentation with a smoothing factor of 0.180 µm (surface grain size), without background subtraction and a manually defined ‘Absolute intensity threshold’ of 10 on the DNA channel (Ch=1).

The Surface was used as a 3D Mask to remove signals outside the nucleus in all three channels (Surface/Edit/Mask All/ ‘duplicate channel’ was opted out, set voxels outside surface set to zero, repeated for each channel). The display is changed to transparent grey.

*Step2- Chromocenter segmentation.*

CC are best segmented with the Surface function, using the supervised, automated mode. Select the option ‘Object-object statistics’ (or ‘shortest distance calculations for Imaris versions earlier than 9,9). Here, we used a smoothing factor of 0.2 µm and the option ‘background subtraction’ is used with 1µm for the ‘Diameter of Largest sphere’. The quality threshold guiding the watershed-based algorithm is set manually to capture best the CC. This step is subjective and requires a user-based decision, which can be repeated, however, across images using similar threshold values. Here we used 28.8 as lower threshold. At the (last) filtering step, only objects with a number of voxels >1240 were kept (small objects were not retained). Alternatively, this filtering step can be done using the object diameter. The display was adjusted to cyan to differentiate the CC from the nucleus color.

*Step3- Bodies segmentation.*

Both H3K27me3 and the CP protein form nuclear bodies of varying size. We therefore used the Surface tool to segment them. Select the option ‘Object-object statistics’ (or ‘shortest distance calculations for Imaris versions earlier than 9,9). The initial parameters were kept to the automatic values (surface grain size =0.145 µm, background subtraction, diameter of largest sphere = 0543 µm). Threshold intensities were adjusted (8 for CP signal, 10 for H3K27me3) and the option ‘enable split touching objects’ was selected (seed point= 0.8 µm). The quality threshold was kept to the default value (CP) or manually set to 2 (H3K27me3) to capture smaller bodies of lower intensities. At the final, filtering step by size (number of voxels), only objects with >500 (CP) or 33 (H3K27me3) were retained.

To segment SSSU signals forming small speckles, we use the ‘Spots’ function. Add a Spot object and follow the creation wizard. Select the option ‘object-object statistics’ (or ‘shortest distance calculations’ in versions earlier than 9.9) and omit the classification. Here, since SSSU speckles have a near constant size, we use the mode ‘spots of fixed size’. Select the channel of interest (here Ch=2). Next, the estimated diameter of the speckles to segment, here 0.320µm. To estimate the speckle diameter, we used the line measurement tool in the Slice Viewing mode (navigate through the Z stacks and set a line with two left mouse clicks across a nuclear speckle, read the value on the right panel, repeat on other speckles). Back in the spot creation wizard, at the next step, apply the thresholding method ‘Quality’ and lower it down to 1 to capture all possible structures (those can be classified at later stage). Possibly, however, images with lower signal-to-noise ratio require a more conservative threshold or background subtraction in the first step.

*Step4- Data export for customized analysis.*

The signal and distance variables associated with the created objects (spots and surface) are called ‘Statistics’ in Imaris. Select the appropriate ones in the Preferences/Statistics for Spots and Surfaces, respectively. For exporting these variables at once for all objects, place the nucleus and spots in one folder (use the Create folder button for this). In the statistics Tab of the folder, select the Export All button. Prior to data export, make sure that (i) the option ‘object-object statistics’ is activated in all objects (this feature is necessary for distance measurements), (ii) the objects’ names have a consistent spelling throughout the images, (iii) the transiently created channels (masks) are deleted.

*Example of statistics of interest* *for* *our aim*: (i) for spots and surface: Intensity Sum, Intensity Standard Deviation, Intensity Mean, (ii) for surfaces: distance to surface, volume, area, sphericity, ellipticity, all possible distance measurements, overlapped volume and overlapped volume ratios.

*Step5- Data visualization.*

The data can be visualized using Imaris Vantage: to plot data from several images at once select several images in the Arena and select ‘Add a new plot’ in the top menu bar. Follow the wizard and explore the plot options. Alternatively, for plotting image-normalized data, especially regarding signal intensities, open the exported data using DataViz tool (see Workflow 1, step 8 and video 2).

# References

Ashenafi, M. S., & Baroux, C. (2018). Automated 3D gene position analysis using a customized Imaris plugin: XTFISHInsideNucleus. *Methods in Molecular Biology*, *1675*, 591–613. https://doi.org/10.1007/978-1-4939-7318-7_32

Bucevičius, J., Keller-Findeisen, J., Gilat, T., Hell, S. W., & Lukinavičius, G. (2019). Rhodamine–Hoechst positional isomers for highly efficient staining of heterochromatin. *Chemical Science*, *10*(7), 1962–1970. https://doi.org/10.1039/C8SC05082A

Bersaglieri, C., Kresoja-Rakic, J., Gupta, S., Bär, D., Kuzyakiv, R., Panatta, M., Santoro, R. (2022). Genome-wide maps of nucleolus interactions reveal distinct layers of repressive chromatin domains. *Nature Communications*, 13, 1483. https://doi.org/10.1038/s41467-022-29146-2
