# Supplementary material for: Image analysis workflows to reveal the spatial organization of cell nuclei and chromosomes
Source: Nucleus. 2022 Nov 29;13(1):277–99. doi: 10.1080/19491034.2022.2144013 (PMC9754023; doi:10.1080/19491034.2022.2144013)
Supplement: Supplemental Material [file KNCL_A_2144013_SM9221.zip › Supplemental File 5 Text and Table/Supplemental File 5 - Text_and_Table/Workflow 5-Text.docx]

# **Workflow 5 – Analysis of metaphase chromosome ultrastructure using volume measurement of oligo-FISH labeled regions.**

The supplemental file 5- image 5 is a 3D-SIM image stack of a barley 5H metaphase chromosome stained for the DNA by DAPI. The centromere, NOR, telomeres, subtelomeres were labeled by specific FISH probes. An interstitial region of the long arm was stained by oligo-FISH probes in different colors (Kubalová et al., 2021).

The key steps and parameters of image analysis are also summarized in the supplemental file 5- Table 5. When applied to other, similar images, these parameters must be adjusted as they highly depend on image resolution and quality (signal-to-noise ratio).

*Step 1- Signal intensity adjustment.*

Starting with the DAPI channel the signal intensities were optimized for all different colors using the sliders of the ‘Display adjustment’ tool.

*Step 2- Chromosome and FISH signal segmentation.*

The chromosome was segmented using the ‘Surface’ function of Imaris. Automated segmentation with user-defined intensity threshold works well on the DNA channel to define the boundary of the chromosome. For it, apply the ‘Threshold’ (Absolute Intensity) slider to adapt the surface to the DNA signal intensity. To obtain a smoother surface the default ‘Surface Detail’ of 0.0794 was set to 0.1. Afterwards, remove segmented background objects by moving the ‘Filter’ slider. Use the finalized chromosome surface to mask all other differently colored channels to get rid of background signals outside the chromosome volume. Instead, mask all other channels (‘Mask All…’) separately by activating ‘Duplicate channel before applying mask’ and ‘setting the voxels outside to surface’ to 0.00. Use the newly established masked channels to generate surfaces for all other FISH signals as described for the DNA channel.

*Step 3- Animation.*

To visualize the chromosome and FISH signals via different views a 3D movie was generated (Movie X) using the Imaris tool ‘Animation’. Adapt the movie speed through changing the ‘Frames’ number.

*Step 4- Select volume statistics.*

Insert all surfaces into a newly created folder (‘Add new group’). Select for this group ‘Statistics’ > ‘Detailed’ > ‘Specific Values’ > ‘Volume’.

*Step 5- Export data*.

Export all values via ‘Export all Statistics to File’ to an excel file. After exporting the volumes from several chromosomes, the data can be used for statistical calculations.

*Step 6- Data visualization.*

Use the ‘Vantage‘ tool to present the selected and grouped surface volumes or mean intensities in a diagram. The desired surface order can be arranged via ‘Surpass Objects and Labels’.
