# Supplementary material for: Image analysis workflows to reveal the spatial organization of cell nuclei and chromosomes
Source: Nucleus. 2022 Nov 29;13(1):277–99. doi: 10.1080/19491034.2022.2144013 (PMC9754023; doi:10.1080/19491034.2022.2144013)
Supplement: Supplemental Material [file KNCL_A_2144013_SM9221.zip › Supplemental File 6 Text and Table/Supplemental File 6 - Text_and_Table/Workflow 6 -Text.docx]

# **Workflow 6 – Analysis of centromeres and telomeres’ positioning**

This step-by-step image analysis workflow can be practiced with the training images, supplemental file 6- image 6a-TypeI, - image 6a-TypeII (Figure 6b-f) and supplemental file 6- image 6b (Figure 6g-j).

The images 6a (Type I and II) are that of barley endosperm flow-sorted nucleus after fluorescence *in situ* hybridization (FISH) with centromeric (channel Ch=1, magenta) and telomeric (Ch=2, yellow) probes. DNA was stained using DAPI (Ch=3, gray). The image was acquired by an AxioImager Z2 (Zeiss) epifluorescence microscope equipped with a DSD2 spinning disk (Andor).

The image 6b is that of an isolated leaf nucleus stained for DNA with DAPI (channel Ch = 1) and with FISH probes labeling centromeres (magenta = Ch2) and telomeres (green = ChS1), and 35S rDNA loci (Cy3 = ChS2). The image was acquired by a laser scanning microscope (Zeiss Axio Observer Z1) with laser scanning unit LSM 780.

The key steps and parameters are also summarized in the supplemental file 6 - Table 6. When applied to other, similar images, these parameters must be adjusted as they highly depend on image resolution and quality (signal-to-noise ratio).

**Workflow for Images 6a**

*Step1- Segmentation of centromeric and telomeric FISH signals*

The centromeric and telomeric FISH signals are segmented using the ‘Spots’ function. Each channel is segmented separately using automatic segmentation settings, with minor modifications.

*Step 1a – Segmentation of centromeric signals*


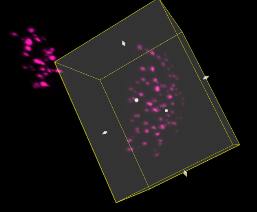

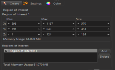


The options ‘different spot sizes’ and ‘classify spots’ are unselected, but ‘object-object statistics’ are selected. Chose channel 3.

If the image contains more than one object (nucleus), then the option ‘segment only a region of interest' is mandatory (Figure 1). Alternatively, the image can be cropped in 3D around the region of interest (Main Menu/Edit/Crop 3D).

Figure 1


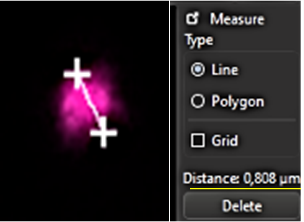
At the next step, the creation tool requires to define the spots size. To provide this value switch from 3D view to slide view mode (2D) and measure manually the size of one selected spot. For this, draw a line capturing a spot diameter, and read the distance in the left (or right)-upper part of the screen (Figure 2). Here, the value amounts 0.808 µm. Type the approximate value in the window ‘estimated XY diameter’, mark ‘background subtraction’ and unmark ‘model PSF-elongation along-Z axis’, to create spherical spots. If your image has a poor axial (z) resolution, activate this latter option and enter the estimated axial length of the signal, ellipsoid spots will be created.

Figure 2


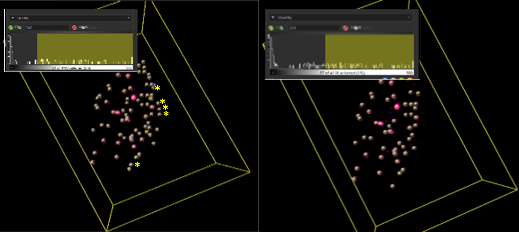
At the next step, the ‘quality’ threshold is adjusted manually, to 131 for the demo image, thereby excluding spots with weak intensities which are not true FISH signal in our interpretation (Figure 3, left panel with a conservative threshold and yellow stars showing spots of weak intensities, right panel with a conservative threshold capturing unambiguous FISH signal).

Figure 3


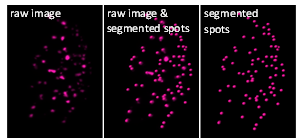
The spots should overlap with all signals (Figure 4). Imaris can customize colors. For both raw images and segmented spots, use ‘base color’ mode, which allows changing and adjusting the colors according to RGB color code.

Figure 4

*(b) Segmentation of telomeric signals*


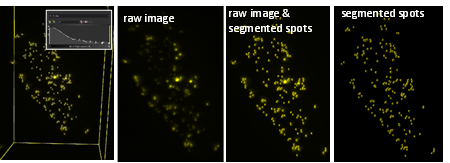
Segmentation of telomeric FISH signals follows the same principles as for centromeric spots. The signals are smaller and the ‘estimated XY diameter’ was around 0.3 µm in our image. In addition, the fluorescence intensity of the signals is weaker. To cover the signals, the threshold for ‘quality’ was chose at 0.74 for the demo image (Figure 5).

Figure 5

*Step2- Segmentation of the nucleus*

The nucleus surface is segmented using the ‘Surface’ function. Automatic segmentation is selected, the option ‘classify surfaces’ is unmarked, but ‘object-object statistics’ is kept. This demo image contains two nuclei (objects), to segment only one of them, the option ‘segment only a region of interest’ is mandatory.

The smoothing factor is kept to the automatic value, here surface grain size= 0.130 µm.

*Note: For irregular shapes as on this image showing grooves and invaginations, it is best to keep a low smoothing.*


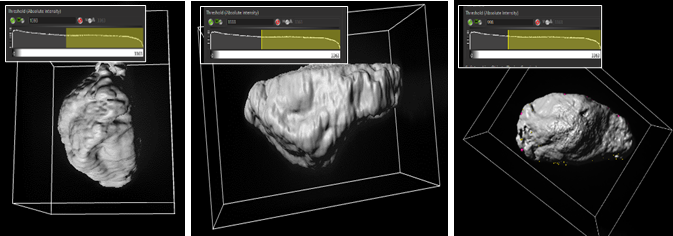
To render the surface of the nucleus, we used the option ‘absolute intensity’. The threshold was adjusted manually to 1247. During this step, it is important to rotate the object and look at it from different perspectives (Figure 6). Already segmented spots should be inside the nucleus surface.

Figure 6

*Step3 - Rendering*

For better visualising the position of spots inside the nucleus, use a transparent coloring mode of the surface (Figure 7).


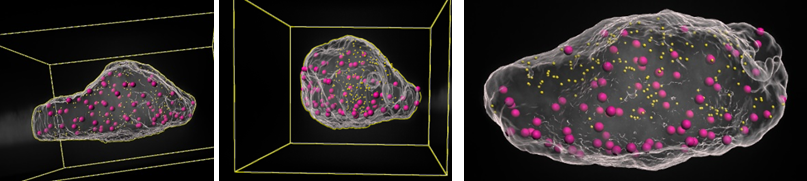


Figure 7

In addition, the ‘Clipping’ tools allow to visualize the spot distribution in the nucleus space and relative to the surface (Figure 8).


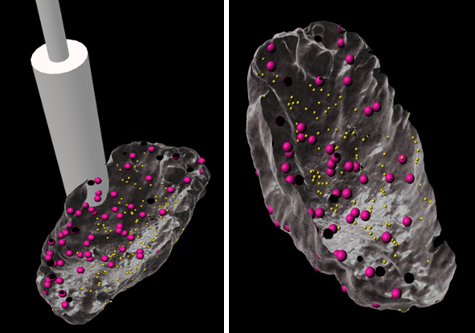


Figure 8

*Step 4 – Export Data*

Spots and surface segmentation allow for automatic measurement of numerous signal intensity and geometrical parameters informing on the position of spots relative other spot groups and to the nucleus surface. Here we exported the following statistics (Main Menu/Edit/Preferences/Statistics/Spots and Surface): (i) ‘Shortest distance to spots’, (ii) ‘Shortest distance to surfaces’ (iii) ‘Averaged distance to top 3, 5 or 9 neighbours’, (iv) ‘Distance to nearest neighbor’; and for surfaces only: ‘volume’ and ‘Diameter’.

The statistics are exported in each object using the Statistics Tab and Save All options. Alternatively, a Folder object is created (object Menu bar) and the spots and surface objects are moved (drag and drop) into the folder. Select the folder and the Statistics Tab, then Save All. All data from all objects will be exported at once in one folder.

*Note: The ‘overall’ statistics file or sub-tab, in each object, provides the number of segmented spots, which can be useful for scoring FISH signals per nucleus (or immunostaining).*

*Note: while the diameter in X,Y,Z dimensions of the ellipsoid can be obtained automatically, it is possible to obtain a quick readout with manual measurement using the tool ‘Measurement points’ in pair mode. Place the Measurement Point object in the folder, the AB distance will also be exported* (Figure 9).

*
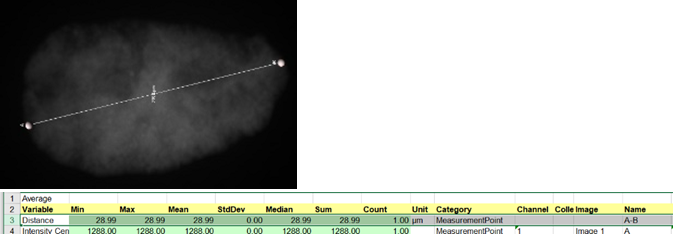
*

Figure 9

*Step 5- Data exploration and visualisation*

At the final step, each image can be converted to a series of graphical plots using the ImarisVantage module. This module enables 1D- and 2D-plots on single, or multiple images using selected objects and Statistics, with ready-to-go graphics. For plotting data of a single image, select ‘Vantage’, in the Surpass view. To plot data from several images, select the relevant images in the Arena and select the action ‘Add new plot’ in the top menu. Examples of plots below:

*
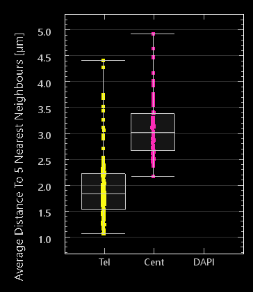
*
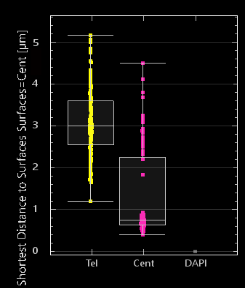
*
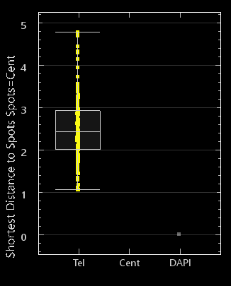

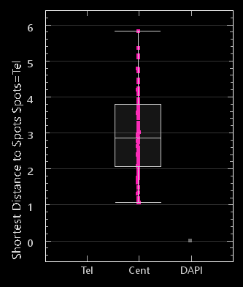
*

*
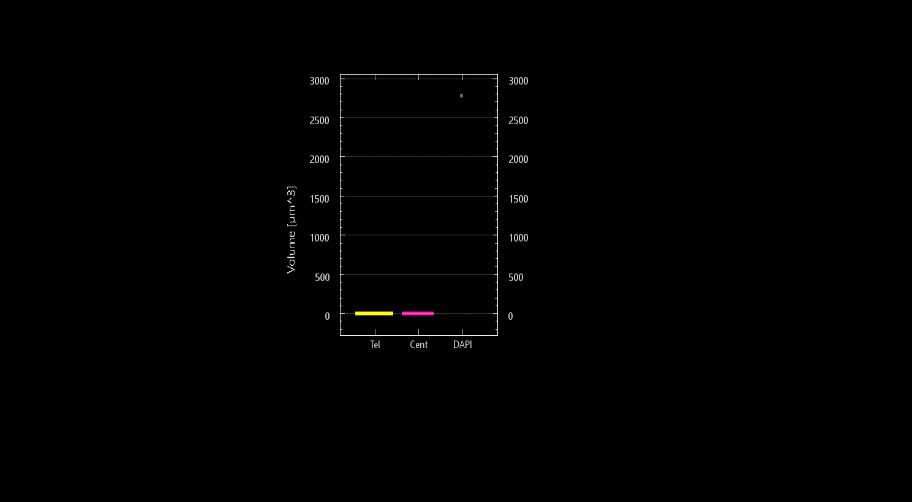

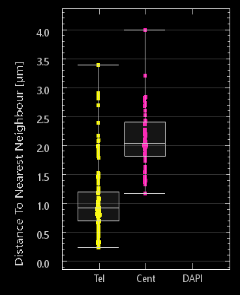

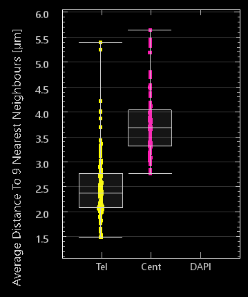

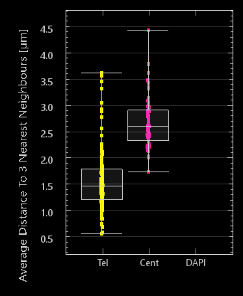
*

Here, for normalization of the data we use the exported statistics and processed them in excel. For instance, we normalized the distance between centromeric and telomeric spots with the nucleus diameter as the nucleus size would influence the dispersion of these signals.

**Workflow for Image 6b**

*Note: –* prior to step 1, two additional (optional) steps can be performed.

*- Deconvolution* can be performed prior to image analysis to improve image resolution.

*- 3D cropping* is useful to select the part of the image containing the nucleus to analyse. For this, use the Main Menu/Edit/3D Crop function and enter the ranges of X, Y, and Z coordinates into the corresponding fields or adjust the region of interest manually using the handles directly in the viewer window.

*Step 1 – Nucleus segmentation*

*Automatic segmentation -* The nucleus is segmented using the Imaris function ‘Surfaces’. This function can segment the object automatically or manually. For an automatic segmentation on this image, chose a smoothing factor 0.45, no background subtraction, and adjust the intensity threshold to best capture the nucleus stained by DAPI (ca 12000).

The nucleolus, when visible, is yielding a problematic result in automatic segmentation. When located close to the periphery of the nucleus, segmentation can result in a large invagination. When located towards the interior of the nucleus, segmentation creates a hollow surface with an outer boundary (nucleus) and an inner boundary around the nucleolus. For distance calculations at later stages, Imaris does not differentiate the inner and outer surface boundaries. For this reason, either try a higher smoothing factor (for instance 0.81 on our image) or prefer manual segmentation (below). A higher smoothing factor result in a bigger surface, less precisely following the DNA stain contour and may lead to overestimated distances of objects to the surface periphery.

*Manual segmentation.* This approach is preferred to create a plain surface with a single (outer) surface boundary precisely following the DNA-stained contour. To start manually contouring the surface choose the option ‘Skip automatic creation, edit manually’ and draw precise contours according to boundaries of individual planes. It is not necessary to draw contours in every plane and if the nucleus has a regular shape, 6-8 contours are sufficient (covering the top, middle and bottom parts). But in case of an irregular shape, the more contours drawn, the more accurate the surface will be. To correct a Surface, if the result is not satisfying, go to the ‘Settings’ Tab of the surface and recompute the surface with the manual mode. The previously drawn contours are kept and additional contours can be drawn to improve the Surface quality.

*Step 1b (optional) - 3D masking.*

In case of background noise (FISH signals) outside the nucleus, use the Nucleus surface to mask them away before Step 2: in the Surface, open the Edit Tab, select the option 'Mask All', unselect duplicate channel (or if you keep this option to duplicate the original channel, delete the redundant channels before data export), select the first FISH signal channel, set voxels outside to Surface to zero. Apply and repeat on the other FISH signal channels

*Step 2 – Telomeric signals segmentation.*

The computer-generated ‘Surface - automatic’ function was used for segmentation of telomeric signals without using background subtraction, and using a smoothing factor of 0.120 um for this image. The intensity threshold was adjusted manually (12887) to best capture the signals. A filtering step is applied to remove surfaces created around signal evaluated as background noise (subjective, user-based but based on biological relevance). Here, we removed surface objects with less than 2.3 voxels (average) corresponding to objects that do not have a biologically relevant size.

Note that for other images where telomeric signals are in close proximity, those can be separated by the ‘Split touching objects’ function. This option supports the separation of two or more objects identified by automatic segmentation as one.

*Step 3 – Centromeric signal segmentation.*

Centromeres/chromocenters are identified as fluorescent signals of a centromeric repeat probe. Centromere segmentation is processed using the computer-generated ‘Surface - automatic’ function. It creates solid objects in order to visualize shape, volume and position of a centromeric/chromocenter loci. The absolute intensity threshold is user-defined to achieve the best caption of the centromeric signals. This step is very subjective and requires a user-based judgement. Centromeric signals in close proximity can be separated by the ‘Split touching objects’ function.

*Step 4 – 35S rDNA signal segmentation.*

The 35S rDNA probe has been shown for detection of nucleoli (e.g., Pecinka et al., 2004; Shan et al., 2021). Based on the intranuclear position of a 35S rDNA locus, it is possible to identify the nucleolus. Nucleolus (35S rDNA) segmentation is done by the automatic ‘Surface’ function, creating solid objects around the nucleolus. The absolute intensity threshold is user-defined for the best caption of 35S rDNA signals. Signals in close proximity can be separated by the ‘Split touching objects’ function.

*Step 5 – Spots creations (optional).*

The function ‘Spots’ is suitable for modelling the centromeres and telomeres as 3D dots. Spots enable additional statistical values such as the average distance to the next 3, 5 or 9 neighbouring spots, not available with Surfaces (at least in Imaris 9.8 and 9.9). For this, create a spot object and choose the source channel of interest. Here, the option ‘Classification of objects’ can be skipped. The size of spots is based on ‘Estimated XY diameter’, which can be measured in ‘Slice view’. Spots with diameter smaller than estimated XY diameter will be not detected. If the image shows a poor axial (Z) resolution, chose the option ‘Model PSF-elongation along Z-axis – estimated Z diameter’ and enter the estimated length of the spots along the Z-axis. The displayed size of the spots can be also reduced after the creation (Spots/Settings).

*Step 6 – Data analysis*

Select the surfaces and spots statistics of interest in the Main Menu/Preferences/Statistics. Make sure that all objects have the option ‘Object-object statistics’ (Imaris 9.9, called ‘Shortest Distance Calculation’ in former versions) as active. Verify that all objects’ names have consistent spelling. Export the Statistics per object (one by one), in Statistics/Save All. Easier is, however, to create a folder object, drag and drop all Surfaces and Spot objects in this folder. Select the folder and in the Statistics Tab, Save All.

*Step 7 – Data Visualization*

Data can be visualized for one or several images using Imaris Vantage. For plotting data of one image, go to ‘Vantage View’ in the Surpass viewing mode. For plotting data of several images, select them in the Arena (ctrl+shift) and select ‘Add New Plot’ on the main menu bar. Vantage allows to effectively visualize and analyze the relevant statistical data, this view allows to create clear, compact and colorful high-quality plots. The highly interactive visualizations help to understand the complexity of the statistical data by identifying hidden patterns and trends. For computing a 1D plot with information about the shortest distance of telomeric and centromeric signal borders to the border of nucleus, function ‘Shortest distance to surfaces’ is used. Distances are positive for surfaces that are entirely outside the nucleus surface. Distances are negative for surfaces that are entirely inside the nucleus surface. Distances are zero for surfaces that intersect the nucleus surface. Function ‘Shortest distance to spots’ can be used to compute 1D plot of the shortest distance from the border of nucleus to the center of all spots from the ‘Spots object’. Keep in mind that for this statistical outputs is necessary to have surfaces with only the nucleus boundary and not the nucleolus boundary (See Step 1). Otherwise, the distances to Surface are not discriminative of distances to the outer or inner boundaries of the surface.

**References**

Armstrong, SJ, Franklin, FC & Jones, GH. 2001. Nucleolus-associated telomere clustering and pairing precede meiotic chromosome synapsis in *Arabidopsis thaliana*. Journal of Cell Science 114, 4207–4217.

Pecinka, A, Schubert, V, Meister, A, Kreth, G, Klatte, M, Lysak, MA, Fuchs, J & Schubert, I. 2004. Chromosome territory arrangement and homologous pairing in nuclei of *Arabidopsis thaliana* are predominantly random except for NOR-bearing chromosomes. Chromosoma 113, 258–269. doi: 10.1007/s00412-004-0316-2

Hurel, A, Phillips, D, Vrielynck, N, Mezard, C, Grelon, M & Christophorou, N. 2018. A cytological approach to studying meiotic recombination and chromosome dynamics in *Arabidopsis thaliana* male meiocytes in three dimensions. Plant Journal 95, 385–396. doi: 0.1111/tpj.13942

Shan, W, Kubová, M, Mandáková, T & Lysak, MA. 2021. Nuclear organization in crucifer genomes: nucleolus-associated telomere clustering is not a universal interphase configuration in Brassicaceae. Plant Journal 108, 528-540. doi: 10.1111/tpj.15459
