# Supplementary material for: Image analysis workflows to reveal the spatial organization of cell nuclei and chromosomes
Source: Nucleus. 2022 Nov 29;13(1):277–99. doi: 10.1080/19491034.2022.2144013 (PMC9754023; doi:10.1080/19491034.2022.2144013)
Supplement: Supplemental Material [file KNCL_A_2144013_SM9221.zip › Supplemental File 7 Text and Table/Supplemental File 7 - Text_and_Table/Workflow 7-Text.docx]

# **Workflow 7 - Analysis of division angle during mitotic division**

This step-by-step image analysis workflow can be practiced with the training image, supplemental file 7- image 7. The image is that of a barley (*Hordeum vulgare*) cell expressing fluorescently tagged histone variants: H2B (CFP-H2B, channel Ch=1, cyan) and centromeric histone H3 (RFP-CENH3, channel Ch=2, magenta) (Kaduchová and Pecinka, unpublished). The image was acquired with a Leica TCS SP8 confocal microscope equipped with the Leica Application Suite X (LAS-X) software version 3.5.5 and Leica Lightning module (Leica, Buffalo Grove, IL, USA). (Leica Microsystems, Wetzland, Germany).

The key steps and parameters of the image analysis workflow are also summarized in the supplemental file 1- Table 1. When applied to similar images, these parameters must be adjusted as they highly depend on image resolution and quality (signal-to-noise ratio).

*Step 1- Display adjustment.*

Figure 1. Adjustment of volume, visualization in MIP and Blend modes.


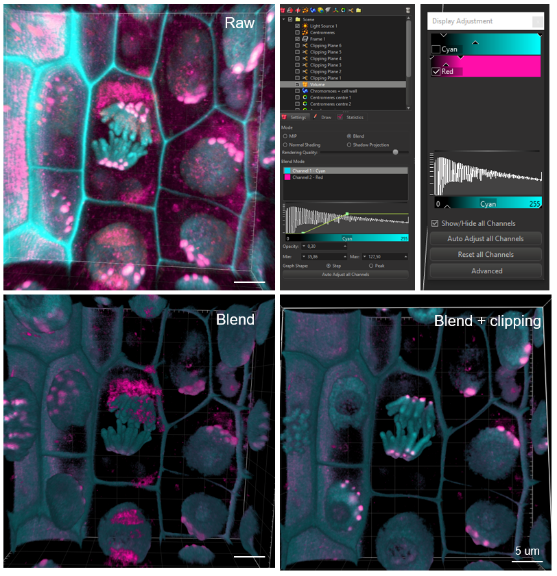


Live imaging of fluorescently tagged proteins often yields low-level intensity, especially if image acquisition is kept with conservative parameters preserving signal quality during scanning. To obtain a better visualization of the actual 3D image and facilitate inspection before further analysis, it is helpful to first adjust the display using the Display adjustment Tool. Here, we chose the ‘Blend mode’ to better render the chromosomes and plant cell walls in 3D. Manually adjust the Min, Max intensities and Opacity for each channel (Figure 1).


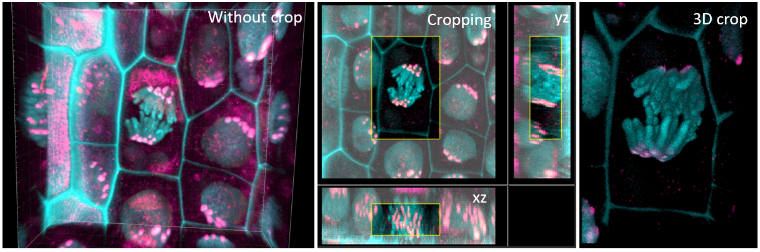
*Step 2 - 3D cropping.*

Figure 2. Cropping of cell layer 3D space focusing of ROI containing dividing cell.

To focus on one cell at a time, use the ‘Crop 3D’ option (Main Menu/Edit). Select the region of interest using the manual sliders (Figure 2).

*Step 3 – Centromeres segmentation*


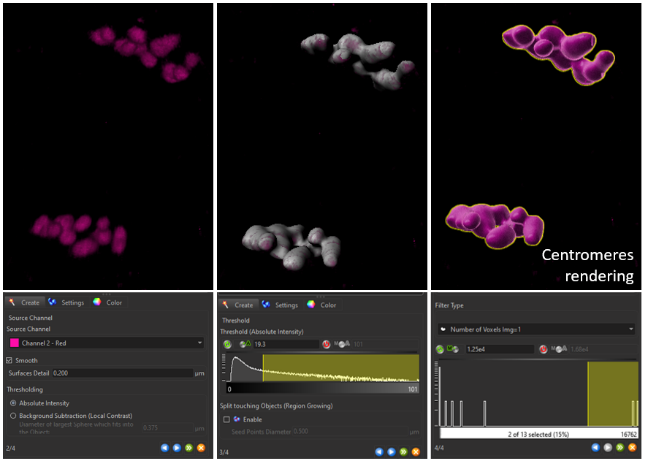
*Step 3a, b, (c) – Centromeres and Cell walls (chromosomes) segmentation.*

Figure 4. Surface rendering of ROI in less (Surface 1) and stricter (Surface 2) rendering threshold.


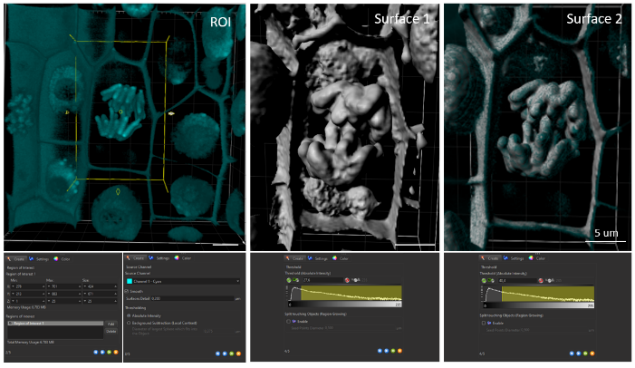


Figure 3. Surface rendering of centromeric groups.

5 um

Centromeres are segmented using the ‘Surface’ Tool of Imaris. Here we used a smoothing factor (Surface detail) of 0.2um, no background subtraction and absolute intensity thresholding (19.3 in this example). As small objects are created around low-intensity noise signals, a filter step is applied at the end to select only the centromeres. The rendering is set to transparent to visualize the spot at a later stage.

*Step 4 – Cell wall segmentation*

In this example, cell wall autofluorescence is detected in the channel set for detecting H2B-CFP (marking the chromosomes). This property is used to create a surface capturing the cell’s boundary. Create a Surface object, segment automatically, without background subtraction and a smoothing factor of 0.15 um (for this image), using absolute intensity thresholding method. At the next step, the threshold is lowered compared to the automatic value of 37 to separate well the chromosomes and cell wall (Figure 4). Filtering is used to remove smaller objects from the final segmentation. Non-desirable objects created during segmentation can be deleted after creation by selecting them and in Surface/Edit/Delete. Chromosomes can be kept for the next step (optional); if not, they should be deleted.

*Step 5 (optional) – Chromosome rendering*

Segmented chromosomes can provide helpful information about the division orientation and stage. Chromosomes have been segmented in the former steps. Select the chromosome surface objects (ctrl+left mouse key to select both), in the Edit Tab, select Duplicate. Rename the new surface object. Delete the chromosome objects in the previous Surface to keep only the cell wall object. The rendering can be set to transparent (optional) (Figure 5).


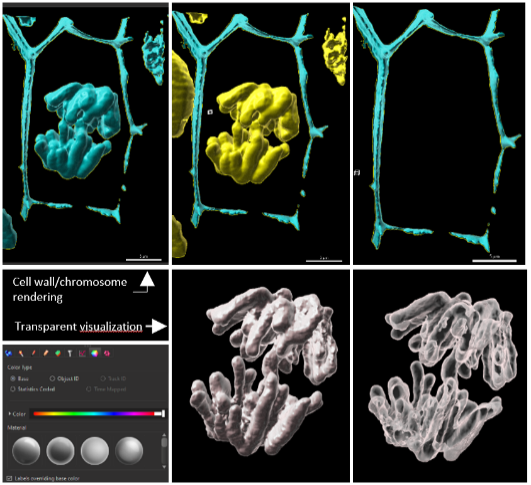


Figure 5. Deleting of additionally rendered structures, solid vs. transparent visualization modes.

In other (but similar images), if the chromosomes are not well segmented at step 4, delete them at step 4 and create a new surface focusing the segmentation performance on the chromosomes, and delete the other objects at the filtering step or after creation.

*Step 6 – Setting the measurement points and axes*

Create a new object ‘Measurement points’ and choose the mode ‘Polygon’. The AB and BC lines are set under two different modes (below and Figure 6)

For setting the Chromosome axis (CHA) given by A-B points, we used ‘Intersect with channel (Ch=2, magenta) ‘ and ‘Centre of Object’. Move the point setting tool controlled by the mouse on the image on the centromere object. The option selected before will allow to dock (by clicking once) an A point in one surface and a B point (second click) into the other centromeric surface, in both cases at their center-of-mass.


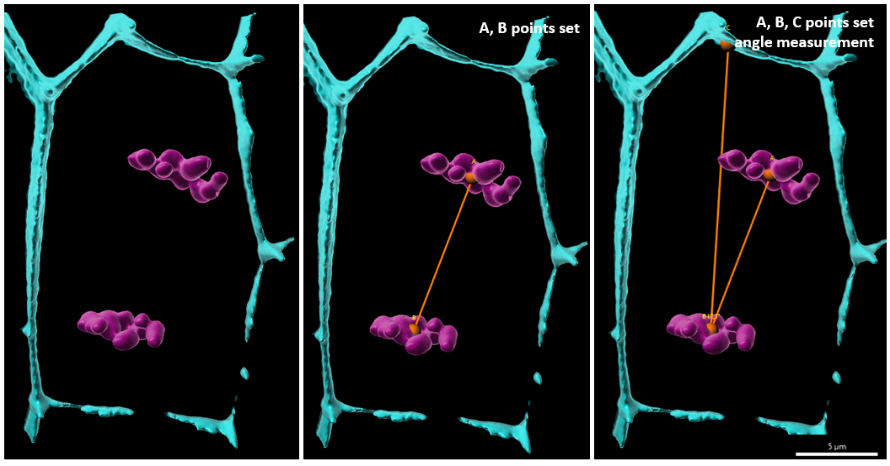


Figure 6. Setting of measurement points A, B and C

For setting the Cell elongation axis (CEA), select again the ‘Settings’ Tab from the same ‘Measurement point’ object and change the mode to ‘Intersect with channel’ (Ch=1, cyan) and ‘Surface of Object’. With the point setting tool, browse over the image and add a measurement point C at the top part of the cell wall, such that the BC line is parallel to the cell’s elongation axis. The position of the C point can be re-adjusted after creation (keyboard keys indicated in the creation wizard).

*Step 7 – Angle measurement*

The angle formed at the basis of AB and BC can be displayed (Settings) and read in the Statistics Tab/Detailed.
