# Supplementary material for: Image analysis workflows to reveal the spatial organization of cell nuclei and chromosomes
Source: Nucleus. 2022 Nov 29;13(1):277–99. doi: 10.1080/19491034.2022.2144013 (PMC9754023; doi:10.1080/19491034.2022.2144013)
Supplement: Supplemental Material [file KNCL_A_2144013_SM9221.zip › Supplemental_File_1_DataViz_Guidelines.pdf]

DataViz

User Manual

Version: 08.11.2022

!! download the latest version at:
https://github.com/barouxlab/DataViz

Universität Zürich[UZH]

Célia Baroux, IPMB
Devin Routh, S3IT

User manual – ‘How to ‘ ?

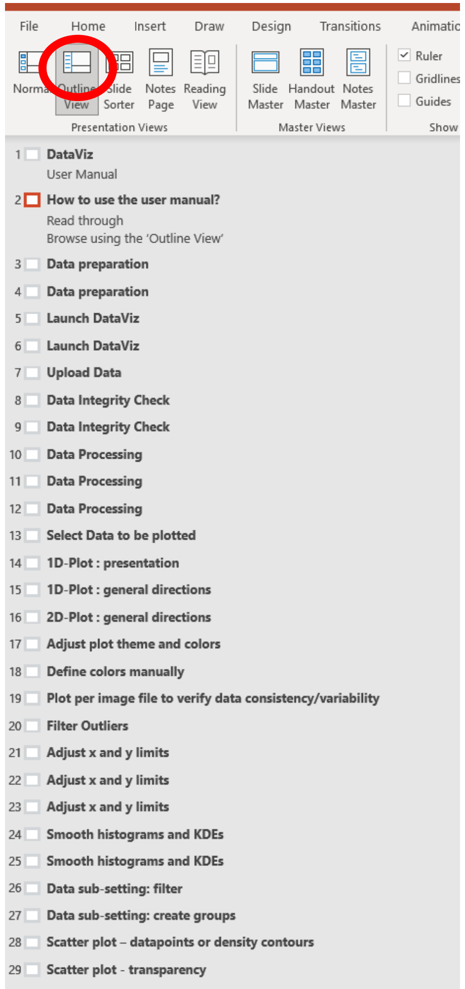

- Browse through specific topic using the ‘Outline View’
- Check for latest update of the user manual on https://github.com/barouxlab/DataViz
- Check the FAQ in DataViz for troubleshooting

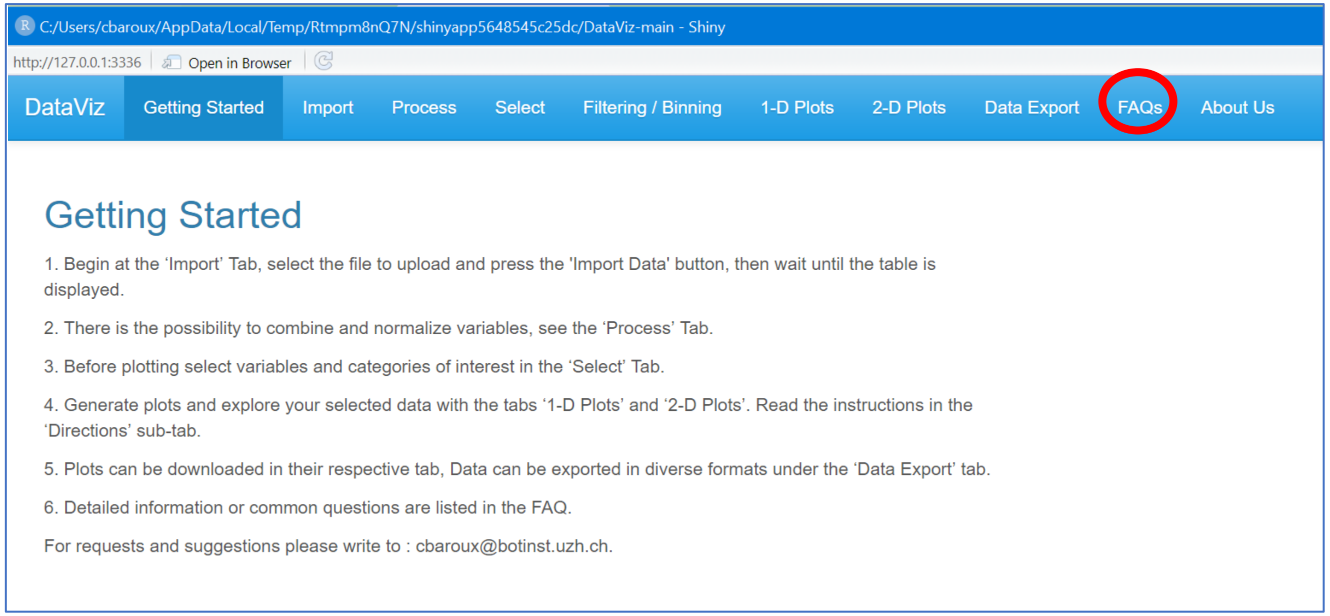

Data preparation

- **Using data exported from Imaris**

- DataViz was tested with data exported from Imaris 9.8 and 9.9
- Image Data must contain a surface called 'Nucleus' as this is a reference object in several calculations
- Image Data must be organized as follow: Genotype > Treatment> Image
- If several genotypes, the treatment folders must be duplicated with identical names
- The root folder containing the data is converted to a .zip file

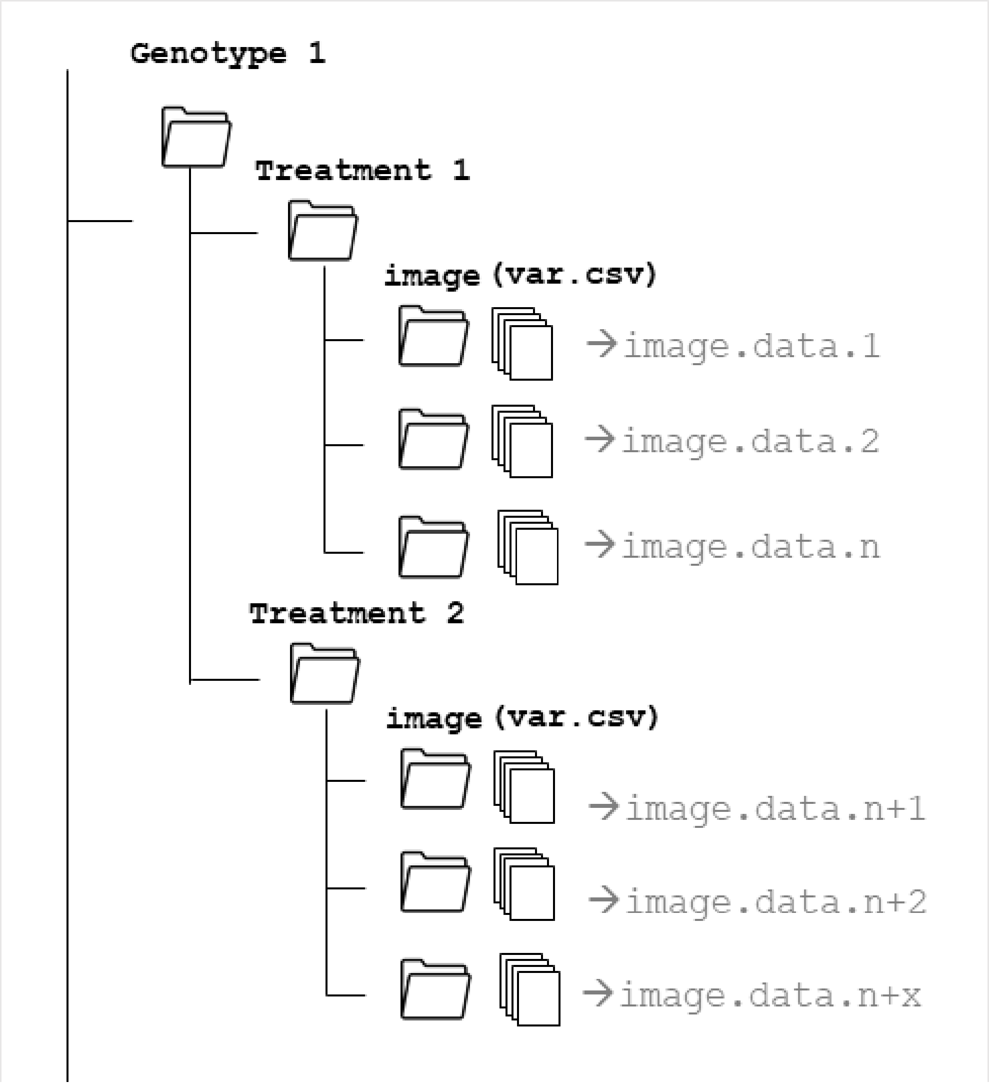

Data preparation

- **Using data exported from a different software**

    - In principle DataViz can handle any data organized according to the sample dataframe uploaded on github/barouxlab/DataViz: make sure to organize the data according to this template

    - The requirement of an object called 'Nucleus' is also valid for normalisations. If no normalization necessary, then DataViz will plot the raw data as labelled in the dataframe

Launch DataViz

- Open your browser, go to:
- https://github.com/barouxlab/DataViz

- Open the 'README' file. Read the instructions

- In the section 'running the application' , copy the lines of code *, Paste them In R (or R studio). Run

*
```
if(!"shiny" %in% rownames(installed.packages()))
install.packages("shiny"); library(shiny)
runUrl("https://github.com/barouxlab/DataViz/archive/main.zip")
```

- There is also the possibility to clone the repo locally – see infos in the README file

Launch DataViz

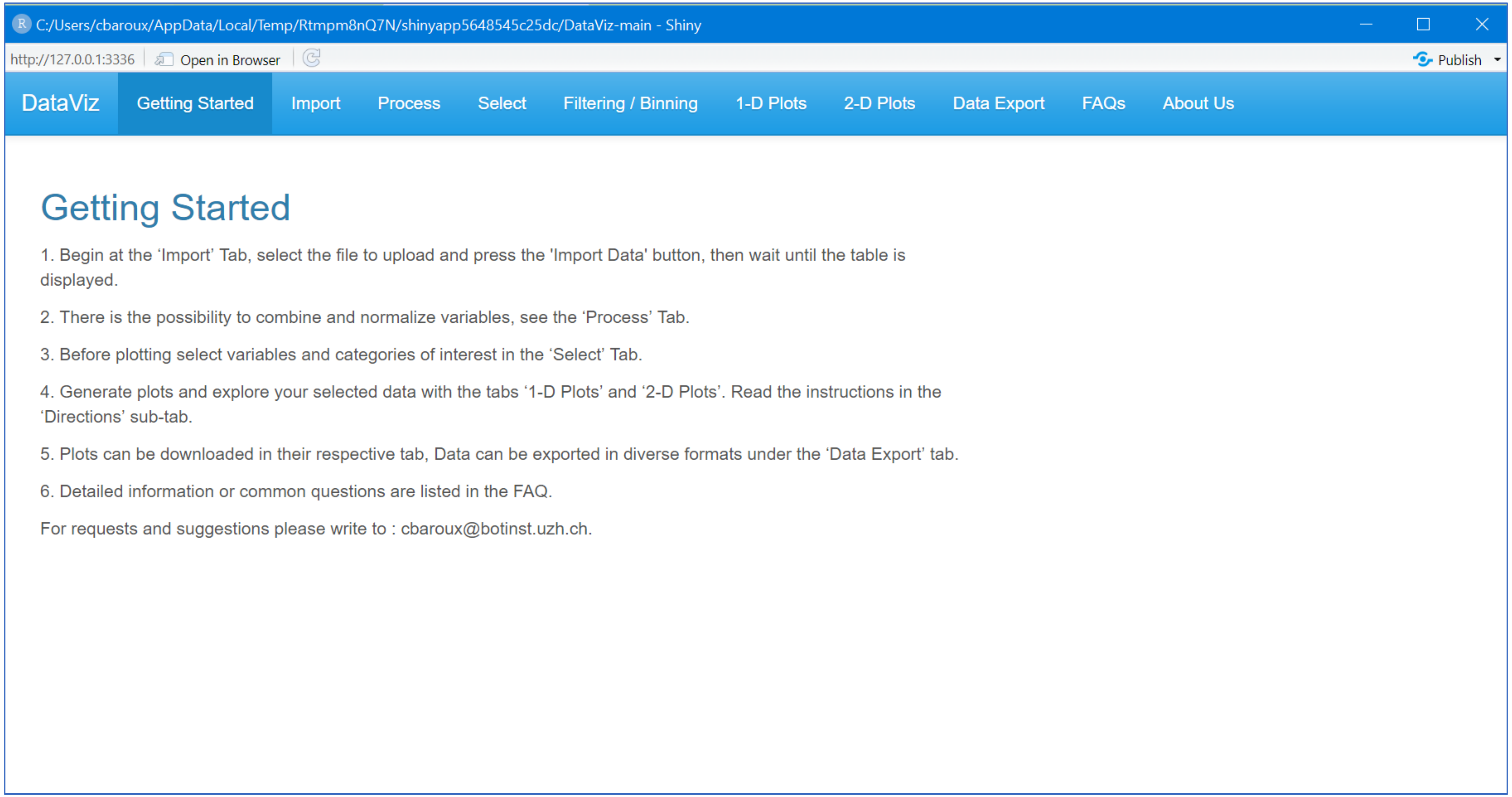

Upload Data

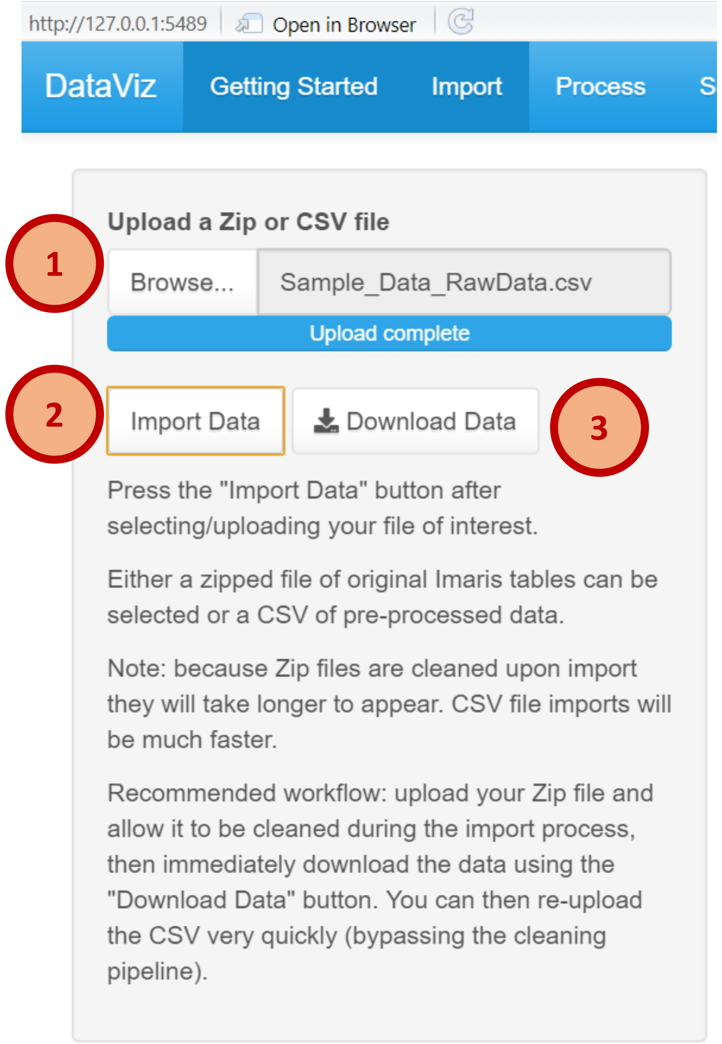

1. Browse to locate your zip folder. Confirm 'Open'
2. When the bar indicate 'Upload complete', Press 'Import Data'

Wait a few minutes – the import includes a cleaning function, data integrity check and creates a dataframe compiling all the data. This can take a few minutes depending on data size and computational power

3. When the table appears on the right, Press Download data

- *If you resume the work at a later stage, with a new session of R, you can upload and import the Raw Data file, the import will be much faster*
- *Note: some windows system seem not to recognize the csv format of the file. In that case, the system crashes at opening. Add manually the '.csv' extension to your filename*

Data Integrity Check

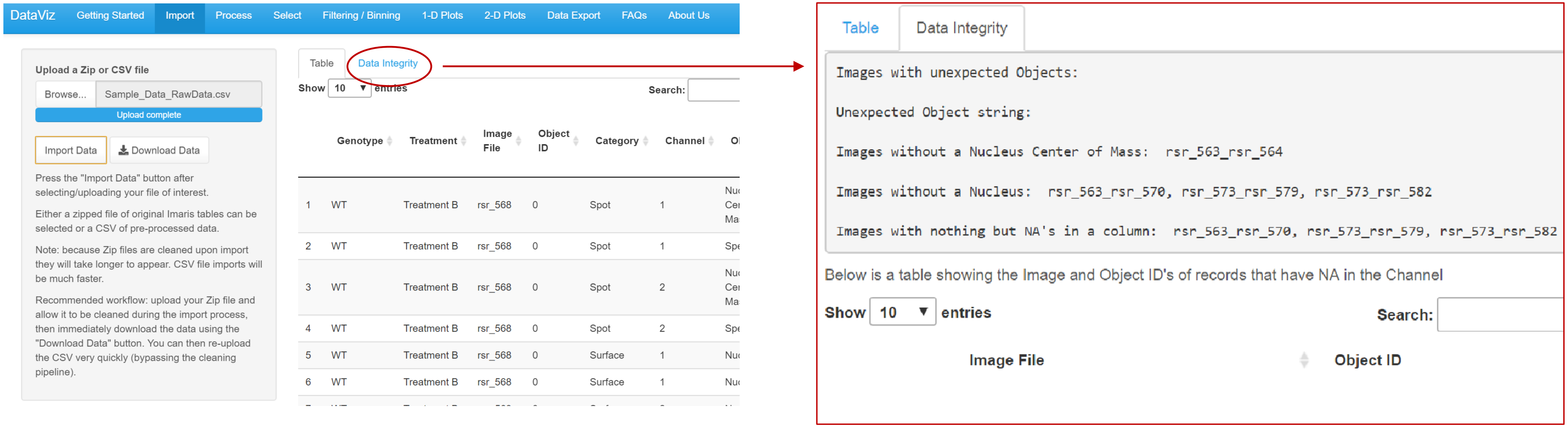

Once the dataframe is uploaded, check the ‘Data Integrity’ Tab – some inconsistency in the data may prompt the user to go back to the images and export steps – or can continue with data analysis knowing that not all variables will be present

Data Integrity Check

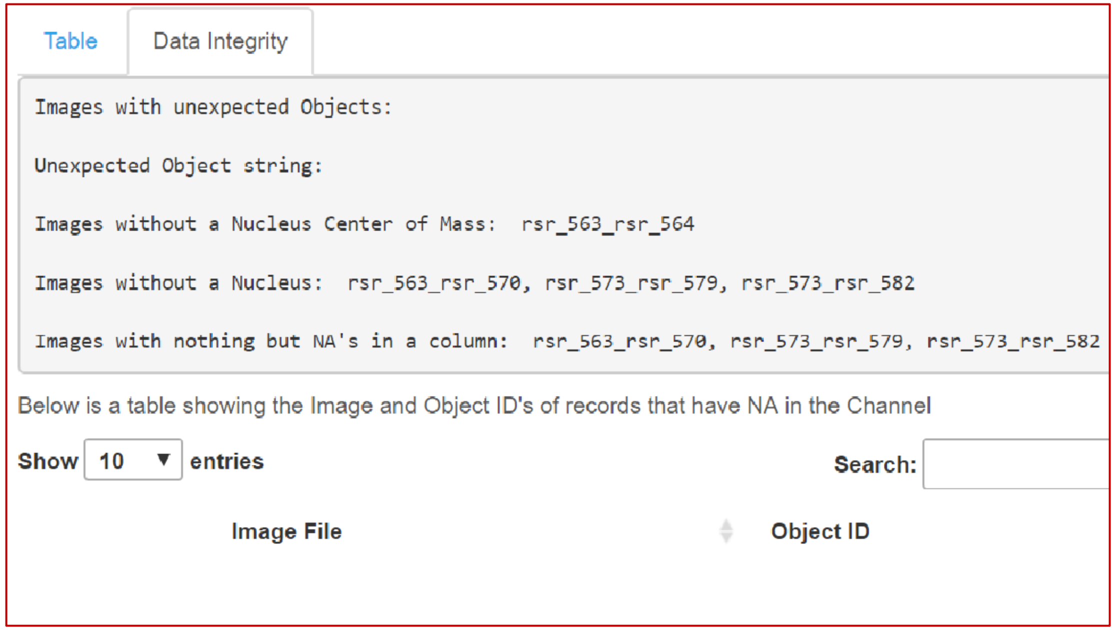

In this example (left):

- some images are missing an object called ‘Nucleus’ hence intensity normalization will not be performed for these images
- other images are missing a Center of Mass hence the normalization of distances will not be done for the objects in these images
- Some images are missing some statistics, hence NA in one of the column (which statistics can be checked in the data table)

Data Processing

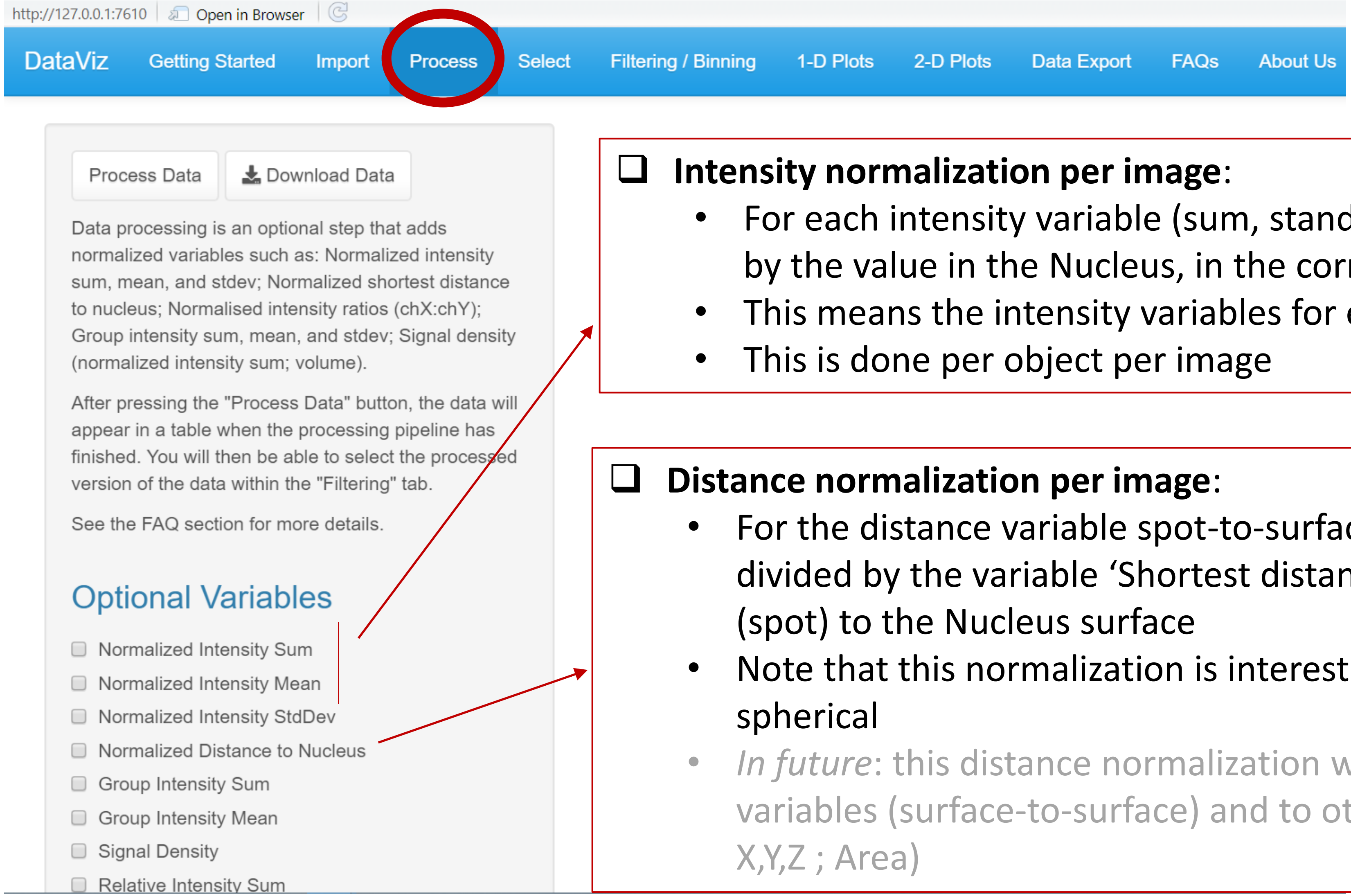

❑ **Intensity normalization per image**:
- For each intensity variable (sum, standard deviation), the value is divided by the value in the Nucleus, in the corresponding channel (Ch).
- This means the intensity variables for each Nucleus become =1
- This is done per object per image

❑ **Distance normalization per image**:
- For the distance variable spot-to-surface, for each object, the value is divided by the variable ‘Shortest distance’ of the Nucleus’ Center of Mass (spot) to the Nucleus surface
- Note that this normalization is interesting only if the nucleus is near spherical
- *In future*: this distance normalization will be customizable to other variables (surface-to-surface) and to other metrics (eg Nucleus diameter X,Y,Z ; Area)

Data Processing

- **Channel Ratio**:
  - for each object (spot, surface)
  - option for Intensity Sum and Intensity Mean
  - Customised ratio of signal intensity between channels of choice
  - Example: Ch2:Ch1, Ch3:Ch2, Ch4:Ch1 …
- **Group Sum**:
  - For each image, for each intensity variable (sum, standard deviation), per object category, the values of all object are added in the corresponding channel (Ch).
  - Example: Group Intensity Sum ‘CC’ (Ch=1) = Sum of the Intensity Sum of all CC in Channel 1
- **Signal Density**:
  - For each object, each channel, the normalized intensity sum is divided by the volume
- **Object Count**:
  - For each image, the number of objects in one category is counted
  - Example: number of chromocenter (CC) per Nucleus

Data Processing

- **Normalised distances**
  - Requirement: spot object called ‘Nucleus Center Of Mass’ (created during image segmentation)
  - For each image, the shortest distance of the spot = ‘Nucleus Center Of Mass’ to the surface = ‘Nucleus’ is taken as a reference distance
  - For each object, the ‘spot-to-surface’ shortest distance is divided by the reference distance
- **Normalised Intensities**
  - Requirement: surface object called ‘Nucleus’
  - For each intensity variable (sum, mean, standard deviation), for each object, the value is divided by the value in the ‘Nucleus’, in the corresponding channel
  - As a consequence, the normalized intensity in the ‘Nucleus’ becomes = 1 , and that of an object is a fraction of it
- **Relative Intensity Fraction (RIF)**
  - For each image, the RIF is the Group Intensity Sum divided by the Intensity Sum in the ‘Nucleus’ in the corresponding channel
  - Note: for the chromocenters, in the DNA staining channel, this corresponds to the RHF (Relative Heterochromatin Fraction)
- **Relative Volume Fraction (RVF)**
  - For each image, the RVF is the Group Intensity Volume divided by the Volume of the ‘Nucleus’

Select Data to be plotted

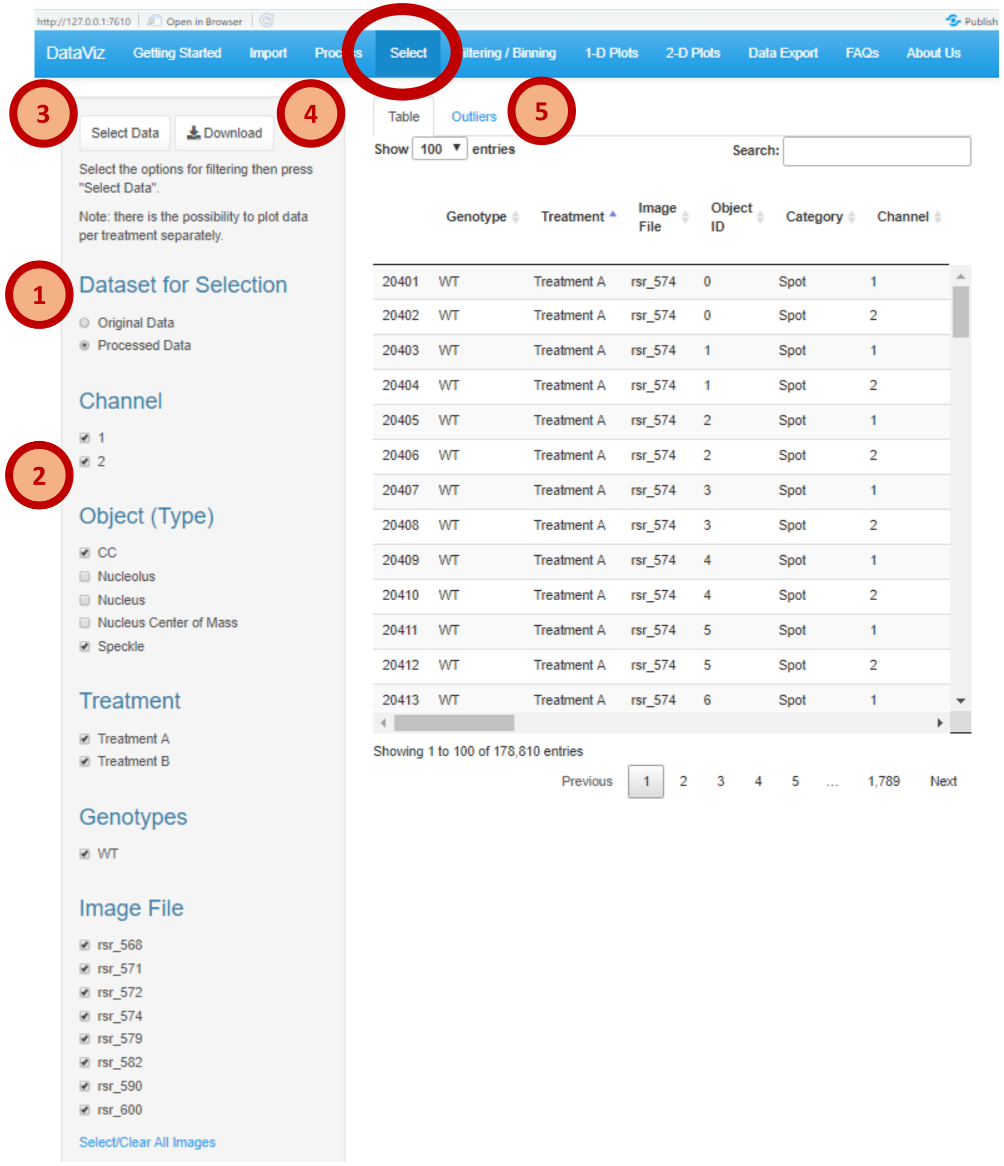

	Genotype	Treatment	Image File	Object ID	Category	Channel
20401	WT	Treatment A	rsr_574	0	Spot	1
20402	WT	Treatment A	rsr_574	0	Spot	2
20403	WT	Treatment A	rsr_574	1	Spot	1
20404	WT	Treatment A	rsr_574	1	Spot	2
20405	WT	Treatment A	rsr_574	2	Spot	1
20406	WT	Treatment A	rsr_574	2	Spot	2
20407	WT	Treatment A	rsr_574	3	Spot	1
20408	WT	Treatment A	rsr_574	3	Spot	2
20409	WT	Treatment A	rsr_574	4	Spot	1
20410	WT	Treatment A	rsr_574	4	Spot	2
20411	WT	Treatment A	rsr_574	5	Spot	1
20412	WT	Treatment A	rsr_574	5	Spot	2
20413	WT	Treatment A	rsr_574	6	Spot	1

1. Chose the data to be handle: original or processed data
→ If the data imported are already processed (from another session), chose 'original' data

2. Chose the channel, object, treatment, genotype and image file to use for plotting.
→ Think carefully of what needs to be plotted.
→ Selection can be changed dynamically while elaborating plots.

3. Press the 'Select Data' button, a table of selected data appears (right)

4. It is possible to download this data subset for additional analyses in third-party software (eg statistical analyses)

5. Inspect outliers (optional)
→This step can be performed only after a first round of plotting. In case outliers are visible, it is possible to filter them out .
→ For this, in the 'Outliers'Tab: select the variable, generate outliers, select 'yes' to the 'Remove outliers' option, and press 'Select Data' (3) again

1D-Plot : presentation

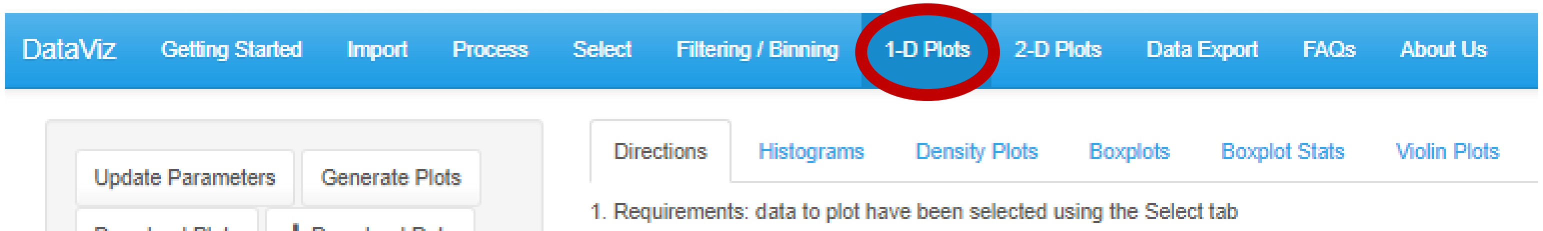

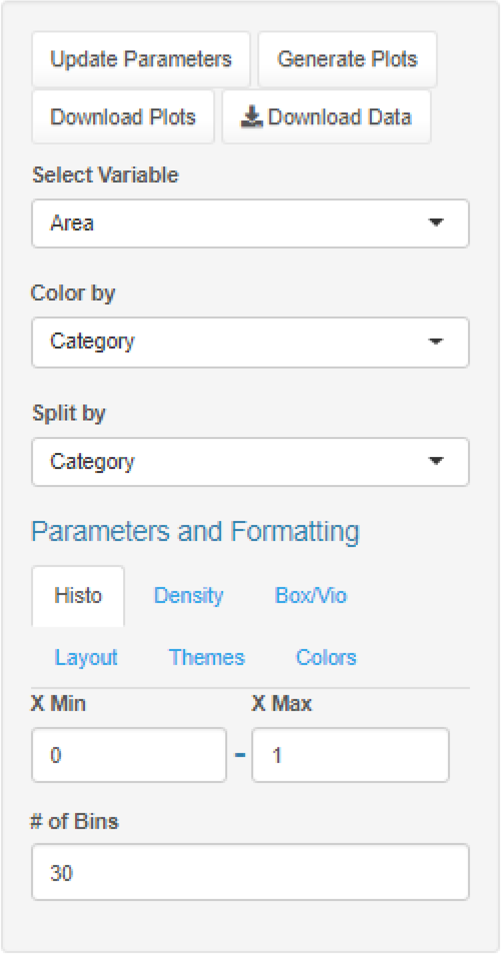

- 1-D plots consist in Histograms, Density Plots, Box Plots and Violin Plots
- Plots parameters and format of the plotting area are adjusted using the Tabs in the section ‘Parameters and formatting’
- ‘Update parameters’ will (re)set the plot parameters based on the selected variable and category for coloring/splitting
- It is important to press ‘Update parameters’ then ‘Generate Plots’ each time another variable or category for color/split is chosen.
- If plot parameters are adjusted, press ‘Generate Plots’ to refresh the plot area
- Box plots statistics deliver the plot’s descriptions
- ‘Download Data’ creates a csv file corresponding to the data plotted (=data subset)

1D-Plot : general directions

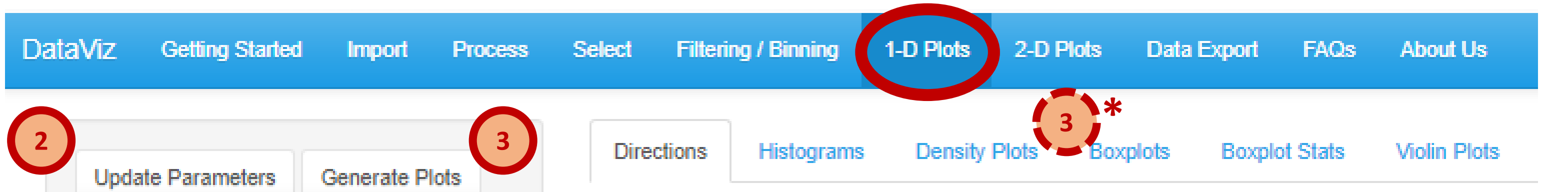

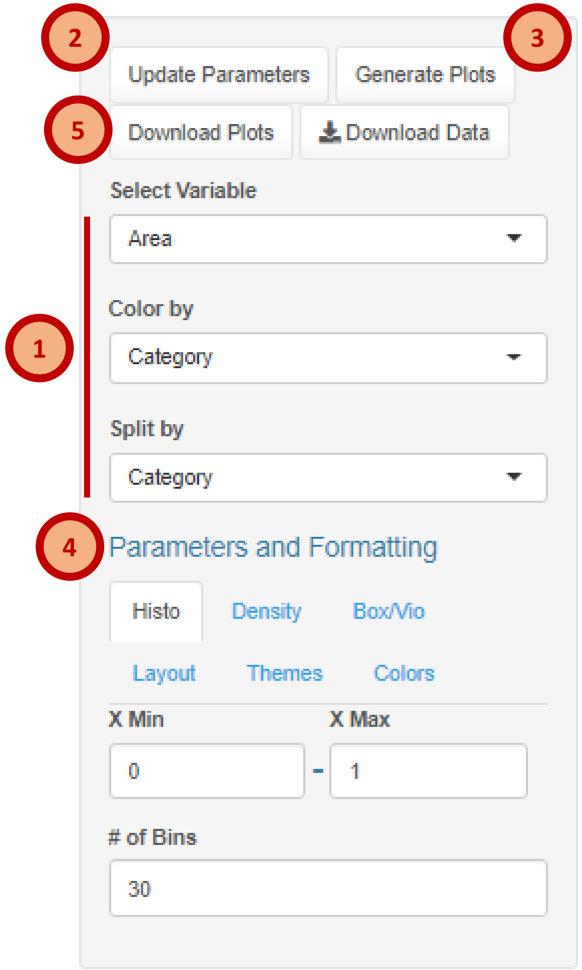

1. Select the variable to be plotted, the category for coloring and plot splitting in the pull down menus
2. Press ’Update Parameters’
3. Press ‘Generate Plots’ and select one of the Plot type in the Sub-Tab area (*)
4. Adjust the plot parameters and formatting
   - Specific plot parameters (x, y axes) in the corresponding sub-Tab
   - Layout: set the size of the plots and the number of plots per raw
   - Themes: set the background color and presence/absence of gridlines
   - Colors: chose or customize the color palette
5. Once the plots are ready, the plots can be downloaded (Download Plots) as well as the data subset used for the plots (Download Data) ; Boxplot statistics are in the subtab ‘Boxplot Stats’

2D-Plot : general directions

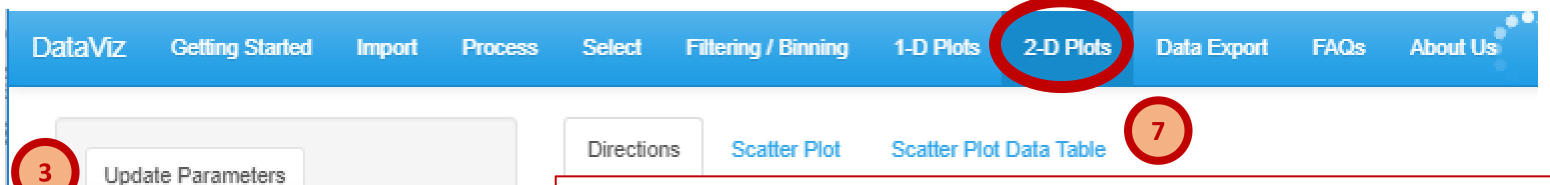

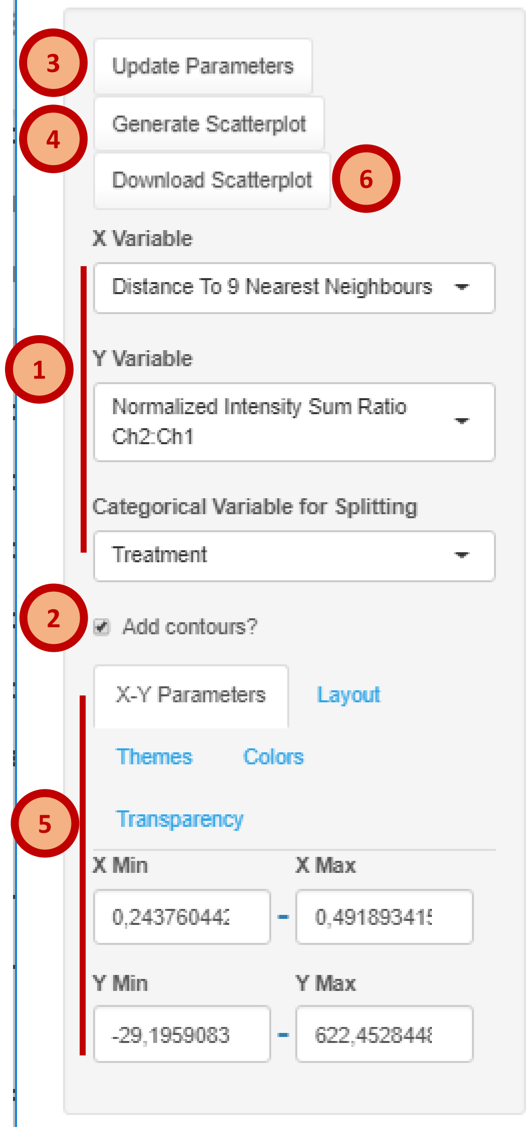

1. Select the X and Y variables to be plotted and, eventually, the category for splitting plots, in the pull down menus
2. By default, density contours are active to feature the density of data points on the graph. This option should be deactivated for a small number of datapoints. When deactivated, a field appear where the categorical variable for coloring the datapoints can be chosen.
3. Press 'Update Parameters'
4. Press 'Generate Scatterplot', open the subtab 'Scatter Plot'
5. Adjust the plot parameters and formatting
   - Specific plot parameters (x, y axes) in the corresponding sub-Tab
   - Layout: set the size of the plots and the number of plots per raw
   - Themes: set the background color and presence/absence of gridlines
   - Colors: chose or customize the color palette
6. Once ready, the plots can be resized and downloaded ('Download Plots'), and the data subset used for the plots can be exported as .csv file ('Download Data')
7. plot's statistics are in the subtab 'Scatter Plot Data Table '

Adjust plot theme and colors

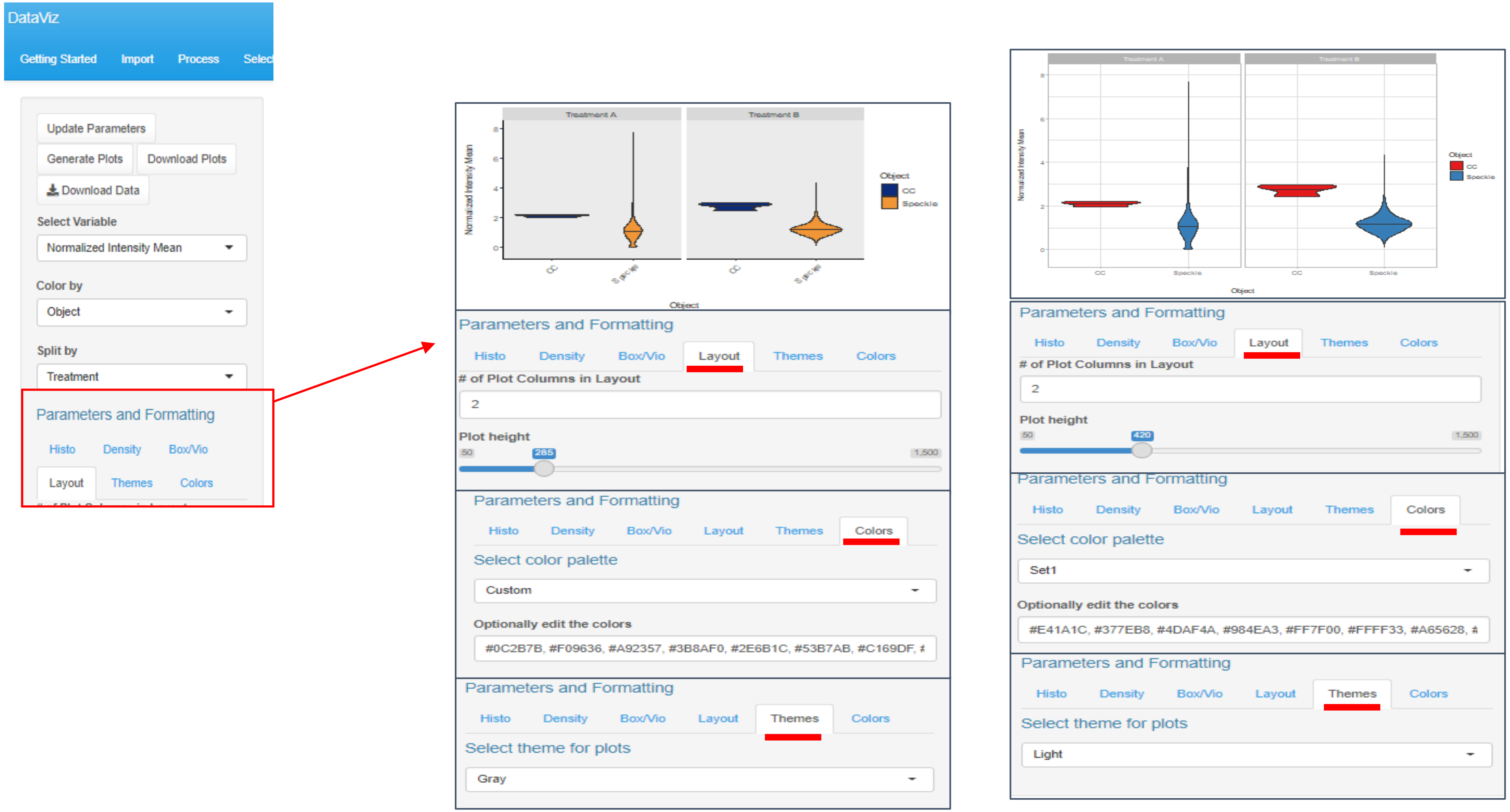

Sample Data, Data selected for CC, Speckles, Violin Plots, Split by: Treatment, Color by: Object type

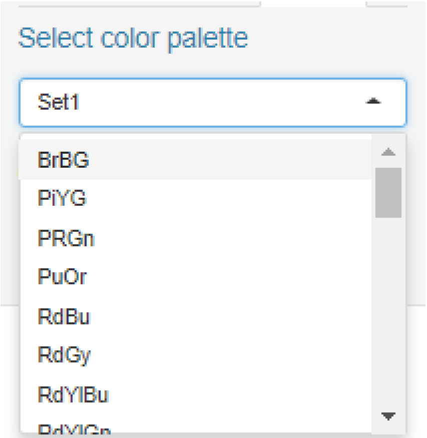

Define colors manually

- The palettes in the pull down menu come from the Color Brewer
- The default coloring is ‘Custom’ corresponding to the following set:

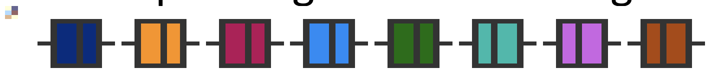

- To change manually the type and order of color, use the window with the heximal code:

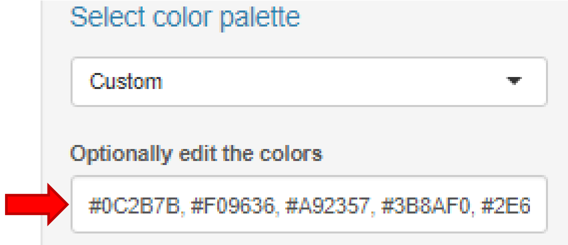

Color Brewer Available Palettes

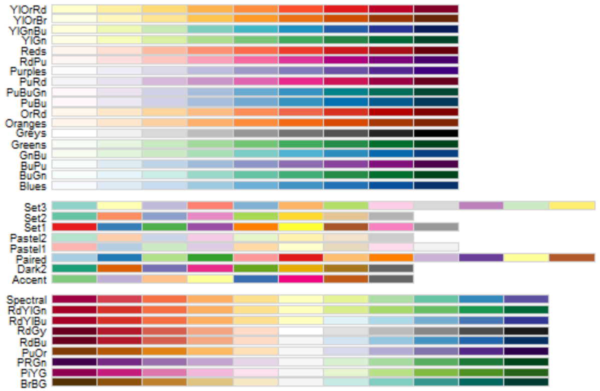

- Use https://encycolorpedia.com/ to find the heximal code of the desired color
- For instance to have neon green and fuschia copy paste #39ff14, #ff00ff in the ‘optionally edit the colors’ window at the beginning of the list (colors must be separated by a comma) – the colors should update automatically

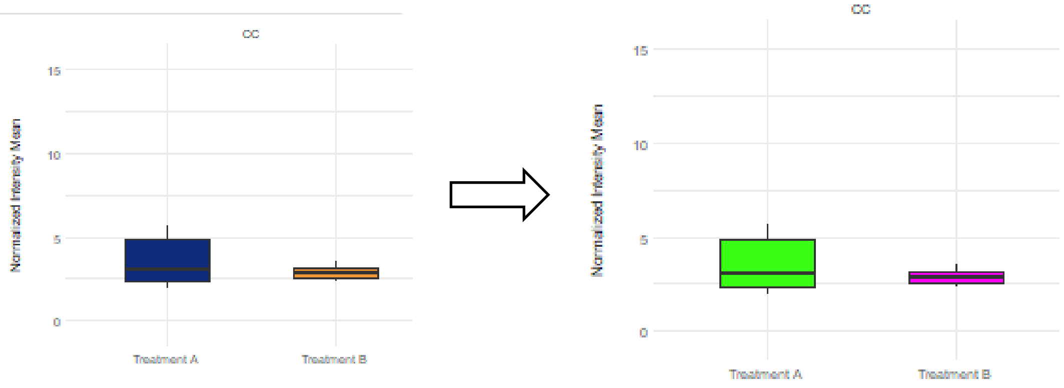

Plot per image file to verify data consistency/variability

Example 1: Distance of CC and speckles to nucleus periphery

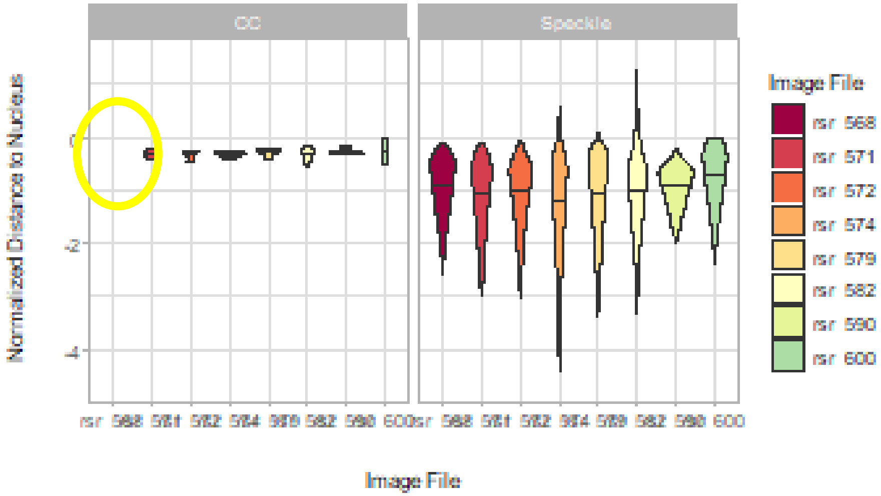

- In this example we can spot one image missing distance measurement for CC – this prompts to return to the image and activate the relevant statistics, then re-export the data

Example 2: Intensity Mean Ch=1 for Speckles

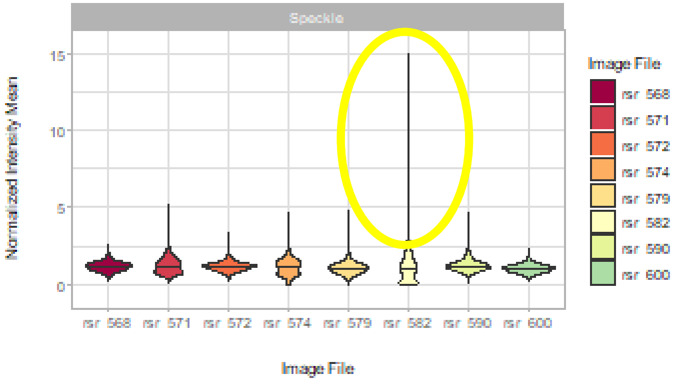

- In this example we can spot one image with highly variable and very high intensities (rsr582)– this prompts to return to the image and verify the spot segmentation, eventually curate and re-export the data

- Note: the color palettes have 9-11 colors. If more image files, the data will not be plotted (example error message – 'Insufficient values in manual scale. 8 needed but only 7 provided'). To solve this, copy the list of Hex code in Parameters/Colors/ editing window (see slide about colors) and paste it at the end of the same list, after adding a comma.

Filter Outliers

Before filtering

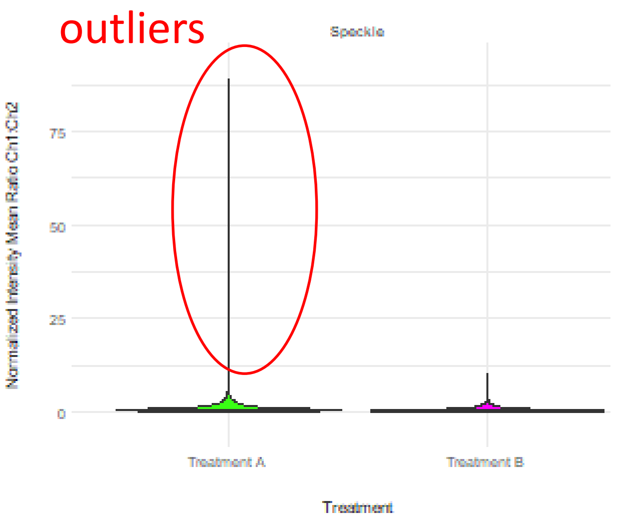

Object count (number of speckles before filtering)

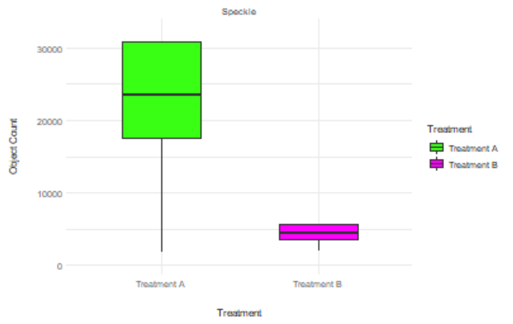

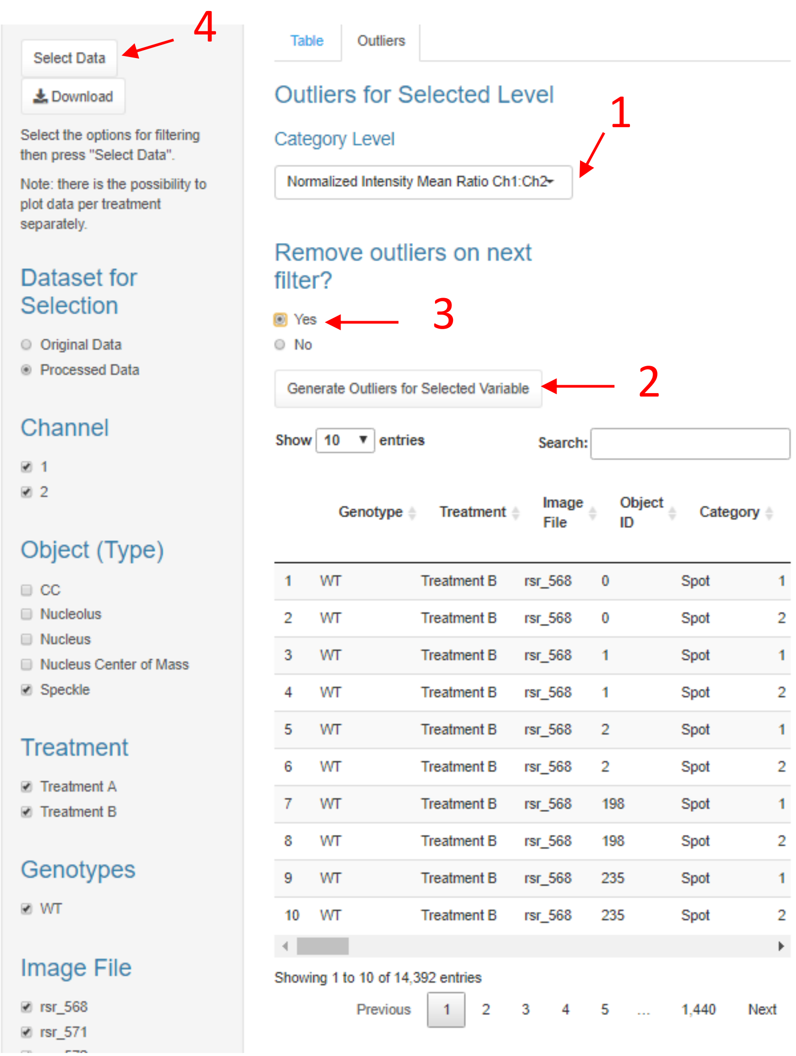

After filtering

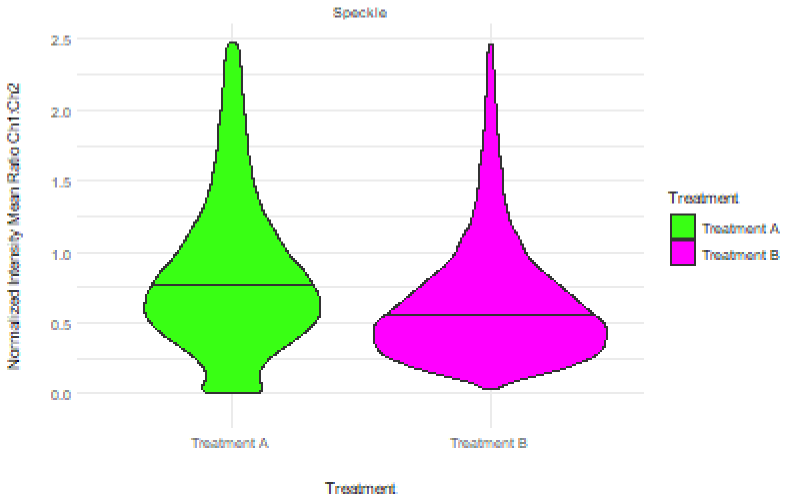

Object count (number of speckles after filtering)

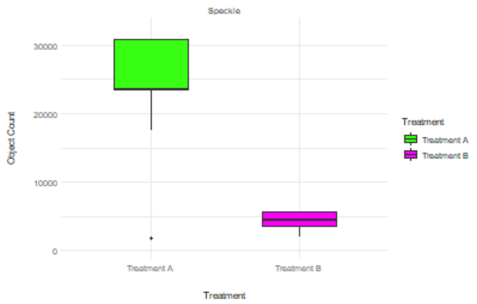

Adjust x and y limits

Example 1:

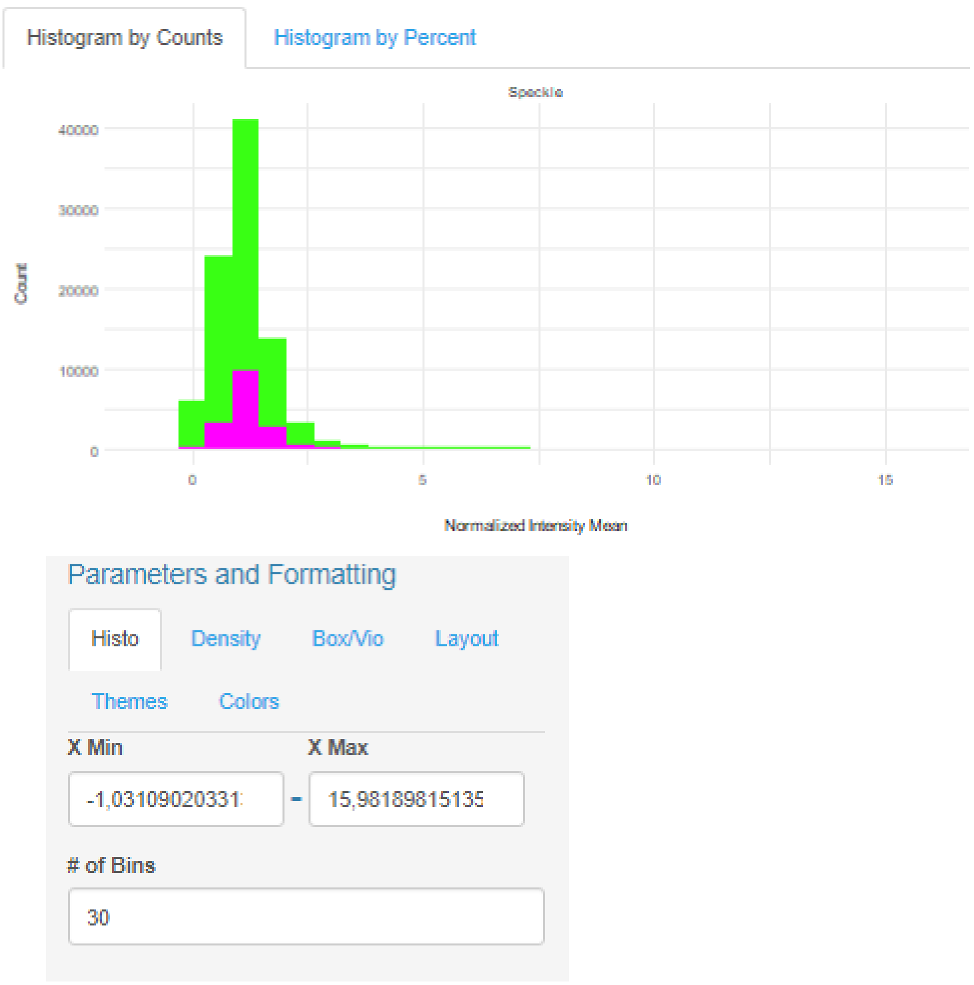

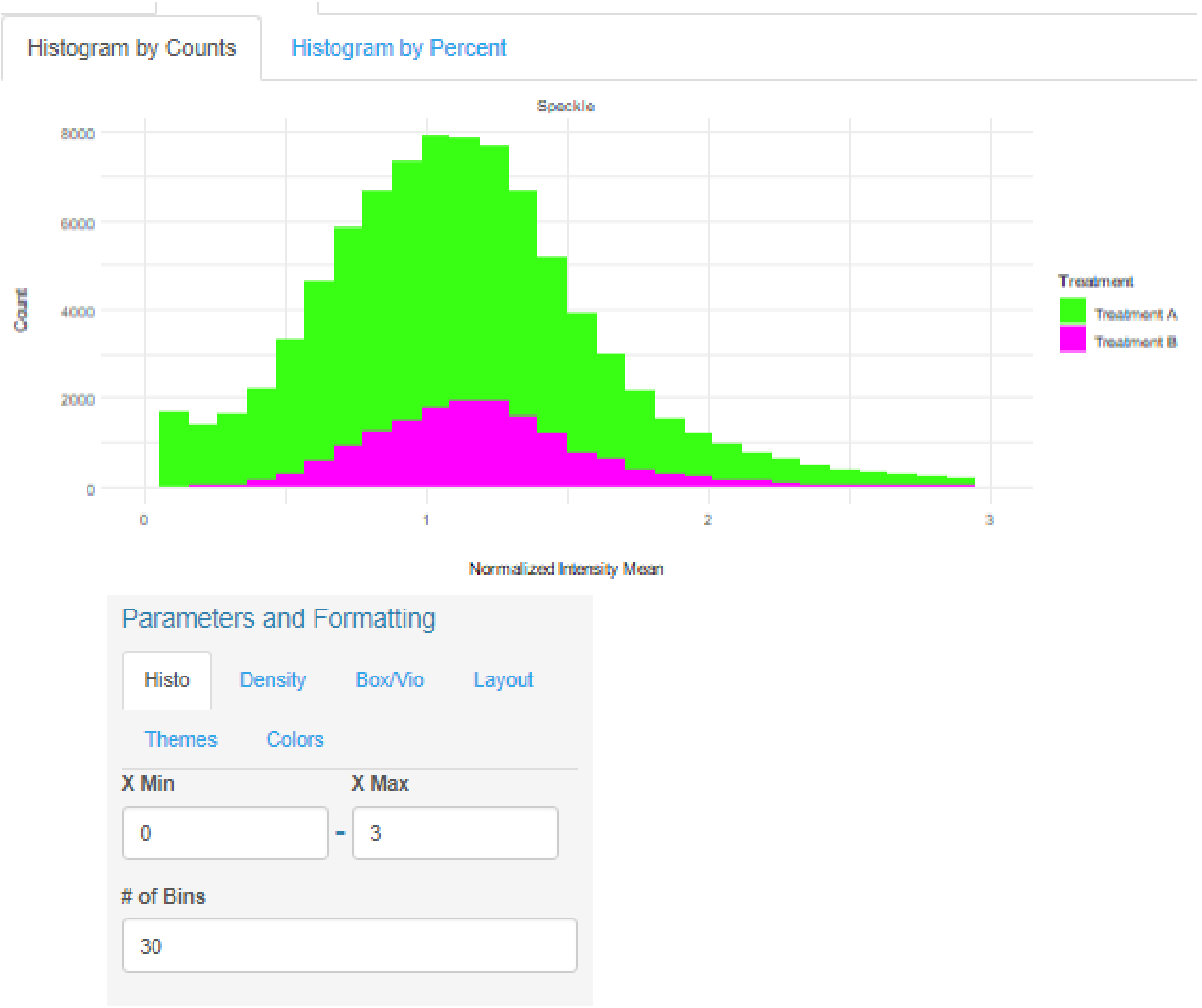

Adjust x and y limits

Example 2: Histogram (or KDE) plot packed on the left side of the graph -> reduce X Max

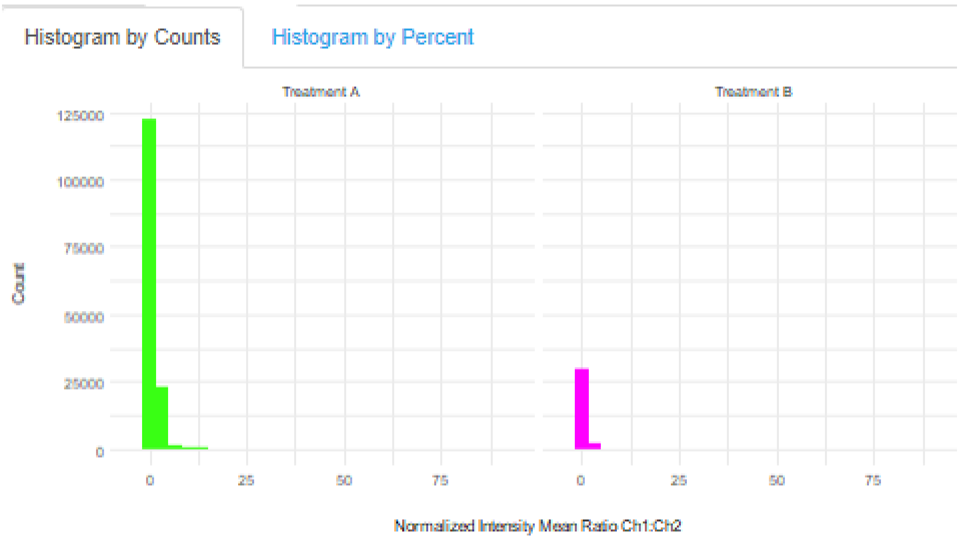

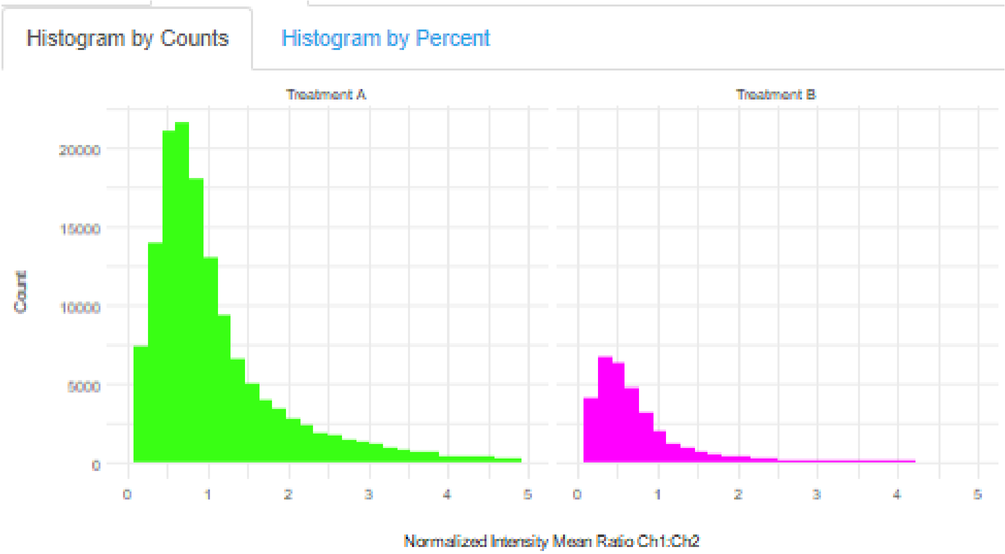

Note: this is a case where outliers should be removed as they influence the overall shape distribution. The violin plot (left) and the histogram (top) show that most values lay between 0 and 5, with outliers up to 80 : -> see section 'Filter outliers'

Adjust x and y limits

Example 3: Density distribution curve seems broken -> adjust the Y Max limit

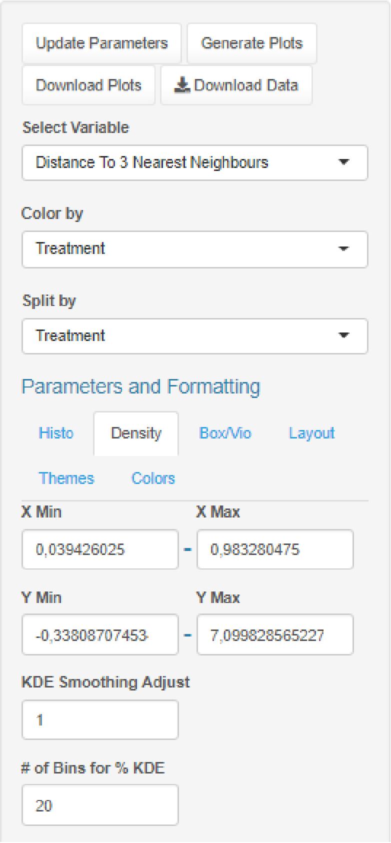

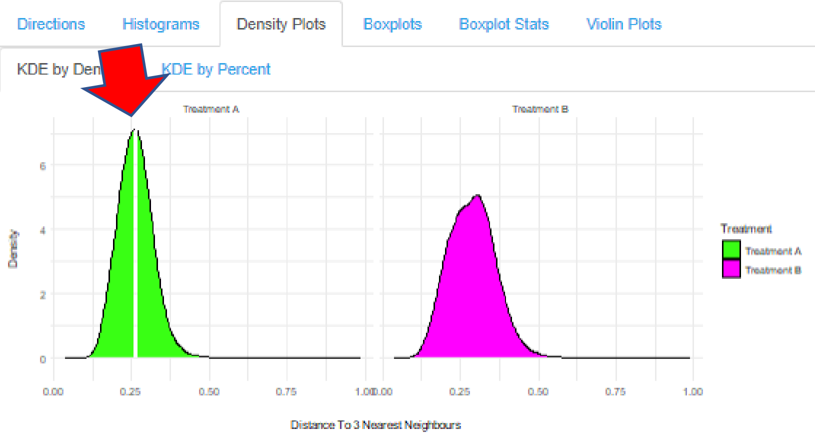

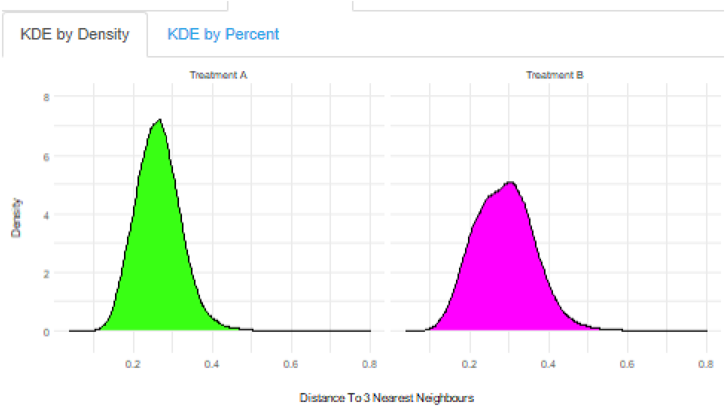

Y Max changed to 8

X Max changed to 0.8

Calculated parameters

Smooth histograms and KDEs

Example 1 : KDE / Histogram distribution show gross shapes -> adjust the number of bins

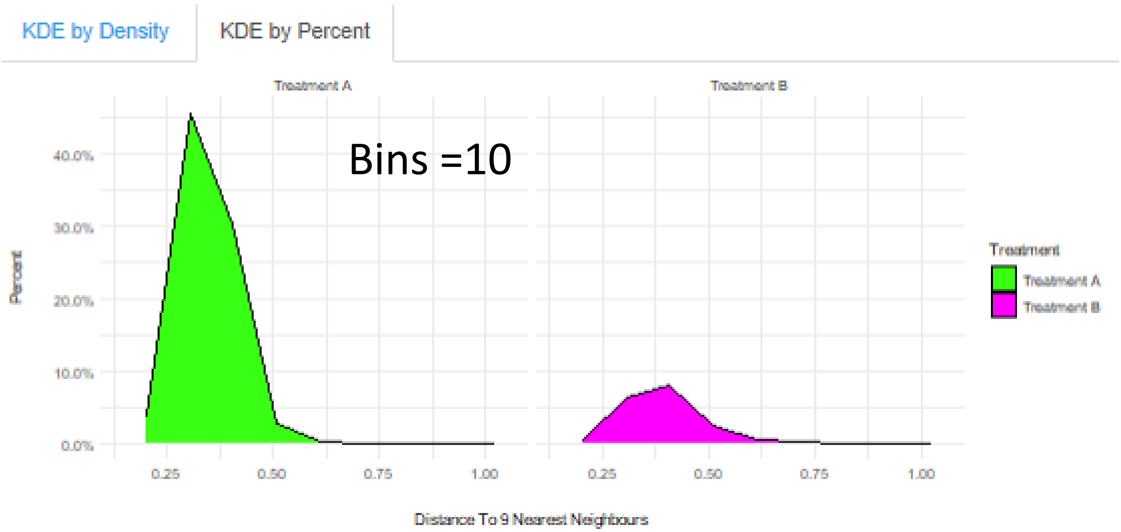

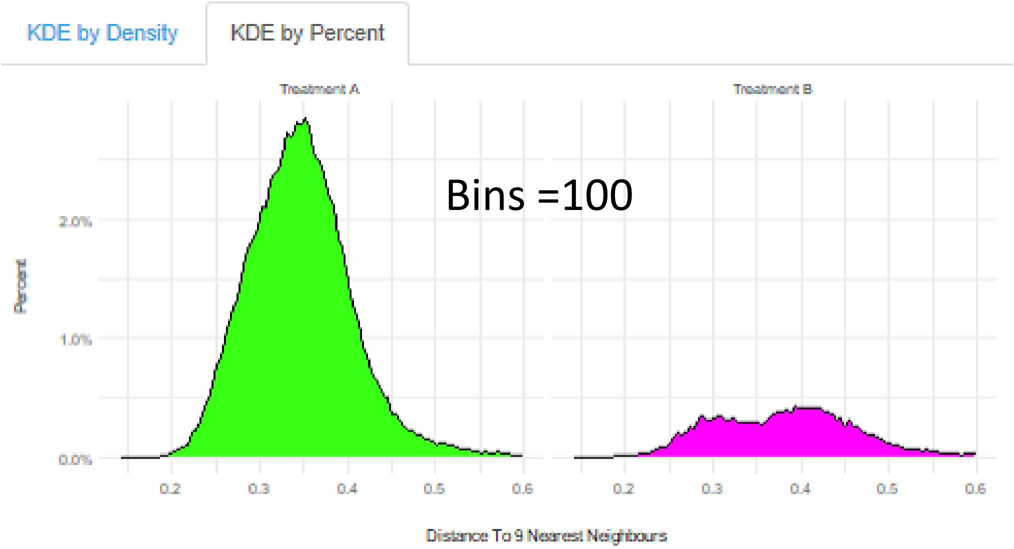

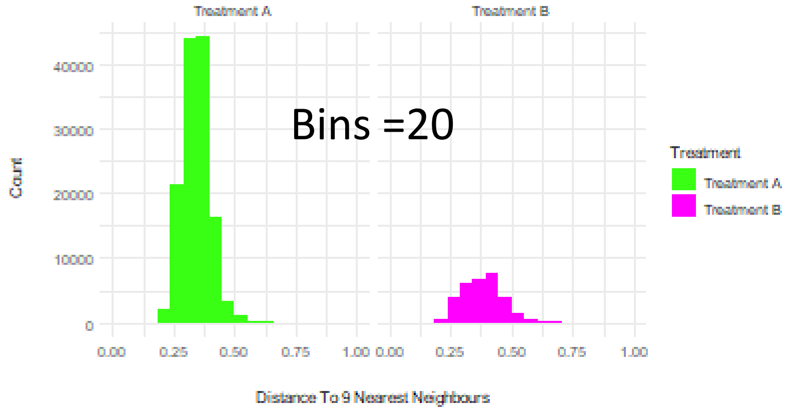

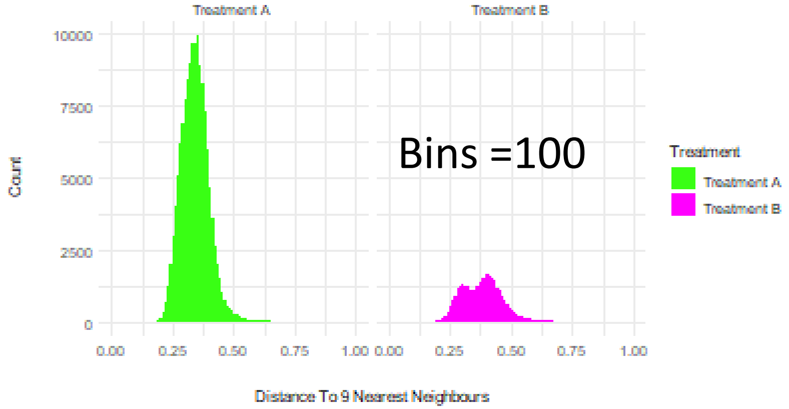

Smooth histograms and KDEs

Example 2: effect of smoothing on KDE distribution

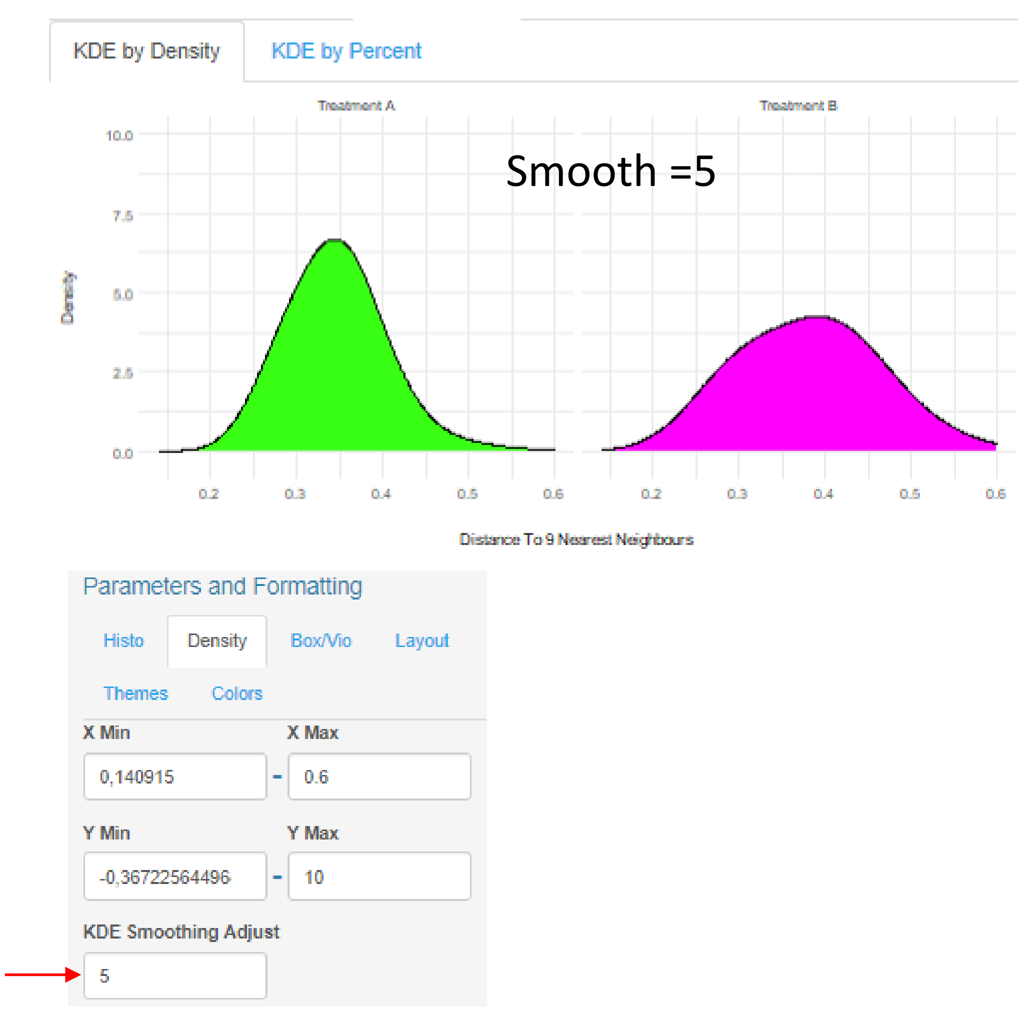

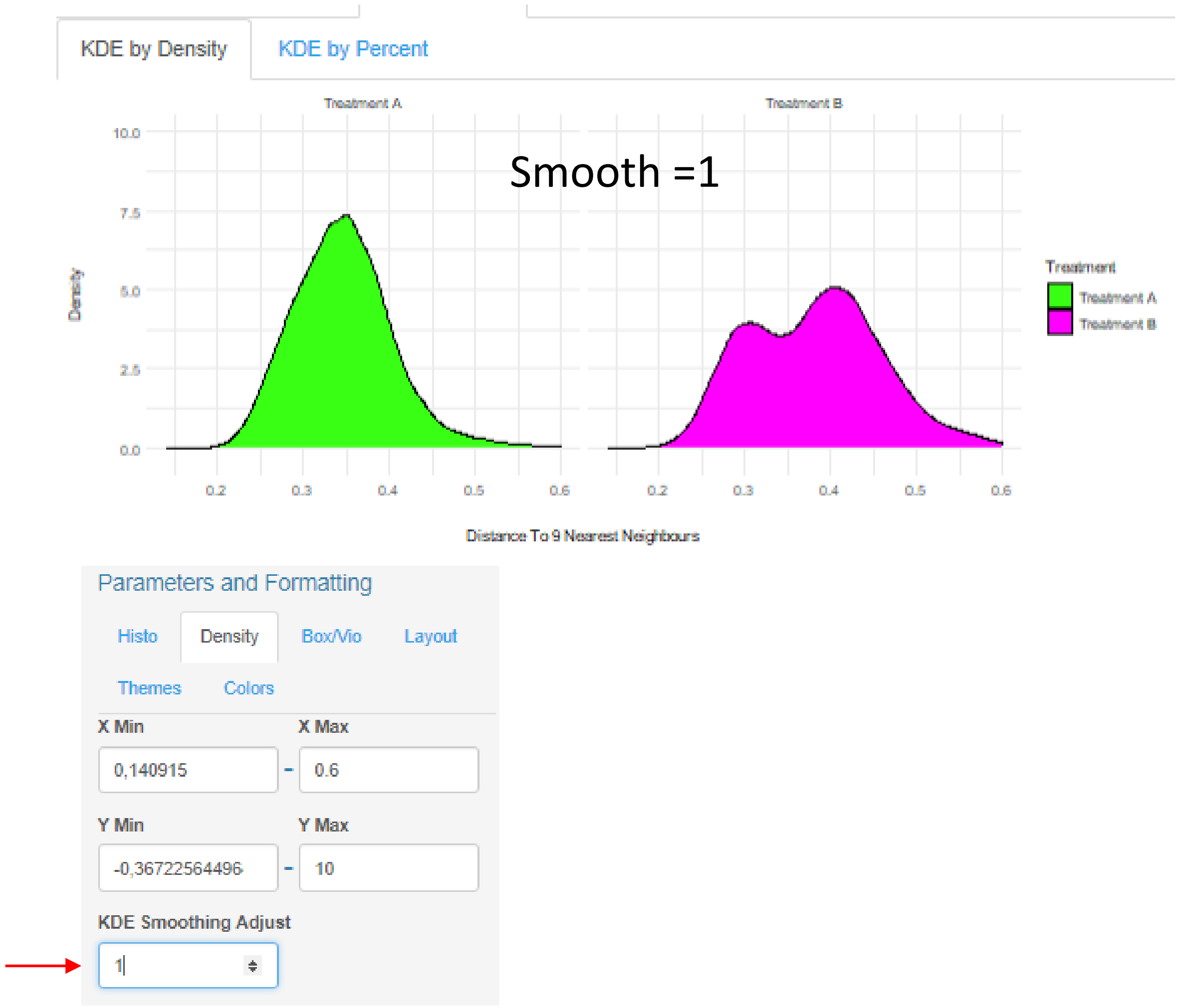

Data sub-setting: filter

In this example we select datapoints with value for ‘Distance to Nucleus’ (=nucleus periphery) between -0.2 and 0 (in µm)

Note that negative distances correspond to datapoints inside the surface (Nucleus). For other applications considering datapoints outside a surface, select a range with positive values

All data

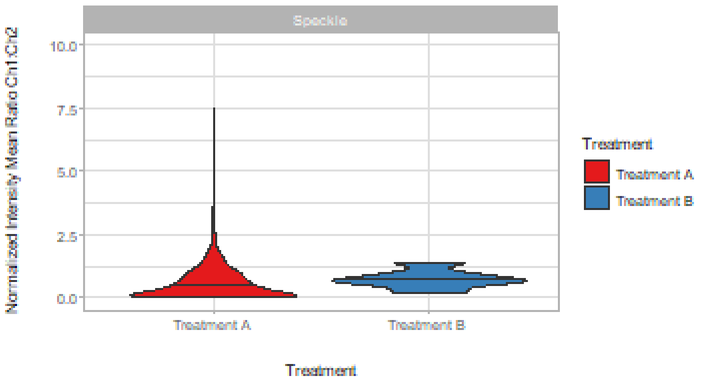

Channel ratio for all speckles

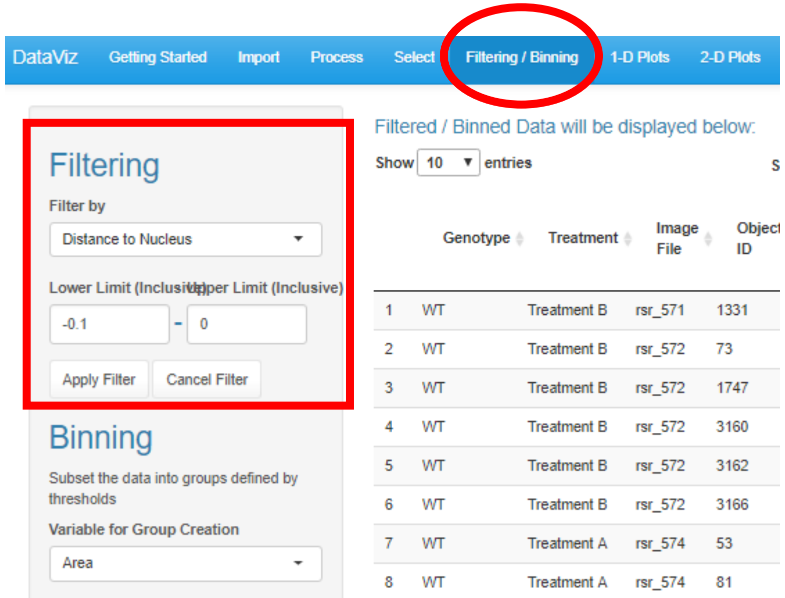

- Tab ‘Filtering/Binning’, Section ‘Filtering’
- Enter the range to subset
- Press ‘Apply filter’
- Return to the Plot Tab – press ‘Generate Plots’
- Data sub-setting can be reversed by applying ‘Cancel Filter’ and update the plots

Filtered data

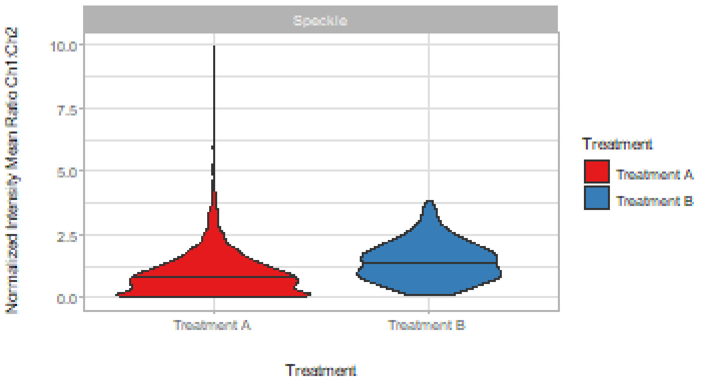

Channel ratio for speckles within a 200nm range of the nucleus periphery

This approach allows to focus on a specific set of data points focused on the biological question

Sample Data, selected for Speckles

Data sub-setting: create groups

In this example we create groups of datapoints located at different distance range from the Nucleus periphery

Note that negative distances correspond to datapoints inside the surface (Nucleus). For other applications considering datapoints outside a surface, select a range with positive values

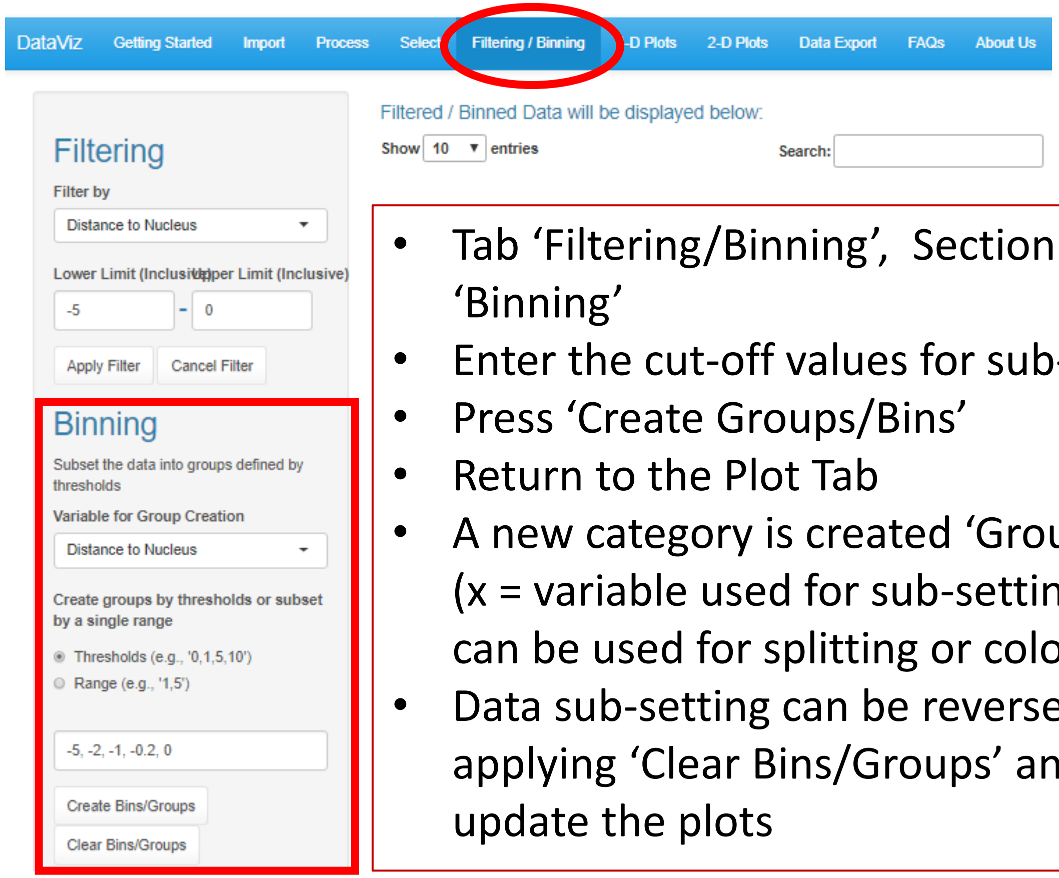

- Tab ‘Filtering/Binning’, Section ‘Binning’
- Enter the cut-off values for sub-setting
- Press ‘Create Groups/Bins’
- Return to the Plot Tab
- A new category is created ‘Group – x’ (x = variable used for sub-setting) that can be used for splitting or coloring
- Data sub-setting can be reversed by applying ‘Clear Bins/Groups’ and update the plots

Channel intensity ratio plotted as groups according to their distance to the nucleus periphery

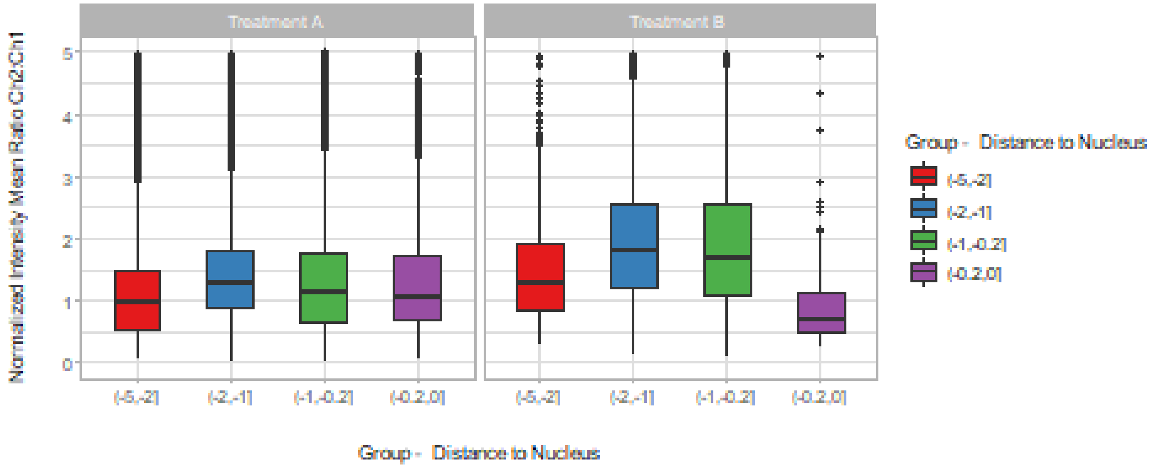

This approach reveals a group showing different intensity ratio between treatment (purple)
The data can be downloaded as csv file in the Plot area for statistical analyses

Sample Data, selected for Speckles

Scatter plot – datapoints or density contours

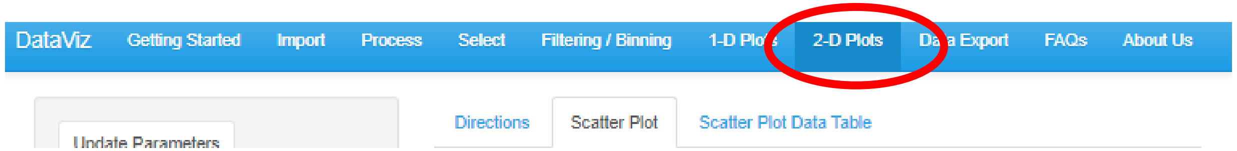

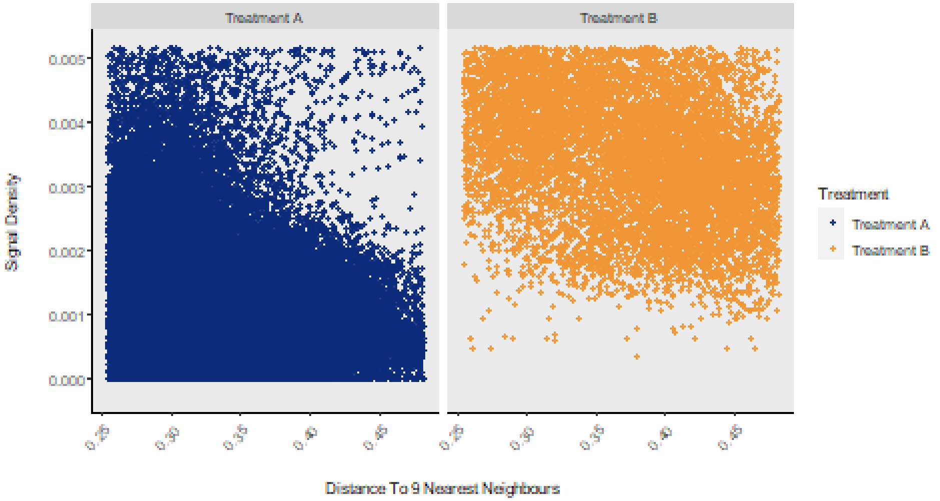

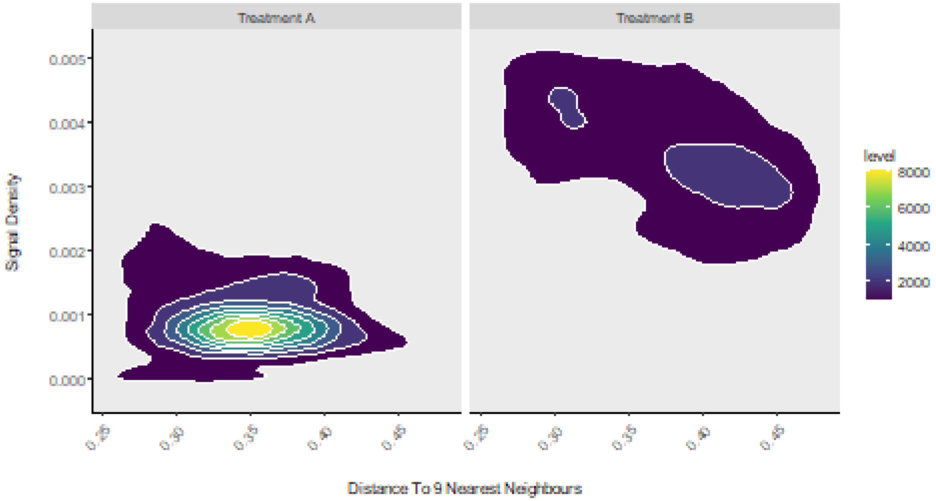

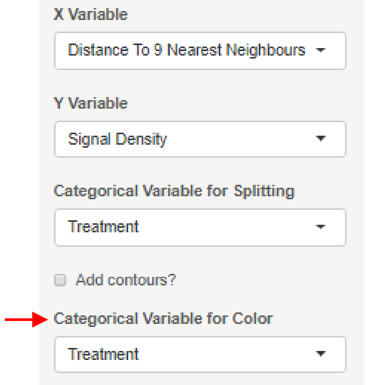

- In this example, the number of datapoints masks a possible heterogenous distribution

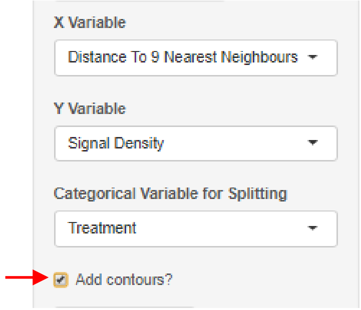

- density contours help identifying a different pattern relative to spacing between speckles (distance to 9 nearest neighbours) and signal density (intensity sum:volume ratio)
