# Supplementary figures and images for: Image analysis workflows to reveal the spatial organization of cell nuclei and chromosomes
Source: Nucleus. 2022 Nov 29;13(1):277–99. doi: 10.1080/19491034.2022.2144013 (PMC9754023; doi:10.1080/19491034.2022.2144013)

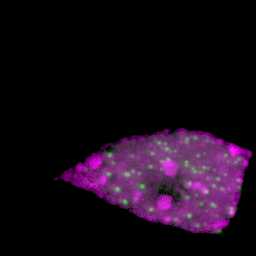

Supplement: Supplemental Material [file KNCL_A_2144013_SM9221.zip › Supplemental File 4 Text and Table/Supplemental File 4 - Text_and_Table/.imaris_cache/Image 4a.ims/thumb_256_0.jpg]

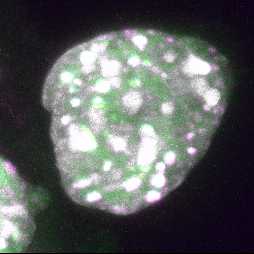

Supplement: Supplemental Material [file KNCL_A_2144013_SM9221.zip › Supplemental File 4 Text and Table/Supplemental File 4 - Text_and_Table/.imaris_cache/image 4b.ims/thumb_256_0.jpg]

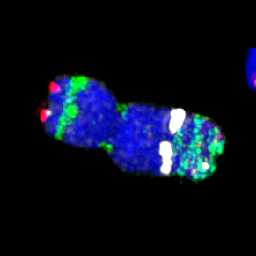

Supplement: Supplemental Material [file KNCL_A_2144013_SM9221.zip › Supplemental File 5 Text and Table/Supplemental File 5 - Text_and_Table/.imaris_cache/Image5.ims/thumb_256_0.jpg]

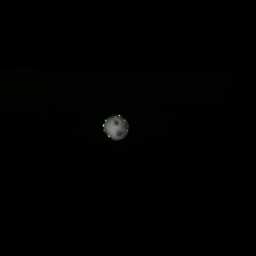

Supplement: Supplemental Material [file KNCL_A_2144013_SM9221.zip › Supplemental File 6 Text and Table/Supplemental File 6 - Text_and_Table/.imaris_cache/image6a_type I.ims/thumb_256_0.jpg]

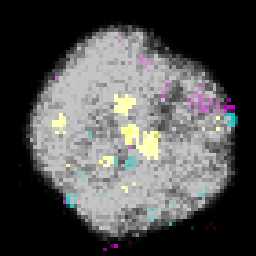

Supplement: Supplemental Material [file KNCL_A_2144013_SM9221.zip › Supplemental File 6 Text and Table/Supplemental File 6 - Text_and_Table/.imaris_cache/image6b.ims/thumb_256_0.jpg]

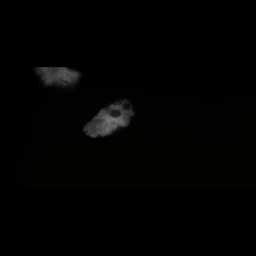

Supplement: Supplemental Material [file KNCL_A_2144013_SM9221.zip › Supplemental File 6 Text and Table/Supplemental File 6 - Text_and_Table/.imaris_cache/image6b_type II.ims/thumb_256_0.jpg]
